# Disease and Economic Burden of Intellectual Developmental Disability Attributable to Congenital Heart Disease, 1990–2021

**DOI:** 10.5334/gh.1511

**Published:** 2026-01-16

**Authors:** Xinjie Lin, Qiyu He, Xuyan Pei, Yanshang Wang, Sirui Zhou, Li Chen, Kai Ma, Zheng Dou, Yuze Liu, Yanbing Ma, Shoujun Li

**Affiliations:** 1 Pediatric Cardiac Surgery Center, State Key Laboratory of Cardiovascular Disease, National Clinical Research Center for Cardiovascular Diseases, Fuwai Hospital, Chinese Academy of Medical Sciences & Peking Union Medical College/National Center for Cardiovascular Diseases, Beijing 100037, China; 2 Division of Prevention and Community Health, National Center for Cardiovascular Disease, National Clinical Research center of Cardiovascular Disease, State Key Laboratory of Cardiovascular Disease, Fuwai Hospital, Peking Union Medical College & Chinese Academy of Medical Sciences, Beijing 102308, China; 3 Division of Psychiatry, Faculty of Brain Science, University College London, London WC1E 6BT, England; 4 Center of Laboratory Medicine, State Key Laboratory of Cardiovascular Disease, Beijing Key Laboratory for Molecular Diagnostics of Cardiovascular Diseases, National Clinical Research Center for Cardiovascular Diseases, Fuwai Hospital, Chinese Academy of Medical Sciences & Peking Union Medical College/National Center for Cardiovascular Diseases, Beijing 100037, China; 5 Institute of Child and Adolescent Health, School of Public Health, Peking University, Beijing, 100083, China

**Keywords:** Congenital heart disease, Intellectual developmental disability, Global burden of disease study

## Abstract

**Objective:**

Progressed medical techniques improved the life expectancy of congenital heart disease (CHD) population. Intellectual developmental disability (IDD) has progressively been a raised concern. This study aimed to comprehensively analyze the global burden, regional and age-specific differences, temporal trends, and economic cost of IDD attributable to CHD.

**Methods:**

This study was a secondary analysis of the Global Burden of Disease Study 2021 and World Development Indicators. The burden of IDD attributable to CHD was evaluated with prevalence, disability-adjusted life years, and estimated annual percentage change across global, socioeconomic, geographic, and age-specific subgroups. Joinpoint regression models were used to describe the temporal trends. Economic cost models were developed to estimate both direct and indirect costs.

**Results:**

In 2021, an estimated 1.05 million people lived with IDD attributable to CHD worldwide. Low-middle social-demographic index (SDI) regions were mostly affected. South Asia experienced the highest prevalence (0.30 million) among all geographic subregions. Children under the age of five were more susceptible to IDD attributable to CHD. The temporal trends varied across different SDI regions and age subgroups. The health-related expenditure of direct costs was disproportional with the burden of IDD attributable to CHD, which also contributed to a substantial income loss in the future.

**Conclusions and Policy Implications:**

Socioeconomic disadvantage and younger age are associated with a higher burden of IDD attributable to CHD. Efforts for both reducing CHD mortality and improving neurodevelopmental outcomes should be coordinately allocated.

## Introduction

Achievements in medical techniques have significantly improved the overall survival of congenital heart disease (CHD) over recent decades (1). From 1990 to 2019, the global mortality rate of CHD decreased by 60.4%, yet it remains the leading cause of congenital malformation, with 3.12 million live births and 13.3 million patients worldwide in 2019 (2). Reduced mortality has extended the lifespan of CHD patients, but prolonged survival has brought growing concern about non-fatal complications that pose long-term challenges to both personal quality of life and socioeconomic burden (3,4).

Developmental disabilities encompass a wide range of delayed attainment of developmental milestones (5,6). Thereinto, intellectual developmental disability (IDD) represents the most enduring and impactful sequela (6). Patients with CHD were reported to have an approximately ninefold higher risk of IDD compared to their non-CHD siblings, and the severity of intellectual impairment was parallel with the complexity of CHD subtypes (7,8). Furthermore, IDD persistently undermined cognition and daily functioning in CHD patients, and the growing number of CHD survivors further aggravated economic pressure on society (9,10,11). Current epidemiological studies are largely restricted to specific socioeconomic groups and age ranges (7,11,12). Although the Global Burden of Disease (GBD) study provides extensive health data across multiple countries and territories from 1990 to 2021, secondary analyses specifically addressing the disease and economic burden of IDD attributable to CHD remain scarce (13).

This study aimed to assess the global burden, regional and age disparities, temporal trends, and economic cost of IDD attributable to CHD using publicly available data from the GBD study 2021 and World Development Indicators. Our findings may address the gaps in epidemiological and health-economic evidence, providing valuable insights into policy development, resource allocation, and long-term caring strategies.

## Methods

### Overview

This secondary analysis of GBD Study 2021 assessed the global burden, regional and age disparities, temporal trends, and economic cost of IDD attributable to CHD. Disease data were downloaded from the Institute for Health Metrics and Evaluation (IHME, query tool: https://ghdx.healthdata.org/gbd-2021). Economic data were downloaded from the DataBank (World Development Indicators: https://databank.worldbank.org/source/world-development-indicators). The study consisted of four aspects: First, assessing the global and regional burden by reporting all-aged number and age-standardized rates of prevalence and disability-adjusted life years (DALYs) in 1990 and 2021, and calculating their estimated annual percentage change (EAPC) from 1990 to 2021 across global, both sexes, 5 SDI regions, 7 geographic regions, and 204 countries and territories; Second, visualizing the prevalence and DALYs of five severity levels of IDD attributable to CHD in both sexes and eight critical age subgroups; Third, analyzing temporal trends in prevalence and DALYs across age and SDI subgroups; Fourth, estimating the direct and indirect costs of IDD attributable to CHD across 204 countries and territories in 2021.

### Metric of burden and economic cost

Prevalence, DALYs, and the EAPC were used to measure the disease burden of IDD attributable to CHD. Prevalence was defined as the number of cases or the rate of IDD attributable to CHD in a given population and time point. The DALYs were represented by years lived with disability (YLD) due to the non-fatal nature of IDD as previously reported (14). The EAPC was calculated based on the methods proposed by Hankey et al. (15). In addition, the annual percent change (APC) and average annual percent change (AAPC) were used to quantify the annual percentage change in joinpoint regression models. Additionally, the economic cost of IDD attributable to CHD was estimated using direct and indirect costs. Direct costs reflected the health-associated expenses for IDD supported by families, societies, and governmental health systems. Indirect costs were used to assess future economic loss due to intellectual impairment. All cost values were presented as 2021 US dollars. Detailed definitions and descriptions were documented in the Supplementary Appendix (eAppendix 1).

### Statistical analysis

All-aged number and age-standardized rates of prevalence and DALYs with a 95% uncertainty interval (UI) were utilized to describe the burden. The 95%UI was estimated by selecting the 25th and 975th ordinals from 1000 draws of the posterior distribution at each step of the burden estimation process (16). The calculated EAPC with a 95% confidence interval (CI) was utilized to describe the yearly change from 1990 to 2021. Global regions were profiled to depict the regional differences, with the cut-off values for prevalence, DALYs, and economic cost being set at minimum, 10th, 25th, 50th, 75th, 90th quantiles, and the maximum as well as the cut-off values for EAPC being set at the minimum, 75th and 25th quantiles of all negative values, zero, 25th, and 75th quantiles of all positive values, and the maximum. Bidirectional stacked bar plots were used to visualize the age-specific discrepancies in both sexes, five hierarchical levels of IDD, and eight age-specific subgroups. Joinpoint regression analysis was employed to delineate temporal trends from 1990 to 2021, which quantitatively identified significant inflection joinpoints within the time series data and segmented the overall trend into discrete intervals demarcated by these joinpoints. Briefly, the direct costs were estimated following a method described by Chen et al. (17), based on the reported health-associated expenditures in the United States and adjusted by personal health expenditure across 204 countries and territories; the indirect costs were measured using a conventional strategy, converting lifelong income loss due to intelligence quotient (IQ) impairment into present value in 2021 (18). Information on modeling details was documented in the Supplementary Appendix (eAppendix 2). All data were analyzed and visualized using R (version 4.2.2) and Joinpoint (version 5.2.0) (19), with two-sided *P* < 0.05 being statistical significance.

## Results

### Global burden

In 2021, approximately 1.05 million people (95%UI: 0.83, 1.24) and 15.71 individuals (95%UI: 12.36, 18.58) per 100,000 population lived with IDD attributable to CHD worldwide. The global prevalence decreased from 1990 to 2021, with an EAPC of –0.15 (95%CI: –0.16, –0.13; Table 1). The global burden of IDD attributable to CHD was measured at 36.03 thousand DALYs (95%UI: 23.56, 53.97) and 0.53 DALYs (95%UI: 0.35, 0.8) per 100,000 population. The global DALYs also decreased from 1990 to 2021, with an EAPC of –0.35 (95%CI: –0.39, –0.31; Table 2).

**Table 1:** The prevalence in all-aged number and age-standardized rates and EAPC of IDD attributable to CHD from 1990 to 2021.


	1990		2021		1990, 2021
		
	NUMBER	ASPR PER 100,000	NUMBER	ASPR PER 100,000	EAPC
		
	NO.(95%UI)	NO.(95%UI)	NO.(95%UI)	NO.(95%UI)	NO.(95%CI)

**Global**	1002537.73	16.46	1049580.35	15.71	–0.15

	(811930.51, 1182553.62)	(13.37, 19.41)	(827445.59, 1240229.06)	(12.36, 18.58)	(–0.16, –0.13)

**Sexes**					

Male	513943.45	16.37	532748.35	15.52	–0.21

	(396318.50, 612334.24)	(12.62, 19.47)	(385978.16, 634998.34)	(11.20, 18.51)	(–0.23, –0.18)

Female	488594.28	16.54	516832.01	15.90	–0.09

	(402710.53, 576742.50)	(13.67, 19.47)	(423295.83, 608027.42)	(12.95, 18.76)	(–0.12, –0.06)

**SDI regions**					

Low SDI	149796.67	17.06	260790.53	16.31	–0.17

	(119233.36, 182693.05)	(13.73, 20.68)	(209008.92, 317620.92)	(13.12, 19.81)	(–0.20, –0.13)

Low-middle SDI	297693.01	17.83	325742.82	17.05	–0.10

	(245028.59, 357986.02)	(14.72, 21.33)	(268253.40, 385252.99)	(14.02, 20.19)	(–0.13, –0.07)

Middle SDI	317594.27	16.15	279260.21	15.20	–0.19

	(258423.84, 373609.97)	(13.18, 18.99)	(219974.67, 328415.93)	(11.88, 17.88)	(–0.20, –0.17)

High-middle SDI	146087.19	15.50	110225.35	14.37	–0.26

	(102028.96, 174528.50)	(10.71, 18.53)	(68911.32, 131221.02)	(8.36, 17.18)	(–0.27, –0.25)

High SDI	90570.27	13.96	72795.31	12.26	–0.40

	(41756.98, 110459.05)	(6.24, 17.10)	(30104.98, 91007.84)	(4.74, 15.38)	(–0.42, –0.38)

**Central Europe, Eastern Europe, and Central Asia**					

Central Asia	21330.47	23.31	23420.43	23.64	–0.10

	(16735.42, 26233.41)	(18.34, 28.54)	(15800.79, 28621.09)	(15.98, 28.87)	(–0.16, –0.04)

Central Europe	16822.77	17.89	9775.07	16.49	–0.39

	(10490.13, 20321.48)	(10.96, 21.67)	(3275.42, 12078.44)	(5.01, 20.45)	(–0.43, –0.35)

Eastern Europe	30288.43	17.21	18349.22	16.67	–0.24

	(15330.53, 36972.46)	(8.47, 21.15)	(8978.69, 22462.09)	(7.51, 20.58)	(–0.32, –0.17)

**High-income**					

Australasia	1228.86	7.81	1977.83	10.33	0.91

	(50.37, 1919.63)	(0.27, 12.28)	(709.34, 2707.04)	(3.49, 14.22)	(0.69, 1.13)

High-income Asia Pacific	13692.12	12.25	3831.45	4.99	–2.63

	(2175.70, 18792.86)	(1.59, 16.77)	(92.30, 9172.51)	(0.08, 12.30)	(–2.80, –2.46)

High-income North America	29480.48	13.29	27740.47	12.75	–0.13

	(14493.47, 35609.18)	(6.46, 16.04)	(11599.29, 33575.74)	(5.11, 15.49)	(–0.17, –0.09)

Southern Latin America	6486.64	12.70	5494.79	12.41	–0.16

	(4311.16, 7760.74)	(8.43, 15.18)	(2413.07, 6796.30)	(5.23, 15.31)	(–0.22, –0.11)

Western Europe	40670.84	15.91	35786.54	14.58	–0.25

	(23984.35, 48872.76)	(8.87, 19.30)	(18976.02, 43572.10)	(7.03, 17.97)	(–0.29, –0.21)

**Latin America and Caribbean**					

Andean Latin America	6851.93	13.54	8166.22	13.14	–0.12

	(5494.05, 8242.19)	(10.85, 16.24)	(5349.00, 9924.15)	(8.51, 15.98)	(–0.13, –0.10)

Caribbean	5330.95	13.13	5050.24	12.76	–0.14

	(3911.42, 6438.10)	(9.63, 15.84)	(3695.82, 6009.15)	(9.27, 15.17)	(–0.17, –0.12)

Central Latin America	30409.96	13.80	28151.34	13.54	–0.10

	(16627.91, 36667.55)	(7.79, 16.64)	(13220.59, 34158.83)	(6.21, 16.39)	(–0.12, –0.08)

Tropical Latin America	22591.53	13.49	22009.09	12.38	–0.28

	(17641.57, 26288.39)	(10.52, 15.70)	(14703.89, 26234.99)	(8.14, 14.76)	(–0.34, –0.23)

**North Africa and Middle East**					

North Africa and Middle East	91390.61	19.12	118705.17	19.46	0.00

	(76169.56, 106795.40)	(15.99, 22.33)	(98242.55, 137987.38)	(16.10, 22.61)	(–0.01, 0.02)

**South Asia**					

South Asia	284494.71	18.76	302255.84	18.78	0.12

	(234033.45, 343653.84)	(15.47, 22.59)	(252659.00, 357444.65)	(15.64, 22.30)	(0.07, 0.17)

**Southeast Asia, East Asia, and Oceania**					

East Asia	175214.26	15.03	114036.91	13.24	–0.41

	(134038.04, 209492.23)	(11.48, 18)	(72492.02, 136829.39)	(7.80, 15.95)	(–0.42, –0.40)

Oceania	1399.52	14.41	2703.74	14.32	0.01

	(1119.23, 1724.1)	(11.52, 17.71)	(2168.99, 3304.33)	(11.57, 17.48)	(0.00, 0.03)

Southeast Asia	269427.98	15.44	198567.55	13.70	–0.41

	(217613.29, 319857.45)	(12.47, 18.33)	(147363.99, 236391.39)	(9.95, 16.35)	(–0.43, –0.40)

**Sub-Saharan Africa**					

Central Sub-Saharan Africa	16246.22	16.08	31753.47	15.70	–0.15

	(12133.45, 20999.06)	(12.01, 20.69)	(24669.51, 39613.37)	(12.26, 19.46)	(–0.25, –0.05)

Eastern Sub-Saharan Africa	51790.63	14.87	84002.03	13.63	–0.37

	(41544.61, 62514.65)	(12.11, 17.8)	(65804.55, 102424.93)	(10.70, 16.58)	(–0.40, –0.33)

Southern Sub-Saharan Africa	9101.38	12.66	10168.28	12.69	–0.01

	(3580.61, 11254.39)	(5.08, 15.64)	(4284.54, 12483.75)	(5.30, 15.57)	(–0.02, 0.00)

Western Sub-Saharan Africa	54901.22	15.77	114375.34	14.81	–0.30

	(37501.04, 68432.67)	(10.94, 19.49)	(67482.28, 141019.43)	(8.84, 18.27)	(–0.34, –0.25)


Abbreviation: EAPC, estimated annual percentage change; IDD, intellectual disability developmental; CHD, congenital heart disease; ASPR, age-standardized prevalence rates; UI, uncertainty intervals; CI, confidence intervals; SDI, social demographic index.

**Table 2:** The DALYs in all-aged number and age-standardized rates and EAPC of IDD attributable to CHD from 1990 to 2021.


	1990		2021		1990–2021
		
	NUMBER	ASDR PER 100,000	NUMBER	ASDR PER 100,000	EAPC
		
	NO.(95%UI)	NO.(95%UI)	NO.(95%UI)	NO.(95%UI)	NO.(95%CI)

**Global**	35189.34	0.58	36029.73	0.53	–0.35

	(23049.83, 53052.22)	(0.38, 0.88)	(23561.33, 53971.97)	(0.35, 0.80)	(–0.39, –0.31)

**Sexes**					

Male	17371.71	0.56	17652.08	0.51	–0.41

	(11286.25, 26402.73)	(0.36, 0.85)	(11423.50, 26227.34)	(0.33, 0.76)	(–0.47, –0.35)

Female	17817.63	0.61	18377.66	0.56	–0.29

	(11626.80, 26826.93)	(0.40, 0.91)	(12070.61, 27001.62)	(0.36, 0.83)	(–0.32, –0.25)

**SDI regions**					

Low SDI	5319.67	0.62	8748.91	0.56	–0.47

	(3382.36, 8106.97)	(0.40, 0.95)	(5555.04, 13051.95)	(0.36, 0.83)	(–0.56, –0.39)

Low-middle SDI	12129.03	0.74	12554.82	0.66	–0.36

	(7812.45, 18064.54)	(0.47, 1.09)	(8173.63, 18321.98)	(0.43, 0.96)	(–0.40, –0.32)

Middle SDI	10966.54	0.56	9373.63	0.49	–0.44

	(7114.14, 16474.67	(0.36, 0.84)	(6103.31, 14033.70)	(0.32, 0.74)	(–0.46, –0.42)

High-middle SDI	4188.35	0.44	3252.76	0.39	–0.47

	(2545.11, 6433.86)	(0.27, 0.67)	(1939.47, 4977.61)	(0.23, 0.61)	(–0.51, –0.42)

High SDI	2561.48	0.38	2076.88	0.32	–0.55

	(1575.54, 3856.20)	(0.23, 0.57)	(1250.19, 3183.16)	(0.19, 0.49)	(–0.61, –0.50)

**Central Europe, Eastern Europe, and Central Asia**					

Central Asia	618.59	0.69	658.80	0.67	–0.38

	(359.13, 947.65)	(0.40, 1.05)	(364.27, 998.96)	(0.37, 1.01)	(–0.53, –0.22)

Central Europe	458.93	0.47	244.76	0.39	–0.84

	(254.10, 724.48)	(0.26, 0.76)	(121.30, 402.01)	(0.19, 0.65)	(–0.90, –0.78)

Eastern Europe	752.92	0.41	488.80	0.41	–0.34

	(402.64, 1224.68)	(0.22, 0.68)	(256.33, 780.35)	(0.21, 0.66)	(–0.52, –0.16)

**High-income**					

Australasia	19.23	0.12	51.42	0.25	2.49

	(3.64, 40.20)	(0.02, 0.25)	(26.51, 85.12)	(0.13, 0.43)	(2.01, 2.98)

High-income Asia Pacific	364.45	0.30	104.70	0.10	–3.31

	(153.80, 609.23)	(0.12, 0.50)	(6.21, 240.84)	(0.00, 0.25)	(–3.38, –3.23)

High-income North America	847.21	0.37	820.25	0.36	–0.14

	(546.3, 1283.54)	(0.24, 0.57)	(534.09, 1221.89)	(0.23, 0.54)	(–0.18, –0.10)

Southern Latin America	190.73	0.37	157.49	0.34	–0.44

	(119.48, 289.24)	(0.23, 0.57)	(99.81, 234.34)	(0.22, 0.51)	(–0.55, –0.33)

Western Europe	1307.60	0.47	1118.73	0.40	–0.49

	(810.90, 1944.45)	(0.29, 0.71)	(679.55, 1684.22)	(0.23, 0.62)	(–0.54, –0.44)

**Latin America and Caribbean**					

Andean Latin America	196.10	0.40	229.95	0.37	–0.34

	(113.06, 301.73)	(0.23, 0.61)	(122.57, 356.82)	(0.19, 0.57)	(–0.36, –0.32)

Caribbean	139.73	0.35	136.19	0.34	–0.17

	(68.59, 219.31)	(0.17, 0.55)	(70.19, 215.28)	(0.17, 0.54)	(–0.20, –0.14)

Central Latin America	769.76	0.36	723.71	0.34	–0.29

	(417.02, 1246.56)	(0.20, 0.58)	(376.24, 1167.37)	(0.17, 0.55)	(–0.36, –0.22)

Tropical Latin America	643.27	0.39	582.12	0.32	–0.69

	(379.71, 1002.05)	(0.23, 0.60)	(315.37, 897.37)	(0.17, 0.49)	(–0.77, –0.60)

**North Africa and Middle East**					

North Africa and Middle East	3486.28	0.75	4518.12	0.74	–0.18

	(2228.27, 5275.79)	(0.48, 1.13)	(2922.01, 6708.96)	(0.48, 1.10)	(–0.22, –0.14)

**South Asia**					

South Asia	12792.22	0.85	13674.81	0.84	0.07

	(8259.77, 18737.62)	(0.55, 1.24)	(8880.17, 19863.14)	(0.55, 1.22)	(0.01, 0.12)

**Southeast Asia, East Asia, and Oceania**					

East Asia	5101.82	0.43	3283.37	0.35	–0.80

	(2977.15, 7867.67)	(0.25, 0.67)	(1870.56, 5046.48)	(0.19, 0.55)	(–0.86, –0.75)

Oceania	42.45	0.45	79.41	0.43	–0.04

	(25.64, 66.86)	(0.27, 0.71)	(47.49, 121.79)	(0.26, 0.66)	(–0.08, –0.01)

Southeast Asia	3631.19	0.64	2594.60	0.44	–1.25

	(2355.78, 5378.91)	(0.42, 0.95)	(1669.25, 3901.14)	(0.28, 0.67)	(–1.30, –1.19)

**Sub-Saharan Africa**					

Central Sub-Saharan Africa	425.05	0.43	883.37	0.44	–0.14

	(238.38, 703.53)	(0.25, 0.71)	(507.97, 1456.81)	(0.26, 0.73)	(–0.42, 0.13)

Eastern Sub-Saharan Africa	1662.69	0.50	2441.84	0.40	–0.93

	(1027.54, 2590.90)	(0.31, 0.76)	(1452.09, 3747.40)	(0.24, 0.62)	(–1.03, –0.84)

Southern Sub-Saharan Africa	213.83	0.31	253.61	0.32	0.06

	(109.52, 354.79)	(0.16, 0.51)	(132.57, 406.14)	(0.16, 0.51)	(0.04, 0.08)

Western Sub-Saharan Africa	1525.27	0.46	2983.68	0.40	–0.67

	(894.79, 2442.61)	(0.27, 0.72)	(1646.61, 4747.87)	(0.22, 0.63)	(–0.77, –0.57)


Abbreviation: DALYs, disability–adjusted life years; EAPC, estimated annual percentage change; IDD, intellectual developmental disability; CHD, congenital heart disease; ASDR, age-standardized DALYs rates; UI, uncertainty intervals; CI, confidence intervals; SDI, social demographic index.

### Regional difference

The burden of IDD attributable to CHD was correlated with the SDI levels, with the highest prevalence of 0.32 million people (95%UI: 0.27, 0.38) and 17.05 individuals (95%UI: 14.02, 20.19) per 100,000 population in low-middle SDI regions compared to others in 2021. High SDI regions exhibited the highest prevalence reduction, with an EAPC of –0.4 (95%CI: –0.42, –0.38; Table 1). Moreover, low-middle SDI regions also experienced the highest DALYs, accounting for 12.55 thousand DALYs (95%UI: 8.17, 18.32) and 0.66 DALYs (95%UI: 0.43, 0.96) per 100,000 population. High SDI regions remained the highest DALYs declined regions from 1990 to 2021, with an EAPC of –0.55 (95%CI: –0.61, –0.5; Table 2).

**Figure 1:**
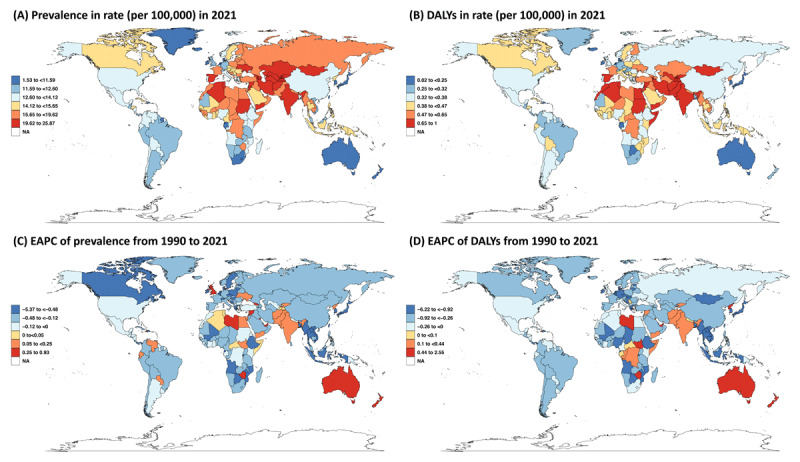
Global prevalence rates, DALYs rates, prevalence EAPC, and DALYs EAPC of IDD attributable to CHD across 204 countries and territories. **(A)** Prevalence rates (per 100,000) in 2021; **(B)** DALYs rates (per 100,000) in 2021; **(C)** EAPC of prevalence from 1990 to 2021; **(D)** EAPC of DALYs from 1990 to 2021. Abbreviations: DALYs, disability-adjusted life years; EAPC, estimated annual percentage change; IDD, intellectual developmental disability; CHD, congenital heart disease; NA, not available.

The burden of IDD attributable to CHD across geographical regions in 2021 is pictured in Figure 1. South Asia and Central Asia represented the most affected regions, with the highest prevalence number of 0.30 million people (95%UI: 0.25, 0.36) in South Asia and the highest prevalence rate of 23.64 individuals (95%UI: 15.98, 28.87) per 100,000 population in Central Asia. From 1990 to 2021, the prevalence decreased across most geographical regions except Australasia of an EAPC of 0.91 (95%CI: 0.69, 1.13) and South Asia of an EAPC of 0.12 (95%CI: 0.07, 0.17; Table 1). Nationally, 0.22 million (95%UI: 0.19, 0.26) people in India were affected by the IDD attributable to CHD, with 0.11 million (95%UI: 0.07, 0.13) people in China following behind. The prevalence rates were notably higher in Tajikistan of 25.87 individuals (95%UI: 20.47, 32.13) per 100,000 population, Armenia of 25.18 individuals (95%UI: 16.4, 30.77), and Mongolia of 24.91 individuals (95%UI: 16.92, 30.79) per 100,000 population. Australia exhibited the most pronounced annual increase, while Singapore experienced the steepest annual decline in IDD attributable to CHD prevalence from 1990 to 2021 (eAppendix 3, STable 3).

Additionally, South Asia remained the most affected region, with the highest number of 13.67 thousand DALYs (95%UI: 8.89, 19.86) and rates of 0.84 DALYs (95%UI: 0.55, 1.22) per 100,000 population in 2021. From 1990 to 2021, only Australasia, South Asia, and Southern Sub-Saharan Africa exhibited an increased DALYs (Table 2). Nationally, India continued to be the country heavily impacted, with 10.77 thousand DALYs (95%UI: 7.01, 15.68) and 0.93 DALYs (95%UI: 0.61, 1.36) per 100,000 population. Similarly, Australia recorded the highest annual increase in DALYs from 1990 to 2021, while South Korea experienced the greatest annual decrease over the same period (eAppendix 3, STable 4).

### Age-specific difference

**Figure 2:**
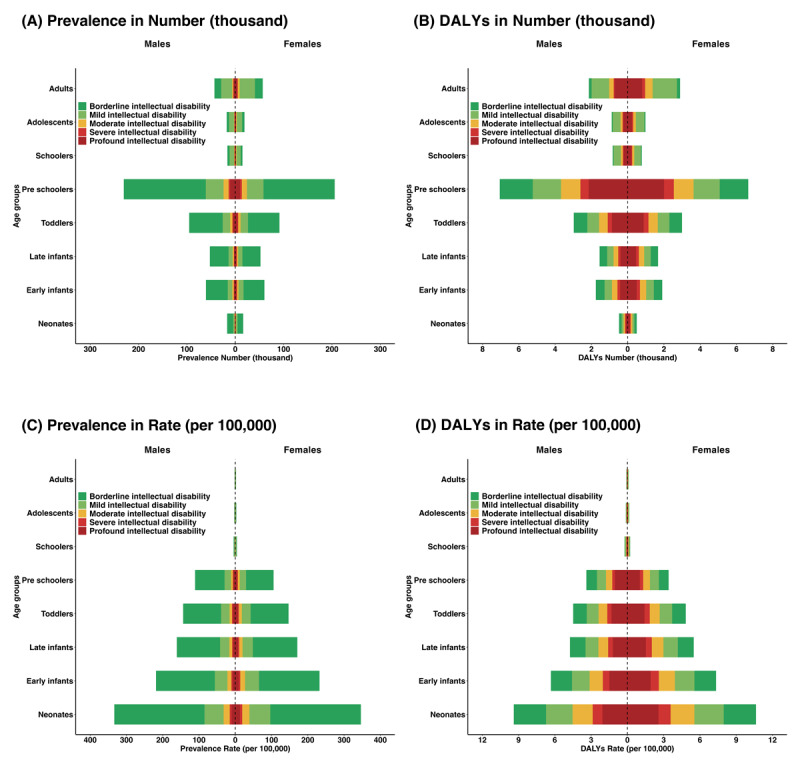
Global prevalence and DALY of different levels of IDD attributable to CHD across ten age subgroups and both sexes in 2021. **(A)** Prevalence number (thousand); **(B)** DALY number (thousand); **(C)** Prevalence rates (per 100,000); **(D)** DALY rates (per 100,000). Abbreviations: DALY, disability-adjusted life years; IDD, intellectual developmental disability; CHD, congenital heart disease.

The age-specific differences of IDD attributable to CHD burden are plotted in Figure 2. Children under the age of five were the most significantly affected population in 2021. Both the prevalence and DALYs number in preschoolers drastically outnumbered others, and the rate was higher in neonates. There was no remarkable discrepancy between the two sexes in the prevalence of five hierarchical levels of IDD attributable to CHD across age subgroups. More corresponding data of the global burden of different hierarchical levels of IDD attributable to CHD across sexes and age subgroups were documented in eAppendix 4.

### Temporal trends

The temporal trends of the prevalence number of IDD attributable to CHD varied across global and five SDI regions, with an S-curve pattern globally, a linear growth pattern in low SDI regions, a continuous decline in high SDI regions, and volatile changes in other SDI regions. This study also witnessed a consistent decrease in prevalence rate in high SDI regions and a fluctuating decrease in others (eAppendix 5, SFigure 1 and 2). The temporal trends of the DALYs across global and SDI regions have a similar tendency (eAppendix 5, SFigure 3 and 4). More detailed data of APC and AAPC globally and across five SDI regions were documented in eAppendix 5 (STable 6).

The temporal change pattern of IDD attributable to CHD burden across eight age subgroups was different. Children under the age of five experienced a fluctuating increase in prevalence number before the year period of 2016 to 2019, but encountered a drastic decrease after that. This study also observed a volatile increase of prevalence in schoolers and adolescents as well as a consistently linear increase in adults from 1990 to 2021. Additionally, the prevalence rate decreased from 1990 to 2021 across all age subgroups with different decline patterns (eAppendix 5, SFigure 5 and 6). The temporal trends of DALYs across age subgroups were also delineated (eAppendix 5, SFigure 7 and 8). More detailed data of APC and AAPC across eight age subgroups were presented in eAppendix 5 (STable 7).

### Economic burden

**Figure 3:**
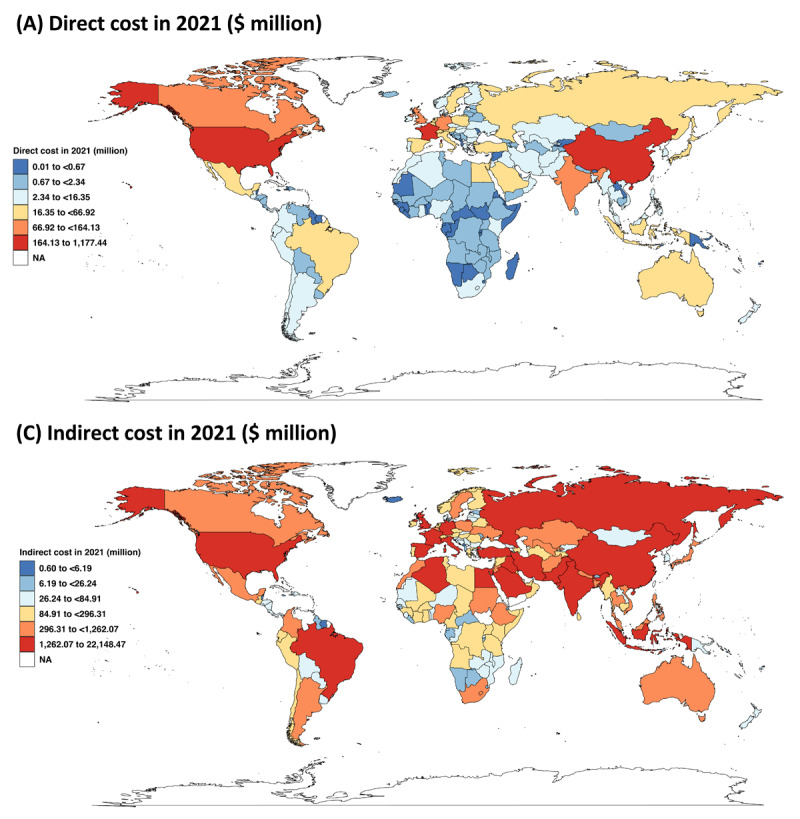
Economic cost of IDD attributable to CHD in 2021. **(A)** Direct costs in 2021 ($ million); **(B)** Indirect costs in 2021 ($ million). Abbreviations: IDD, intellectual developmental disability; CHD, congenital heart disease; NA, not available.

The direct costs of IDD attributable to CHD in 2021 significantly differed across 204 countries and territories, with the United States inputting the highest at $1,177.44 ($491.5 to $1,428.2) million, followed by China at $292.86 ($184.1 to $352.05) million. Indirect costs constituted the majority of total cost, with India having the highest at $22,148.47 ($16,627.36 to $27,937.39) million, followed by the United States at $10,067.13 ($7,133.32 to $13,657.21) and China at $9,315.27 ($4,461.09 to $13,452.92; Figure 3). More data of the cost analysis were documented in the eAppendix 6 (STable 8).

## Discussion

To our best knowledge, this is the first study that collectively described the burden and cost of IDD attributable to CHD at the population level. The significant contribution of this study lies in replenishing epidemiological and health-economic evidence on the neurodevelopment issue associated with CHD. Major findings are presented: (1) Globally in 2021, 1.05 million people (15.71 individuals per 100,000 population) lived with IDD attributable to CHD and 36.03 thousand (0.53 individuals per 100,000 population) lost years of healthy life due to IDD attributable to CHD; (2) socioeconomic levels profoundly impact the burden of IDD attributable to CHD, with the higher prevalence and DALYs in socioeconomic disadvantaged regions except low SDI region; (3) geographical differences of IDD attributable to CHD remained a concern, with South Asia presenting the heaviest burden; (4) children under the age of five constituted the predominant population affected by IDD attributable to CHD, with the most significant numbers in preschoolers and rates in neonates; (5) the temporal trends of IDD attributable to CHD varied across different SDI regions and age-specific subgroups, with an overall decrease in rates but increase in number in low SDI regions and adult population; and (6) health-related economic investment for IDD attributable to CHD varied across countries and territories, and the IQ loss among CHD population may substantially hinder their future economic growth.

Consistent with previous studies (6,7,8,20,21), our findings demonstrated the susceptibility of IDD in CHD population. Based on the available data of GBD study 2021, approximately 6.6% CHD patients were comorbid with IDD in 2021 worldwide, significantly higher than the global prevalence of IDD of 2.35% in the general population during the same period. However, the underlying mechanisms of comorbidity between CHD and IDD have not been fully elucidated. Intrinsic and external factors contribute to the neurodevelopment impairment in the CHD population (6,10,22,23,24,25,26,27,28). First, abnormal hemodynamics primarily impact the IDD of complex CHD patients. Limperopoulos et al. found that anoxia in significant brain regions and neurodevelopment relied metabolic substrate were associated with the reduction of brain volumes, impediment of neuroaxonal development, and dysregulation of neurological metabolism in CHD fetuses with disturbed hemodynamics (25). During the infancy period, reduced cerebral blood flow and oxygenation due to cardiac defects also procrastinated the maturity and development of the cortex which further contributed to the cognitive impairments (26). Second, the advanced life support techniques, such as deep hypothermic circulatory arrest and extracorporeal membrane oxygenation support, were also linked to abnormal changes in brain metabolism among term-born CHD infants (27). Third, dysregulated coagulation caused by prolonged cardiopulmonary bypass and inappropriate warfarin administration in CHD patients who received mechanical prostheses, significantly increased the risk of stroke, resulting in motor, speech, and even cognitive functional impairments (28). Fourth, neonates underwent cardiac surgeries were inevitably exposed to excessive antibiotics in early life, which may be involved in the pathological process of neurodevelopment by reestablishing the gut macrobiotic environment (29).

Socioeconomic inequality remains a crucial factor influencing the burden of IDD attributable to CHD. Previous studies have reported that socioeconomic disadvantage was a fundamental risk factor of neurodevelopmental disability among the CHD population (6,30,31,32). Our findings revealed that the burden of IDD attributable to CHD was most pronounced in low-middle SDI regions, rather than in low SDI regions. Johnson et al. summarized that child poverty reshaped their developing brain through the intricate interactions between genetic and environmental influences, including neural plasticity, epigenetics, constrained cognitive stimulation, inadequate nutritional supplementation, family stressors, and exposure to environmental toxins (33). Socioeconomically, the limited accessibility of medical resources and high costs along with the limited commercial insurance in poverty-stricken regions exacerbated the health outcomes and disease burden related to neurodevelopmental disabilities for CHD patients (34). However, previous data indicated the mortality of CHD was higher in low SDI regions than other regions due to the inherent impact of socioeconomic disadvantages (35). Epidemiological investigations of non-fatal comorbidities as IDD could only be conducted among survivors that healthy life years loss is difficult to evaluate among deceased CHD population, which may underestimate the burden of IDD attributable to CHD in low SDI regions. Collectively, hygienic politics should prioritize reducing CHD mortality in low-SDI regions, whereas gradually allocating increased resources toward IDD in low-middle SDI regions to mitigate the burden of neurodevelopment of CHD population.

Geographically, South Asia was the region profoundly affected by both CHD and IDD (35,36,37,38), and our findings confirm that it continues to bear the heaviest burden of IDD attributable to CHD. As exemplified by India, the largest country in the South Asia, exhibited the highest prevalence and DALYs among all 204 countries and territories in 2021. Pediatric patients with CHD in India experienced a long-term cyanosis, pulmonary hypertension, and end-stage heart failure, partly because of the religious culture, distant cardiac centers, delayed diagnosis, expensive private healthcare, and insufficient governmental funding (39). All these challenges may contribute to a greater burden by increasing hypoxic period, narrowing optimal surgical windows, and exacerbating neurological impairment. Additionally, Australia and New Zealand, despite being high-income countries, showed the highest annual increase in IDD attributable to CHD from 1990 to 2021. Even though progress of congenital cardiac surgery significantly reduced the CHD mortality in Australians, recent data also indicated a gradual rise in developmental concerns, as reflected in a national census report (40). The disparity in resource allocation between addressing disability and reducing mortality was lagging compared to the increasing demand for non-fatal disability of primary causes (41), potentially aggregating the burden of IDD attributable to CHD observed in Australians.

The most critical age period affected by IDD attributable to CHD has not been previously reported using global data, which is essential for guiding hygienic resources allocation. Our study observed a heavier burden of IDD attributable to CHD in the first 5 years of life, particularly in infancy. This disproportionate burden may be partly attributable to the improved survival among children with CHD (2). In addition, the proportion of complex CHD is typically diagnosed in children younger than 5 years, and these lesions are prone to severe and multifaceted IDD, further amplifying the early-life burden (42). Even so, children with simple CHD are not free from risk and may still experience milder yet clinically relevant neurodevelopmental impairment. Together, these findings further support the notion that the period before five represents the crucial time window for neurodevelopment (43). Cerebrovascular accidents in early life are associated with more severe neurological impairments, contributing to a lifelong burden for individuals, families, and societies (44). Thus, delaying unnecessary surgeries in early life may give time for neurological maturation and reduce the lifelong burden of IDD attributable to CHD. Apart from surgical timing, early identification and proactive rehabilitation intervention of neurodevelopment among CHD patients under the age of five were also recommended by the American Heart Association (6).

Over the past three decades, the population number of IDD attributable to CHD drastically increased in low SDI regions, yet gradually decreased in high SDI regions, which aligned with the backdrop of high fertility in socioeconomically disadvantaged regions and population shrinking in developed countries during the same periods, respectively (45,46). Differently, the rate of IDD attributable to CHD decreased across all SDI regions, with the most significant decline observed in high SDI regions, likely driven by improvements in neuroprotective strategies in congenital cardiac surgery (47). In 2012, the American Heart Association released the statement of evaluation and management of neurodevelopmental outcomes in children with CHD (48). With growing medical facilities incorporating the statement into clinical practice, children under the age of five may benefit, and the impact may extend to older age subgroups over time. Although the number of adults with IDD attributable to CHD continued to rise, it remained far less than children under the age of five. Overall, investments of emerging medical resources to improve IDD attributable to CHD should be prioritized for CHD children under the age of five in socioeconomical disadvantaged regions, as they represent the dominant group for neurodevelopmental disability in the future.

Cost analysis revealed a significant disparity between economic investment and the disease burden of IDD attributable to CHD across 204 countries and territories. For example, India had the highest prevalence of IDD attributable to CHD cases in 2021, yet its direct costs represented merely one-twentieth of that in the United States. This disproportion primarily reflects variations in health expenditure per capita across regions, as demonstrated by our cost model. Moreover, our findings indicate that regions with higher numbers of IDD attributable to CHD face substantially greater indirect costs, suggesting an accumulating long-term economic burden in the future. Taken together, cost analyses underscore the unmet requirements for health investments that are commensurate with the burden of IDD attributable to CHD. Importantly, early identification and intervention to prevent IQ loss in CHD population may mitigate future national economic loss.

This study has several limitations. First, the insufficient availability of epidemiological data and unavoidable biases of accessible primary data may affect the estimation of the presented prevalence and DALY (13). Second, the burden of IDD attributable to specific subtypes of CHD was not further analyzed due to the unavailability of data in GBD 2021. Third, there was no available data of physical, psychological, and other aspects of developmental disability in GBD 2021, which hindered a multidimensional analysis. Fourth, the estimation of direct costs may be conservative, as it only limitedly accounted for variations in healthcare prioritization of IDD attributable to CHD across countries and territories. Given the short of concerning about the neurodevelopment in CHD population, the direct costs in our cost model may be overestimated.

## Conclusion

Globally, a total of 1.05 million people lived with IDD attributable to CHD, and 36.03 thousand individuals lost their years of health life due to IDD attributable to CHD in 2021. Socioeconomic and regional inequality remain a crucial issue. Pediatric CHD patients under the age of 5 years are the majority affected by IDD attributable to CHD. Medical resources for both reducing CHD mortality and improving neurodevelopmental outcomes should be coordinately allocated.

## Article Summary

These findings highlight the importance of coordinated allocation of public health resource to reduce mortality and improve neurodevelopmental outcomes in children with congenital heart disease.

### What is already known on this topic

In recent years, survival rates among patients with congenital heart disease have improved. As a result, neurodevelopmental disability has emerged as an important non-fatal complication in this growing survivor population.

### What this study adds

This study estimated that, in 2021, approximately 1.05 million individuals were living with intellectual developmental disability attributable to congenital heart disease worldwide. Substantial disparities in burden and trends were observed across regions and age groups. Health-related economic investment was limited and is projected to impose a significant indirect loss in the future.

## Additional File

The additional file for this article can be found as follows:

10.5334/gh.1511.s1 Supplementary Appendix. eAppendix 1 to 6.

## Abbreviations

CHD, Congenital heart disease; IDD, Intellectual developmental disability; GBD, Global burden of disease; YLL, Years of life lost; YLD, Years lived with disabilities; DALYs, Disability-adjusted life years; SDI, Social-demographic index; EAPC, Estimated annual percentage changes; ASR, Age-standardized rate; APC, Annual percent change; AAPC, Average annual percent change; UI, Uncertainty interval; CI, Confidence interval; IQ, Intelligence quotient.
