# Supplementary material for: Disease and Economic Burden of Intellectual Developmental Disability Attributable to Congenital Heart Disease, 1990–2021
Source: Glob Heart. 2026 Jan 16;21(1):2. doi: 10.5334/gh.1511 (PMC12829444; doi:10.5334/gh.1511)
Supplement: Supplementary Appendix. — eAppendix 1 to 6. [file gh-21-1-1511-s1.pdf]

Supplementary Appendix

Content

eAppendix 1. Definition description

eAppendix 2. Molding details

eAppendix 3. Global burden of IDD attributable to CHD across 204 countries and territories

eAppendix 4. Global burden of IDD attributable to CHD across age subgroups

eAppendix 5. Temporal trends of DID attributable to CHD from 1990 to 2021

eAppendix 6. The cost of IDD attributable to CHD in 2021

eAppendix 1. Definition description

DALYs

The disability adjusted life years (DALYs), one of the most significant indicators to measure the burden of intellectual developmental disability (IDD) attributable to congenital heart disease (CHD), are calculated by the sum of years of lost life (YLL) and years lived with disability (YLD) [1]. This study utilizes the YLD to represent the DALY due to the non-fatal nature of IDD as previous reported [2].

IDD

In the global burden of disease (GBD) study 2021, IDD was defined as a condition of significantly limited intellectual functioning and adaptive behavior originated before the age of eighteen, which was consistent with the definition of American Association on Intellectual and Developmental Disabilities [1]. Moreover, the etiology-specific IDD was estimated using mutually exclusive causes. As a result, the burden of IDD attributable to CHD estimated in this study was excluded from other confounding factors (e.g., the preterm birth and genetic syndrome) [1]. In the GBD study 2021, the IDD was commonly measured with intelligence quotient (IQ) tests, including Wechsler Preschool and Primary Scale of Intelligence, Wechsler Intelligence Scale for Children, Wechsler Adult Intelligence Scale, and etc. Thus, the hierarchical levels of IDD was classified based on IQ scores: borderline (IQ score 70–85), mild (IQ score 50–69), moderate (IQ score 35–49), severe (IQ score 20–34), and profound (IQ score 0–19) intellectual disability [1].

CHD

The CHD was defined as a spectrum of congenital circulatory malformation and categorized based on anatomical characteristics and the treatment requirements of each condition: i) single ventricle and single ventricle pathway defects; ii) complex congenital heart defects excluding single ventricle and single ventricle pathway defects; iii) malformations of great vessels, congenital valvular heart disease, and patent ductus arteriosus; iv) ventricular septal defect and atrial septal defect; and v) other congenital cardiovascular anomalies [1].

SDI

The social-demographic index (SDI) was evaluated to scale the socioeconomic background of certain locations and was measured by lag-distributed income per capita, average years of schooling, and the fertility rate in females under the age of 25 years [1]. All locations in certain years were grouped into five SDI regions based on the calculated SDI, ranging from low SDI, low-meddle SDI, middle SDI, high-middle SDI, and high SDI [1].

Age

A total of 25 original age subgroups were categorized in the GBD study 2021 [1]. Therein, eight typical age subgroups were selected of our study based on the integrated child-life

stages for Eunice Kennedy Shriver National Institute of Child Health and Human Development pediatric terminology [3] and the Cardiac Neurodevelopmental Outcome Collaborative [4]. Specifically, they were neonates (i.e., aged 0 to 28 days), early infants (i.e., aged 1 to 5 months), late infants (i.e., aged 6 to 11 months), toddlers (i.e., aged 12 to 23 months), preschoolers (i.e., aged 2 to 4 years), schoolers (i.e., aged 5 to 9 years), adolescents (i.e., aged 10 to 19 years), and adults (i.e., aged over 20 years).

eAppendix 2. Molding details

EAPC

The estimated annual percentage change (EAPC), first proposed by Hankey, is an indicator to measure the yearly temporal trends of age-standardized prevalence and DALY over a time interval [5]. In this study, the EAPC was calculated with the following formulas:

$$ln(ASR) = \alpha + \beta x + \varepsilon \#(1)$$

$$EAPC = 100 \times (exp(\beta) - 1)\#(2)$$

Age-standardized rate could be fitted in a regression model, where the α is the intercept term, β is the annual change per 100,000 in the rates, x is the calendar year, and ε is the error term. And the EAPC is calculated with its 95% confidence interval (95%CI) computed similarly from the linear regression model [5].

APC and AAPC

The annual percentage change (APC) represents the average annual percentage change of the dependent variable, to evaluate the internal trend of each independent interval of a segmented function or the overall trend with no connecting points [6]. For example, in a logarithmic linear model:

$$ln(y) = \beta_0 + \beta_1 x\#(3)$$

where y represents the prevalence or DALY rate and x represents the year, the formula for calculating APC in the fitted model can be derived as:

$$APC = \left[\frac{y_{x+1} - y_x}{y_x}\right] \times 100 = \left(e^{\beta_1} - 1\right) \times 100\#(4)$$

when assessing the overall average change trend encompassing multiple intervals, the average annual percentage change (AAPC) was utilized [6], with the following formula:

$$AAPC = \left(e^{\sum w_i \beta_i} - 1\right) \times 100\#(5)$$

In the above formula, w_i represents the width of each segment function interval (i.e., the number of years included in the interval), β_i denoted the regression coefficient corresponding to each interval.

Cost of the IDD attributable to CHD

The cost of IDD attributable to CHD in 2021 is modeled as direct cost and indirect cost in our study design. Direct cost refers to healthcare-associated expenditures of IDD in 2021, including financial inputs from families, societies, government health systems, etc. Indirect cost is estimated as the present value of loss in lifelong income from IQ loss. In the GBD 2021, the borderline IDD refers to individuals with slow learning or difficulty with complex tasks, which has significant lagging in health expenditure and impact on lifelong income compared to other levels of IDD [1]. Thus, the borderline IDD is excluded from the cost model to mitigate potential overestimation of economic burden. All the cost estimations were

performed with sensitivity analysis to account for underlying uncertainty by adjusting the lower and upper data of patients number of IDD attributable to CHD in the GBD 2021.

$$tC = dC + iC \#(6)$$

where the *tC* is the total cost, the *dC* is the direct cost, and the *iC* is the indirect cost.

Direct cost

Prior studies on the economic cost of IDD have primarily focused on broader IDD population, rather than specifically estimating the cost of IDD among CHD population [7, 8]. This crude scope limits the use of macroeconomic data necessary to quantify the direct healthcare-related costs of IDD attributable to CHD at the population level. Medicaid Home and Community Based Services (HCBS) waivers represent the benchmark of cost-effective funding mechanism for the long-term survives and supports of patients lived with IDD. In 2021, an average of $47,315 was expended per person with IDD for HCBS, including residential habilitation, inhabitancy, day habilitation, health and professional services, supported employment, and etc. [8]. Based on this, we set the direct cost of IDD attributable to CHD as $47,315 (US dollar in 2021) per person in the United States (US) in 2021 [8].

The direct cost of IDD attributable to CHD in the US are calculated by multiplying the reference cost of $47,315 per person by its number of IDD attributable to CHD in 2021. To extrapolate other national level cost, we assuming that per case direct cost for IDD was proportional to the health expenditure per capita (PHE) of the country, as previous studies have postulated [9-11]. Accordingly, we adjust these national-level cost using the ratio of national health expenditure per capita in 2021 with the following formula:

$$dC_i = N_i \times \mathrm{C_{ref}} \times \frac{PHE_i}{\mathrm{PHE_{ref}}} \#(7)$$

where dC_i is the national direct cost of IDD, N_i is the patients number of IDD attributable to CHD, $\mathrm{C_{ref}}$ is the reference cost of IDD in the US in 2021, the $\mathrm{PHE_{ref}}$ is the health expenditure per capita in the US in 2021, and the PHE_i is the health expenditure per capita of other countries in 2021.

For example, in India, with a patient number of 224622.78, the direct cost of reference is $47,315, the PHE of India was 75.54971, and the PHE of the US was 11999.0908. The direct cost of IDD attributable to CHD was estimated at $66.92 million in 2021.

Indirect cost

The indirect cost of IDD attributable to CHD is defined as the present value of lifetime income loss from IQ loss in CHD populations. In much-cited studies, the relationship between lifelong income loss and IQ loss has been established in the population of lead exposure[12-15]. These studies assumed that a higher IQ has a straightforward impact on the income and increases the income through higher educational levels and labor-force

participations [12, 13, 15]. Although the underlying mechanisms of IQ loss between lead exposure and CHD differ, their collective outcome of IQ loss may have the similar manner to refine lifelong income. Aligned with prior estimations, a lifelong income effect of 2.0% per IQ point is set in our study [15].

$$iC_i = \alpha \times \Delta IQ_i \times PV_0(I)\#(8)$$

where α is the lifelong income effect per IQ point (2.0% per IQ point), ΔIQ_i is the IQ loss at a national level in 2021 due to IDD attributable to CHD, $PV_0(I)$ is the present value of lifetime income in 2021.

For example, in India, with an estimated ΔIQ of 2498942 points based on formula No.9, an average GDP per employed person of $22095.3, and an expected income growth rate of 2.5%, the indirect cost of IDD attributable to CHD was estimated at $22,148.47 million in 2021.

IQ loss

The estimation of IQ loss was calculated based on the difference between the median IQ values at hierarchical levels of IDD and the IQ of 70, which is the lower limit of borderline IDD and associated with significant impairments of conceptual and practical function [16]. Employing this reference value helps mitigate the risk of overestimation the cost of IQ loss. The detailed formula is presented as:

$$\Delta IQ_i = \sum_{s=1}^{S} N_s \times (IQ_{ref} - IQ_s)\#(9)$$

where the ΔIQ_i is the total IQ loss at the population level, s is the certain levels of IDD attributable to CHD (i.e., borderline, mild, moderate, severe, and profound), N_s is the number of IDD attributable to CHD at the corresponding IDD levels, IQ_{ref} is set to 70, and the IQ_s is set according to the STable 1.

STable 1. IQ values at different hierarchical levels of IDD attributable to CHD

IDD levels	IQ scores	IQ_s	IQ_{ref}	ΔIQ_s
Mild	50 ~ 69	59.5	70	10.5
Moderate	35 ~ 49	42	70	28
Severe	20 ~ 34	27	70	43
Profound	0 ~ 19	9.5	70	50[#]

[#] ΔIQ_s of profound IDD is adjusted as 50 due to maximal lifelong income loss of 100% at the lifetime income effect of 2% per IQ loss.

Present value

We calculate the present value of lifetime income in 2021 with:

$$PV_0(I) = \sum_{i=k}^{i=n} I_0(1+g)^i/(1+r)^i\,\#(10)$$

where I_0 is the average annual income in the country in 2021, g is the rate of future income growth, r is the discounting rate of future income, k is the age of workforce entry minus 3 years, and n is the age of retirement less 3 years.

The average annual income in a country is set as the gross domestic product (GDP) per person employed in 2021 from the World Data Bank [17]. The future income growth rate is set to recent historical GDP per capita growth in high-income countries; slightly lower in middle-income countries due to economic maturation, and relatively higher in low-income countries to reflect expected catch-up growth [15]. In accordance with World Bank recommendation, the discount rate for future income is assumed to be twice the projected income growth rate in each country [18]. The age of entering and retirement is referred from Larsen and Sánchez-Triana [15], and the onset age of IQ loss is set at preschoolers of 3 yeas based on the median age of population distribution within this study. Parameters utilized to calculate the present value of lifetime income in 2021 were shown in the STable 2.

STable 2. Parameter values to calculate the present value of lifetime income

Parameter	Meaning	Low-income countries[#]	Middle-income countries[#]	High-income countries[#]
I	Average annual income	GDP per person employed in 2021 from Data Bank		
g	Future income growth	2.5%	2.5%	1.5%
r	Discounting rate of future income	5.0%	5.0%	3.0%
k	Age of entering the workforce	15 years	18 years	21 years
n	Age of retirement	60 years	65 years	65 years

[#]The economic classification of countries worldwide in 2021 is assigned by Work bank, based on the gross national income (GNI) per capita in current US dollar (Atlas method) [19]. Low-income ($1045), middle-income ($1046-$12,695), and high-income (> $12,695).

Reference

1. Ferrari, A.J., et al., *Global incidence, prevalence, years lived with disability (YLDs), disability-adjusted life-years (DALYs), and healthy life expectancy (HALE) for 371 diseases and injuries in 204 countries and territories and 811 subnational locations, 1990–2021: a systematic analysis for the Global Burden of Disease Study 2021.* The Lancet, 2024. **403**(10440): p. 2133-2161.
2. Steinmetz, J.D., et al., *Global, regional, and national burden of disorders affecting the nervous system, 1990–2021: a systematic analysis for the Global Burden of Disease Study 2021.* The Lancet Neurology, 2024. **23**(4): p. 344-381.
3. Cohen Hubal, E.A., et al., *Identifying important life stages for monitoring and assessing risks from exposures to environmental contaminants: results of a World Health Organization review.* Regul Toxicol Pharmacol, 2014. **69**(1): p. 113-24.
4. Ware, J., et al., *Neurodevelopmental evaluation strategies for children with congenital heart disease aged birth through 5 years: recommendations from the cardiac*

neurodevelopmental outcome collaborative. Cardiol Young, 2020. **30**(11): p. 1609-1622.

5. Hankey, B.F., et al., *Partitioning linear trends in age-adjusted rates.* Cancer Causes Control, 2000. **11**(1): p. 31-5.
6. Clegg, L.X., et al., *Estimating average annual per cent change in trend analysis.* Stat Med, 2009. **28**(29): p. 3670-82.
7. Genereaux, D., C.D. van Karnebeek, and P.H. Birch, *Costs of caring for children with an intellectual developmental disorder.* Disabil Health J, 2015. **8**(4): p. 646-51.
8. Friedman, C., *Medicaid Home- and Community-Based Services Waivers for People With Intellectual and Developmental Disabilities.* Intellect Dev Disabil, 2023. **61**(4): p. 269-279.
9. Ding, D., et al., *The economic burden of physical inactivity: a global analysis of major non-communicable diseases.* Lancet, 2016. **388**(10051): p. 1311-24.
10. Chen, S., et al., *The global macroeconomic burden of road injuries: estimates and projections for 166 countries.* Lancet Planet Health, 2019. **3**(9): p. e390-e398.
11. Chen, S., et al., *Estimates and Projections of the Global Economic Cost of 29 Cancers in 204 Countries and Territories From 2020 to 2050.* JAMA Oncol, 2023. **9**(4): p. 465-472.
12. Schwartz, J., *Societal benefits of reducing lead exposure.* Environ Res, 1994. **66**(1): p. 105-24.
13. Salkever, D.S., *Updated estimates of earnings benefits from reduced exposure of children to environmental lead.* Environ Res, 1995. **70**(1): p. 1-6.
14. Attina, T.M. and L. Trasande, *Economic costs of childhood lead exposure in low- and middle-income countries.* Environ Health Perspect, 2013. **121**(9): p. 1097-102.
15. Larsen, B. and E. Sánchez-Triana, *Global health burden and cost of lead exposure in children and adults: a health impact and economic modelling analysis.* Lancet Planet Health, 2023. **7**(10): p. e831-e840.
16. *Defining Criteria for Intellectual Disability.*; Available from: https://www.aaidd.org/intellectual-disability/definition.
17. *World Bank. World Development Indicators. Washington, DC 2021.https://databank.worldbank.org/source/world-development-indicators#.*
18. *Discounting Costs and Benefits in Economic Analysis of World Bank Projects (English). Washington, D.C. : World Bank Group. http://documents.worldbank.org/curated/en/099610503022315638.*
19. METREAU, N.H.V.R. *New World Bank country classifications by income level: 2021-2022*

(https://blogs.worldbank.org/en/opendata/new-world-bank-country-classifications-income-level-2021-2022). JULY 01, 2021.

eAppendix 3. Global burden of IDD attributable to CHD across 204 countries (STable 3 and STable 4)

STable 3. The prevalence in all-aged number and age-standardized rates and EAPC of IDD attributable to CHD across 204 countries and territories from 1990 to 2021

	1990		2021		1990-2021
	Number No.(95%UI)	ASPR per 100,000 No.(95%UI)	Number No.(95%UI)	ASPR per 100,000 No.(95%UI)	EAPC No.(95%CI)
**High-income**					
**High-income North America**					
Canada	3057.27 (996.12-3836.86)	15.51 (4.89-19.51)	2850.44 (1214.57-3660.38)	14.12 (5.63-18.2)	-0.52 (-0.59--0.44)
Greenland	6.69 (3.02-8.27)	12.08 (5.52-14.90)	4.57 (1.84-5.62)	11.18 (4.38-13.73)	-0.4 (-0.46--0.34)
United States of America	26415.84 (13502.36-32019.05)	13.07 (6.62-15.83)	24885.03 (10387.91-30184.88)	12.6 (5.05-15.33)	-0.09 (-0.13--0.05)
**Australasia**					
Australia	971.52 (35.72-1553.05)	7.53 (0.22-12.12)	1599.19 (559.06-2234.4)	10.08 (3.32-14.14)	0.93 (0.70-1.16)
New Zealand	257.34 (17.88-373.24)	9.06 (0.58-13.14)	378.64 (151.59-491.49)	11.54 (4.34-14.99)	0.89 (0.68-1.1)
**High-income Asia Pacific**					
Brunei Darussalam	18.22 (0.02-39.94)	5.44 (0.02-11.84)	8.75 (0-31.86)	2.72 (0-10)	-2.46 (-2.80--2.12)
Japan	8368.49 (1169.71-12124.91)	11.15 (1.15-16.08)	3179.78 (92.25-6744.72)	5.86 (0.12-12.61)	-1.81 (-1.95--1.67)
Singapore	199.71 (6.76-304.22)	9.39 (0.24-14.36)	47.21 (0-297.72)	1.53 (0-9.77)	-5.37 (-5.95--4.78)
Republic of Korea	5105.7 (1070.88-6702.41)	14.71 (2.79-19.37)	595.72 (0.04-2157.1)	3.18 (0-11.98)	-4.45 (-4.82--4.08)
**Western Europe**					
Andorra	3.65 (1.32-4.68)	11.63 (3.42-15.00)	3.84 (1.67-5.02)	11.28 (3.74-14.89)	-0.14 (-0.22--0.06)
Austria	789.26 (434.82-944.91)	15.46 (8.05-18.54)	787.23 (304.45-953.41)	15.58 (5.07-19.01)	-0.18 (-0.28--0.07)
Belgium	1085.67 (648.67-1287.37)	15.96 (8.84-19.11)	927.69 (439.46-1158.51)	13.69 (5.76-17.02)	-0.49 (-0.65--0.32)
Cyprus	100.16 (58.48-122.92)	15.05 (8.64-18.43)	116.49 (61.63-146.58)	13.76 (6.78-17.12)	-0.31 (-0.38--0.24)
Denmark	216.83 (22.03-444.1)	6.62 (0.39-13.97)	172.52 (11.66-318.28)	4.84 (0.17-9.26)	-1.16 (-1.22--1.11)
Finland	671.92 (385.68-824.98)	19.56 (10.52-24.32)	517.59 (263.55-652.65)	18.27 (8.33-23.12)	-0.33 (-0.47--0.18)

France	9238.01 (5539.19-11567.73)	22.15 (12.69-27.77)	7741.35 (4523.04-9815.09)	19.53 (10.67-25.15)	-0.29 (-0.34--0.25)
Germany	7035.58 (2549.42-8763.29)	14.28 (4.63-17.88)	5678.9 (1131.56-7713.13)	12.48 (1.96-17.04)	-0.36 (-0.44--0.27)
Greece	1044.41 (646.69-1261.84)	15.92 (9.1-19.33)	676.34 (360.83-817.76)	13.42 (6.4-16.22)	-0.59 (-0.65--0.53)
Iceland	25.26 (10.39-31.59)	11.5 (4.66-14.38)	25.25 (8.05-32.78)	10.56 (3.05-13.7)	-0.34 (-0.38--0.31)
Ireland	423.63 (236.22-518.15)	13.95 (7.57-16.99)	364.47 (103.86-493.47)	11.18(2.8-15.26)	-0.87 (-1.03--0.72)
Israel	696.8 (411.03-857.66)	13.64 (8.07-16.76)	1226.43 (621.05-1508.99)	13.33 (6.73-16.4)	-0.11 (-0.15--0.08)
Italy	4876.64 (2398.59-5963.01)	15.13 (6.73-18.56)	3871.63 (1903.42-4784.32)	14.01 (5.85-17.23)	-0.17 (-0.46-0.11)
Luxembourg	33.57 (16.33-42.58)	13.19 (6-16.91)	43.31 (15.05-58.22)	11.62 (3.46-15.64)	-0.37 (-0.44--0.3)
Malta	42.85 (27.82-51.77)	14.32 (9.01-17.33)	31.59 (13.94-39.14)	12.7 (4.92-15.98)	-0.24 (-0.28--0.19)
Monaco	1.17 (0.17-1.87)	8.13 (0.71-13.34)	1.3 (0.13-2.38)	6.86 (0.36-13.03)	-0.53 (-0.54—0.52)
Netherlands	1375.27 (589.87-1711.66)	13.29 (5.23-16.59)	1019.98 (300.89-1352.41)	10.36 (2.57-13.88)	-0.79 (-0.88--0.71)
Norway	399.69 (200.29-492.4)	13.23 (6.32-16.28)	410.8 (173.87-523.36)	12.77 (4.87-16.27)	-0.38 (-0.47--0.29)
Portugal	926.32 (630.61-1130.03)	14.14 (8.97-17.51)	676.07 (376.89-839.26)	13.4 (6.9-16.74)	-0.12 (-0.16--0.08)
San Marino	1.59 (0.75-2.07)	11.35 (4.78-14.55)	1.76 (0.82-2.26)	11.54 (4.38-14.74)	0.04 (-0.11-0.2)
Spain	4954.16 (4248.03-5724.9)	19.85 (17-23.19)	4739.47 (3941.8-5435.54)	19.85 (16.57-23.08)	-0.3 (-0.39--0.2)
Sweden	989.06 (477.99-1297.79)	16.89 (7.77-22.31)	906.33 (433.29-1174.87)	14.42 (6.61-18.75)	-0.67 (-0.97--0.37)
Switzerland	649.32 (262.71-788.22)	14.59 (5.31-17.89)	638.32 (235.99-821.09)	12.58 (4.02-16.28)	-0.53 (-0.57--0.5)
United Kingdom	5056.57 (3736.59-5926.35)	11.98 (8.52-14.1)	5176.35 (2950.09-6229.02)	12.58 (6.78-15.08)	0.26 (0.2-0.32)
**Southern Latin America**					
Argentina	4020.48 (2523.26-4946.6)	11.81 (7.4-14.54)	3678.93 (1844.97-4624.16)	11.94 (5.73-15.01)	-0.05 (-0.11-0)
Chile	2096.38 (1510.05-2564.31)	14.7 (10.6-17.96)	1545.55 (523.53-1884.59)	13.54 (4.26-16.57)	-0.34 (-0.4--0.27)
Uruguay	369.47 (271.95-453.57)	13.39 (9.84-16.47)	270 (116.54-341.5)	13.26 (5.43-16.77)	0 (-0.03-0.04)
**Central Europe, eastern Europe, and central Asia**					
**Eastern Europe**					

Belarus	1610.53 (946.15-1982.74)	19.51 (11.25-24.01)	924.66 (455.08-1154.88)	18.13 (8.25-22.63)	-0.34 (-0.4--0.28)
Estonia	218.1 (123.43-270)	17.73 (9.85-21.98)	114.39 (31.5-147.91)	15.6 (3.87-19.99)	-0.61 (-0.7--0.52)
Latvia	384.04 (192.31-478.9)	18.53 (9.14-23.13)	167.27 (55.4-212.84)	16.58 (4.86-21.18)	-0.44 (-0.49--0.39)
Lithuania	547.39 (290.41-680.99)	18.56 (9.7-23.16)	230.95 (68.76-293.42)	16.36 (4.19-20.97)	-0.55 (-0.6--0.5)
Republic of Moldova	820.05 (647.81-997.83)	19.18 (15.12-23.36)	333.37 (272.69-399.05)	19.2 (15.4-23.15)	-0.05 (-0.1-0)
Russian Federation	18991.59 (8423.52-23456.61)	16.06 (6.86-19.95)	12726.59 (5370.18-15804.33)	15.65 (6.05-19.57)	-0.25 (-0.33--0.16)
Ukraine	7716.73 (4881.55-9347.1)	19.81 (12.28-24.05)	3851.98 (2842.14-4592.28)	20.67 (14.59-24.89)	0.08 (0.02-0.13)
**Central Europe**					
Albania	673.67 (537.95-814.55)	17.08 (13.65-20.57)	249.88 (155.32-305.74)	16.43 (9.8-20.26)	-0.18 (-0.2--0.15)
Bosnia and Herzegovina	715.43 (599.28-844.2)	19.77 (16.58-23.35)	286.92 (162.94-353.38)	17.36 (9.19-21.38)	-0.49 (-0.52--0.46)
Bulgaria	1126.34 (700.08-1374.46)	20.07 (12.11-24.5)	620.51 (254.27-777.31)	19.02 (7.04-23.86)	-0.01 (-0.1-0.09)
Croatia	493.91 (201.96-614.86)	15.52 (6.05-19.43)	294.95 (86.02-377.19)	14.84 (3.66-19.01)	-0.1 (-0.19--0.02)
Czechia	1162.71 (351.24-1495.04)	17.11 (4.75-22.21)	910.7 (202.66-1200.47)	15.47 (3.09-20.5)	-0.51 (-0.63--0.4)
Hungary	1106.74 (421.07-1400.68)	16.92 (6.03-21.47)	738.21 (185.61-947.61)	15.26 (3.4-19.55)	-0.53 (-0.61--0.45)
North Macedonia	280.18 (199.51-340.75)	16.21 (11.41-19.73)	167.86 (84.67-210.68)	15.23 (7.07-19.15)	-0.29 (-0.33--0.25)
Montenegro	72.43 (37.06-90.12)	13.6 (6.78-16.96)	51.08 (20.29-63.74)	13.39 (4.93-16.79)	-0.24 (-0.33--0.14)
Poland	5898.6 (4141.91-7102.93)	19.62 (13.59-23.61)	3570.31 (1200.04-4416.79)	18.03 (5.65-22.31)	-0.49 (-0.61--0.37)
Romania	3024.9 (2150.27-3716.22)	16.88 (11.75-20.8)	1511.87 (497.9-1894.35)	15.3 (4.66-19.18)	-0.42 (-0.47--0.37)
Serbia	1105.19 (619.65-1366.66)	15.62 (8.52-19.43)	630.91 (321.7-785.73)	15.39 (7.24-19.22)	-0.16 (-0.22--0.09)
Slovakia	687.72 (302.97-850.1)	16.44 (7.11-20.34)	445.44 (116.69-569.31)	14.85 (3.52-19.04)	-0.39 (-0.41--0.37)
Slovenia	205.92 (68.72-262.45)	15.86 (4.87-20.27)	154.14 (36.87-200.34)	14.7 (3.02-19.2)	-0.38 (-0.43--0.32)
**Central Asia**					
Armenia	932.37 (725.17-1142.82)	24.95 (19.46-30.52)	502.09 (336.93-613.73)	25.18 (16.4-30.77)	-0.01 (-0.05-0.03)
Azerbaijan	1958.47	22.33	1605.11	21.44	-0.32

	(1386.57-2471.62)	(15.8-28.14)	(859.23-2028.13)	(10.78-27.19)	(-0.41--0.22)
Georgia	896.52 (678.04-1065.74)	18.89 (14.19-22.52)	519.86 (379.81-620.55)	20.4 (14.76-24.33)	0.29 (0.19-0.4)
Kazakhstan	4090.34 (2878.06-5137.07)	22.24 (15.66-27.92)	4063.91 (1566.77-5167.95)	20.97 (8.16-26.63)	-0.33 (-0.4--0.26)
Kyrgyzstan	1419.39 (1126.93-1770.51)	23.18 (18.42-28.72)	1886.58 (1512.88-2302.43)	24.38 (19.58-29.69)	0.1 (0.05-0.16)
Mongolia	893.47 (695.63-1115.77)	27.44 (21.42-34.11)	947.83 (644.33-1173.2)	24.91 (16.92-30.79)	-0.47 (-0.52--0.42)
Tajikistan	2191.44 (1697.53-2761.86)	24.27 (18.85-30.51)	3373.06 (2656.41-4198.73)	25.87 (20.47-32.13)	0.02 (-0.09-0.14)
Turkmenistan	1291.7 (950.04-1633.7)	23.17 (16.99-28.97)	1204.64 (546.92-1512.06)	22.51 (10.23-28.25)	-0.2 (-0.24--0.16)
Uzbekistan	7656.77 (6036.55-9661.73)	23.94 (19.02-29.88)	9317.34 (6832.09-11519)	24.68 (18.18-30.46)	-0.05 (-0.1-0)
**Latin America and Caribbean**					
**Central Latin America**					
Colombia	5667.38 (3691.5-6940.94)	13.94 (9.18-17.07)	4552.33 (1905.75-5801.9)	12.63 (5.05-16.19)	-0.4 (-0.44--0.37)
Costa Rica	560.69 (273.7-706.9)	14.36 (7.14-18.11)	456.87 (170.41-598.17)	13.8 (4.62-18.19)	-0.14 (-0.17--0.12)
El Salvador	1088.47 (871.44-1341.13)	14.77 (11.71-18.11)	836.41 (568.67-1047.22)	13.81 (9.27-17.27)	-0.2 (-0.22--0.18)
Guatemala	1831 (1423.16-2264.62)	12.65 (9.74-15.65)	1940 (1452.73-2385.04)	12.48 (9.29-15.35)	0 (-0.02-0.02)
Honduras	1139.76 (901.24-1379.84)	14.71 (11.54-17.8)	1556.31 (1187.27-1884.34)	14.34 (10.92-17.33)	-0.1 (-0.11--0.08)
Mexico	15867.24 (6954.52-18980.99)	14.04 (6.41-16.79)	14429.7 (5741.27-17543.83)	13.97 (5.31-16.94)	-0.04 (-0.06--0.02)
Nicaragua	816.13 (632.89-1000.82)	13.17 (10.26-16.02)	862.74 (647.41-1024.67)	13.22 (9.94-15.71)	-0.02 (-0.05-0.02)
Panama	385.78 (214.67-475.39)	13.84 (7.85-16.97)	445.93 (115.2-592.34)	11.8 (2.94-15.68)	-0.51 (-0.55--0.47)
Venezuela (Bolivarian Republic of)	3053.51 (1311.99-3810.85)	12.55 (5.68-15.61)	3071.04 (1453.4-3865.06)	13.71 (6.31-17.28)	0.18 (0.1-0.26)
**Andean Latin America**					
Bolivia (Plurinational State of)	1342.04 (1101.97-1593.27)	14.13 (11.49-16.7)	1622.26 (1276.61-1932.91)	13.62 (10.7-16.22)	-0.15 (-0.17--0.14)
Ecuador	1776.6 (1353.72-2150.24)	13.73 (10.47-16.56)	2356.31 (1644.5-2858.06)	14.02 (9.68-17)	0.09 (0.03-0.15)
Peru	3733.29 (2905.31-4573.49)	13.26 (10.28-16.19)	4187.64 (2608.65-5131.39)	12.53 (7.7-15.35)	-0.23 (-0.24--0.22)
**Caribbean**					
Antigua and Barbuda	7.97 (3.19-10.13)	13.16 (5.23-16.73)	6.75 (2.26-8.64)	11.9 (3.62-15.3)	-0.31 (-0.34--0.29)

Bahamas	27.92 (6.72-35.98)	10.89 (2.55-14.06)	25.1 (7.87-32.22)	10.87 (2.88-14.03)	-0.09 (-0.13--0.06)
Barbados	27.01 (11.65-33.89)	13.33 (5.49-16.76)	20.8 (9.49-26.4)	13.54 (5.48-17.34)	0.07 (0.03-0.1)
Belize	34.3 (27.6-41.85)	12.32 (9.87-14.98)	45.11 (32.87-55.1)	11.59 (8.38-14.17)	-0.24 (-0.27--0.21)
Bermuda	5.29 (0.98-7.15)	11.9 (2.03-16.2)	2.85 (0.41-4.14)	9.87 (1.05-14.65)	-0.73 (-0.79--0.66)
Cuba	1405.63 (878.54-1781.05)	15.34 (9.37-19.54)	931.86 (631.18-1137.62)	15.03 (9.84-18.56)	-0.18 (-0.28--0.09)
Dominica	10.09 (7.8-12.41)	11.92 (9.23-14.71)	4.4 (3.33-5.32)	11.25 (7.86-13.63)	-0.23 (-0.25--0.21)
Dominican Republic	1087.68 (835.39-1327.5)	11.31 (8.66-13.83)	1003.81 (451.84-1241.08)	9.66 (4.31-11.92)	-0.61 (-0.64--0.57)
Grenada	15.83 (12.31-19.72)	13.78 (10.72-17.09)	9.19 (5.08-11.33)	12.68 (6.69-15.69)	-0.33 (-0.36--0.29)
Guyana	135.88 (107.71-162.11)	12.45 (9.91-14.91)	92.02 (70.83-114.36)	12.36 (9.52-15.33)	-0.02 (-0.06-0.02)
Haiti	1392.91 (1062.22-1752.89)	13.51 (10.22-17)	2188.37 (1771.66-2729.02)	14.16 (11.46-17.61)	0.15 (0.12-0.17)
Jamaica	342.1 (235.56-433.48)	12.52 (8.63-15.82)	235.59 (160.3-295.5)	12.77 (8.59-16.13)	0.02 (-0.02-0.06)
Puerto Rico	391.88 (111.89-510.99)	12.12 (3.37-15.85)	141.54 (32.1-185.57)	11.15 (1.97-15)	-0.42 (-0.53--0.31)
Saint Kitts and Nevis	5.49 (2.93-6.85)	11.92 (6.41-14.9)	3.47 (1.17-4.37)	10.32 (3.15-12.96)	-0.46 (-0.48--0.44)
Saint Lucia	20.61 (14.19-25.73)	12 (8.31-15.02)	11.05 (5.9-13.73)	11.37 (5.8-14.16)	-0.19 (-0.22--0.16)
Saint Vincent and the Grenadines	17.82 (13.87-21.93)	14.18 (11.05-17.38)	9.8 (6.62-12.3)	12.76 (8.42-16.03)	-0.38 (-0.41--0.34)
Suriname	49.54 (32.07-60.87)	11.47 (7.42-14.08)	51.02 (27.63-63.35)	11.09 (5.87-13.74)	-0.24 (-0.28--0.2)
Trinidad and Tobago	159.5 (74.25-203.15)	12.1 (5.6-15.33)	92.29 (16.65-119.62)	10.81 (1.76-14.16)	-0.53 (-0.59--0.47)
United States Virgin Islands	12.98 (3.98-16.7)	11.87 (3.65-15.27)	4.33 (0.77-5.77)	10.04 (1.51-13.39)	-0.64 (-0.79--0.48)
**Tropical Latin**					
Brazil	21787.23 (17015.44-25390.55)	13.48 (10.49-15.67)	21083.55 (14129.98-25120.78)	12.31 (8.11-14.69)	-0.3 (-0.36--0.24)
Paraguay	804.3 (618.37-985.94)	13.81 (10.61-16.83)	925.53 (630.46-1153.24)	14.02 (9.47-17.41)	0.08 (0.04-0.12)
**Southeast Asia, east Asia, and Oceania**					
**East Asia**					
China	170172.9 (130437.23-203651.52)	15.12 (11.57-18.11)	110805.52 (69658.01-133203.03)	13.28 (7.73-16)	-0.42 (-0.44--0.41)

Democratic People's Republic of Korea	3103.52 (2494.81-3725.9)	13.43 (10.8-16.09)	2437.71 (1989.64-2955.12)	14.54 (11.86-17.78)	0.24 (0.21-0.27)
Taiwan (Province of China)	1937.84 (738.74-2468.27)	11.53 (4.08-14.69)	793.69 (108.61-1142.86)	7.63 (0.72-11.1)	-1.32 (-1.38--1.27)
**Southeast Asia**					
Cambodia	3174.24 (2585.14-3840.02)	18.12 (14.84-21.84)	2823 (2274.97-3399.52)	16.23 (13.07-19.54)	-0.41 (-0.45--0.37)
Indonesia	36601.27 (30089.19-43860.19)	16.77 (13.8-20.08)	32463.9 (26891.27-38549.96)	14.34 (11.85-17.08)	-0.54 (-0.56--0.51)
Lao People's Democratic Republic	1321.25 (1063.53-1613.38)	19.17 (15.46-23.21)	1331.94 (1066.46-1632.81)	16.22 (13-19.85)	-0.59 (-0.63--0.56)
Malaysia	3140.17 (2489.66-3826.64)	13.71 (10.88-16.68)	3152.86 (1906.4-3945.54)	12.36 (7.36-15.43)	-0.35 (-0.36--0.34)
Maldives	53.86 (42.96-65.92)	13.84 (11.04-16.92)	40.56 (28.85-49.33)	11.79 (8.07-14.34)	-0.53 (-0.55--0.52)
Mauritius	156.45 (125.71-191.73)	14.65 (11.72-18.03)	84.04 (55.87-104.64)	11.78 (7.57-14.66)	-0.75 (-0.83--0.67)
Myanmar	9609.43 (7926.82-11848.01)	19.34 (15.99-23.76)	8323.71 (6693.64-10148.18)	15.83 (12.71-19.36)	-0.78 (-0.84--0.71)
Philippines	14196.56 (11795.53-16649.4)	15.93 (13.29-18.61)	16315.23 (13473.07-19167.74)	14.54 (12-17.08)	-0.28 (-0.3--0.26)
Sri Lanka	2491.17 (2001.29-3000.89)	14.09 (11.35-16.95)	2104.06 (1579.11-2573.94)	12.85 (9.67-15.78)	-0.26 (-0.28--0.24)
Seychelles	11.13 (8.7-13.67)	13.97 (10.92-17.14)	10.44 (6.6-12.8)	12.82 (8.02-15.7)	-0.22 (-0.24--0.19)
Thailand	8733.67 (7150.36-10442.11)	16.5 (13.48-19.71)	4704.28 (3909.22-5667.13)	14.31 (11.71-17.46)	-0.48 (-0.5--0.45)
Timor-Leste	223.88 (178.53-274.31)	16.39 (13.14-19.96)	270.07 (218.44-330.9)	14.87 (12.03-18.18)	-0.48 (-0.55--0.4)
Viet Nam	12966.85 (10657.3-15597.69)	14.41 (11.87-17.29)	10088.67 (6557.48-12336.8)	12.09 (7.73-14.77)	-0.59 (-0.63--0.55)
**Oceania**					
American Samoa	7.7 (3.69-9.58)	10.5 (5.23-13.02)	4.39 (3.28-5.44)	11.34 (8.34-13.94)	0.21 (0.17-0.25)
Cook Islands	2.53 (1.45-3.15)	11.54 (6.62-14.28)	1.22 (0.52-1.54)	10.16 (4.22-12.83)	-0.39 (-0.43--0.36)
Micronesia (Federated States of)	21.34 (17.01-25.97)	14.31 (11.44-17.33)	13.95 (11.53-16.99)	14.54 (12.02-17.76)	0.04 (0.01-0.07)
Fiji	123.55 (99.54-148.62)	13.46 (10.85-16.18)	117.64 (86.33-145.14)	12.94 (9.47-15.96)	-0.12 (-0.13--0.1)
Guam	15.37 (4.92-19.36)	9.63 (3.13-12.18)	11.93 (3.74-15.13)	9.14 (2.8-11.61)	-0.25 (-0.28--0.22)
Kiribati	17.81 (14.27-22.32)	15.59 (12.47-19.48)	22.84 (18.26-27.95)	16.29 (13.03-19.94)	0.15 (0.13-0.18)
Marshall Islands	10.07	14.31	8.21	14.45	0.04

	(8.1-12.11)	(11.45-17.2)	(6.7-9.98)	(11.82-17.55)	(0.02-0.06)
Nauru	1.96<br>(1.08-2.49)	12.42<br>(7.01-15.72)	1.79<br>(1.37-2.16)	13.07<br>(9.97-15.8)	0.17<br>(0.05-0.29)
Niue	0.32<br>(0.24-0.38)	12.65<br>(9.76-15.34)	0.15<br>(0.11-0.18)	11.84<br>(8.56-14.66)	-0.29<br>(-0.33--0.26)
Northern Mariana Islands	4.68<br>(1.27-6.01)	9.82<br>(2.69-12.63)	3.49<br>(1.53-4.45)	10.24<br>(4.36-13)	0.26<br>(0.2-0.32)
Palau	1.84<br>(1.41-2.21)	12.3<br>(9.46-14.81)	1.22<br>(0.83-1.5)	11.74<br>(7.66-14.53)	-0.15<br>(-0.17--0.12)
Papua New Guinea	919.44<br>(717.9-1153.27)	14.65<br>(11.46-18.29)	2127.35<br>(1706.75-2632.93)	14.33<br>(11.43-17.67)	-0.03<br>(-0.05--0.01)
Samoa	32.52<br>(26.65-38.48)	13.41<br>(11-15.85)	37.17<br>(30.28-44.49)	13.26<br>(10.77-15.84)	-0.08<br>(-0.11--0.05)
Solomon Islands	92.27<br>(74.11-111.68)	16.14<br>(12.9-19.5)	151<br>(119.95-183.4)	16.35<br>(13.05-19.83)	0.09<br>(0.07-0.11)
Tokelau	0.26<br>(0.21-0.31)	13.96<br>(11.31-16.63)	0.14<br>(0.11-0.17)	13.12<br>(10.34-15.85)	-0.23<br>(-0.24--0.22)
Tonga	19.14<br>(15.35-22.82)	13.32<br>(10.71-15.85)	18.02<br>(14.69-21.65)	13.13<br>(10.72-15.7)	-0.04<br>(-0.05--0.02)
Tuvalu	2.24<br>(1.84-2.72)	15.61<br>(12.83-18.89)	1.86<br>(1.51-2.22)	14.6<br>(11.85-17.4)	-0.19<br>(-0.23--0.15)
Vanuatu	37.21<br>(29.99-44.93)	14.51<br>(11.74-17.46)	59.99<br>(48.58-72.68)	14.75<br>(11.96-17.82)	0.03<br>(0-0.06)
**North Africa and Middle East**					
Afghanistan	3582.03<br>(2943.93-4351.86)	21.73<br>(18-26.33)	11972.73<br>(9986.72-14702.77)	23.18<br>(19.38-28.26)	0.16<br>(0.1-0.22)
Algeria	6624.44<br>(5194.48-8166.7)	18.98<br>(14.95-23.25)	9096.66<br>(7234.07-11269.45)	19.67<br>(15.71-24.27)	0.05<br>(0.02-0.07)
Bahrain	94.93<br>(77.24-116.2)	16.07<br>(13.1-19.53)	166.62<br>(130.63-203.16)	15.76<br>(12.4-19.22)	-0.08<br>(-0.1--0.07)
Egypt	14559.66<br>(12136.33-17176.25)	18.29<br>(15.33-21.49)	23439.98<br>(19144.66-27729.83)	18.77<br>(15.32-22.2)	0.06<br>(0.05-0.07)
Iran (Islamic Republic of)	16685.79<br>(13995.35-19708.64)	20.66<br>(17.4-24.33)	13106.39<br>(10722.42-15363.3)	19.99<br>(16.44-23.48)	-0.23<br>(-0.29--0.17)
Iraq	5250.44<br>(4170.11-6514.95)	18.08<br>(14.36-22.37)	7528.52<br>(6107.51-9123.54)	17.68<br>(14.34-21.35)	-0.26<br>(-0.36--0.16)
Jordan	1168.09<br>(909.27-1473.48)	20.96<br>(16.32-26.25)	2357.38<br>(1841.15-2919.92)	20.94<br>(16.37-25.96)	-0.12<br>(-0.16--0.08)
Kuwait	329.4<br>(264.13-407.6)	16.69<br>(13.53-20.38)	579.44<br>(452.05-704.26)	19.25<br>(15.03-23.86)	0.39<br>(0.31-0.48)
Lebanon	707.81<br>(555.29-876.43)	19.16<br>(15.08-23.65)	846.87<br>(673.31-1044.54)	19.45<br>(15.5-24.13)	-0.06<br>(-0.09--0.03)
Libya	864.88<br>(713.75-1028.46)	14.72<br>(12.16-17.39)	819.01<br>(649.9-987.11)	17.05<br>(13.52-20.65)	0.4<br>(0.33-0.47)
Morocco	5741.36	17.09	5690.92	17.09	-0.02

	(4770.94-6794.73)	(14.28-20.26)	(4589.95-6715.54)	(13.78-20.17)	(-0.04-0)
Palestine	696.93<br>(576.15-843.31)	19.83<br>(16.49-23.78)	1171.23<br>(959.69-1406.12)	19.84<br>(16.39-23.66)	-0.01<br>(-0.02-0)
Oman	496.26<br>(410.92-593.84)	16.54<br>(13.7-19.73)	694.44<br>(572.84-827.19)	16.06<br>(13.21-19.17)	-0.21<br>(-0.27--0.15)
Qatar	78.96<br>(60.23-98.89)	15.91<br>(12.1-19.99)	308.88<br>(171.5-388.44)	15.41<br>(8.22-19.46)	-0.2<br>(-0.23--0.17)
Saudi Arabia	3631.38<br>(2910.76-4375.38)	16.03<br>(12.97-19.26)	4017.65<br>(3056.57-4852.97)	14.95<br>(11.47-18.18)	-0.23<br>(-0.25--0.21)
Sudan	1242.59<br>(428.68-1611.49)	12.3<br>(4.4-16.01)	2063.22<br>(1408.79-2607.08)	13.32<br>(9.18-16.74)	0.19<br>(0.13-0.25)
Syrian Arab Republic	3578.31<br>(2982.56-4187.83)	18.18<br>(15.34-21.2)	2219.15<br>(1811.7-2652.33)	20.5<br>(16.67-24.66)	0.48<br>(0.44-0.52)
Tunisia	2011.64<br>(1621.76-2443.8)	19.76<br>(15.99-23.87)	1893.19<br>(1482.65-2296.88)	20.01<br>(15.62-24.44)	0.02<br>(0.01-0.03)
Turkey	12957.4<br>(10471.25-15917.83)	19.32<br>(15.61-23.7)	11161.99<br>(9228.84-13276.23)	18.4<br>(15.21-21.83)	-0.11<br>(-0.16--0.07)
United Arab Emirates	321.52<br>(231.47-399.14)	14.55<br>(10.52-18.15)	823.82<br>(641.3-1006.24)	15.71<br>(12.39-19.4)	0.27<br>(0.25-0.29)
Yemen	5061.39<br>(4198.15-6004.42)	19.76<br>(16.48-23.21)	9557.47<br>(7998.97-11330.08)	21.36<br>(17.91-25.29)	0.18<br>(0.15-0.21)
**South Asia**					
Bangladesh	29666.37<br>(22992.81-36884.56)	16.31<br>(12.65-20.31)	18548.04<br>(13741.91-23060.81)	12.86<br>(9.49-15.97)	-0.8<br>(-0.84--0.77)
Bhutan	155.98<br>(126.28-190.07)	16.86<br>(13.69-20.36)	98.22<br>(79.94-119.62)	15.62<br>(12.68-19.07)	-0.28<br>(-0.3--0.26)
India	217793.19<br>(180580.97-263387.21)	19.34<br>(16.07-23.36)	224622.78<br>(189214.5-263564.26)	19.62<br>(16.47-23.08)	0.19<br>(0.13-0.26)
Nepal	5068.59<br>(4109.93-6302.25)	15.98<br>(13.06-19.7)	4895.7<br>(4014.15-5965.97)	15.77<br>(12.93-19.23)	-0.06<br>(-0.12--0.01)
Pakistan	31810.57<br>(25983.87-39816.29)	17.94)<br>(14.73-22.22	54091.11<br>(44312.28-66810.41)	18.69<br>(15.38-23.05)	0.15<br>(0.13-0.17)
**Sub-Saharan Africa**					
**Southern sub-Saharan Africa**					
Botswana	283.79<br>(87.24-367.65)	13.91<br>(4.52-17.99)	275.77<br>(41.84-369.14)	11.68<br>(1.73-15.67)	-0.57<br>(-0.59--0.55)
Lesotho	374.77<br>(290.91-467.75)	16.21<br>(12.52-20.29)	318.5<br>(212.85-400.42)	15.79<br>(10.49-19.82)	-0.12<br>(-0.15--0.1)
Namibia	284.01<br>(111.17-364.58)	13.08<br>(5.48-16.81)	328.87<br>(77.6-424.7)	11.99<br>(2.87-15.49)	-0.4<br>(-0.45--0.35)
South Africa	5576.5<br>(1642.86-6968.4)	11.79<br>(3.57-14.75)	5750.26<br>(1477.36-7200.04)	11.43<br>(2.82-14.3)	-0.19<br>(-0.21--0.16)
Eswatini	202.34<br>(91.62-256.49)	14.84<br>(7.14-18.74)	193.06<br>(65.76-240.36)	14.09<br>(4.87-17.57)	-0.15<br>(-0.18--0.12)
Zimbabwe	2379.97	14.33	3301.82	15.67	0.46

	(1394.11-2987.97)	(8.64-17.84)	(2523.2-4190.87)	(11.97-19.82)	(0.38-0.54)
**Western sub-Saharan Africa**					
Benin	1484.34 (1101.47-1957.39)	15.68 (11.54-20.34)	3365.8 (2371.02-4188.53)	14.94 (10.56-18.53)	-0.15 (-0.16--0.14)
Burkina Faso	3104.65 (2366.89-3988.33)	17.13 (12.9-21.74)	6491.26 (4932.09-8164.65)	16.42 (12.51-20.56)	-0.13 (-0.15--0.11)
Cameroon	2923.87 (1621.51-3805.06)	15.17 (8.68-19.61)	6867.64 (4181.07-8639.67)	14.69 (9.02-18.4)	-0.13 (-0.16--0.1)
Cabo Verde	73.1 (55.27-88.61)	13.13 (9.86-15.87)	56.31 (22.5-71.65)	12.3 (4.71-15.69)	-0.28 (-0.33--0.22)
Chad	2188.72 (1676.48-2730.7)	18.19 (13.99-22.55)	5854.02 (4384.45-7384.83)	16.73 (12.48-20.92)	-0.35 (-0.39--0.31)
Côte d’Ivoire	3092.44 (1775.56-3944.34)	13.86 (8.18-17.78)	5735.41 (3348.51-7239.68)	13.63 (8.11-17.16)	-0.09 (-0.14--0.04)
Gambia	248.06 (184.48-308.85)	13.77 (10.19-17.02)	483.54 (361.37-589.28)	13.92 (10.39-16.93)	0.01 (0-0.03)
Ghana	4030.85 (2955.45-4910.56)	15.94 (11.68-19.42)	6909.68 (3222.22-8625.08)	15.31 (7.27-19.04)	-0.08 (-0.11--0.05)
Guinea	1895.49 (1333-2439.78)	16.78 (11.68-21.4)	3473.06 (2411.98-4408.13)	15.97 (11.12-20.19)	-0.13 (-0.17--0.09)
Guinea-Bissau	308.96 (226.82-392.39)	16.81 (12.29-21.3)	528.56 (403.77-670.09)	16.42 (12.47-20.78)	-0.09 (-0.11--0.08)
Liberia	725.07 (553.6-917.17)	16.2 (12.38-20.6)	1144.95 (909.81-1429.05)	15.41 (12.23-19.25)	-0.37 (-0.45--0.29)
Mali	2998.76 (2291.1-3858.02)	17.8 (13.6-22.86)	6876.08 (5038.7-8863.99)	15.51 (11.29-19.89)	-0.49 (-0.55--0.43)
Mauritania	456.07 (302.3-570.13)	12.67 (8.49-15.82)	760.73 (397.42-951.5)	11.96 (6.32-14.94)	-0.16 (-0.18--0.15)
Niger	3048.43 (2317.63-3942.55)	18.38 (13.81-23.6)	8437.09 (6556.71-10721.44)	17.18 (13.39-21.65)	-0.29 (-0.33--0.25)
Nigeria	23952.73 (13192.08-29706.9)	15.34 (8.69-18.85)	50330.74 (21969.68-62679.74)	14.04 (6.28-17.44)	-0.46 (-0.53--0.39)
São Tomé and Príncipe	27.02 (20.12-32.67)	13.83 (10.4-16.62)	33.22 (20.5-41.29)	13.55 (8.32-16.78)	-0.06 (-0.09--0.02)
Senegal	2053.48 (1347.36-2571.59)	14.55 (9.66-18.15)	3172.98 (1979.87-3959.28)	14.39 (9-17.93)	-0.07 (-0.09--0.06)
Sierra Leone	1268.16 (914.28-1638.04)	16.68 (12.18-21.42)	2111.49 (1630.97-2630.35)	16.16 (12.42-20.01)	-0.08 (-0.15--0.01)
Togo	1019.22 (755.94-1304.42)	15.47 (11.54-19.55)	1741.45 (1360.34-2132.88)	15.39 (11.99-18.81)	-0.01 (-0.04-0.01)
**Eastern sub-Saharan Africa**					
Burundi	1567.01 (1223.38-1927.1)	15.17 (11.75-18.57)	3139.68 (2529.11-3959.52)	15.16 (12.25-19.06)	-0.04 (-0.09-0)
Comoros	94.17 (62.44-118.05)	11.82 (7.91-14.81)	95.95 (71.37-118.4)	11.9 (8.84-14.67)	-0.01 (-0.03-0.02)

Djibouti	74.7 (42.6-90.99)	11.8 (6.91-14.39)	166.68 (101.67-208.21)	11.61 (7.09-14.49)	-0.06 (-0.12--0.01)
Eritrea	857.71 (668.72-1088.05)	14.28 (10.99-17.84)	1208.39 (924.7-1500.02)	13.55 (10.31-16.75)	-0.11 (-0.16--0.06)
Ethiopia	15670.25 (12568.96-19228.97)	16.71 (13.42-20.33)	21087.36 (16932.21-26132.06)	13.56 (10.88-16.68)	-0.81 (-0.89--0.73)
Kenya	5492.5 (4161.95-6543.94)	13.57 (10.32-16.08)	7798.58 (5451.05-9321.35)	13.36 (9.28-15.93)	-0.05 (-0.06--0.04)
Madagascar	2863.32 (2223.65-3612.18)	13.7 (10.63-17.14)	5261.65 (4194-6469.55)	13.32 (10.58-16.39)	-0.13 (-0.14--0.11)
Malawi	2712.78 (2136.21-3400.65)	14.64 (11.62-18.27)	3637.32 (2924.32-4492.98)	13.77 (11.07-16.94)	-0.24 (-0.27--0.22)
Mozambique	3667.19 (2902.84-4488.47)	15.74 (12.57-19.15)	6744.7 (5304.93-8307.38)	13.62 (10.58-16.76)	-0.56 (-0.59--0.53)
Rwanda	1933.71 (1518.94-2416.34)	15.07 (11.73-18.62)	2319.76 (1834-2837.9)	13.66 (10.79-16.65)	-0.48 (-0.55--0.41)
Somalia	2660.21 (2125.06-3318.34)	17.73 (14.26-21.95)	7256.99 (5874.39-9072.23)	18.26 (14.8-22.71)	0.05 (0.02-0.09)
South Sudan	1242.59 (428.68-1611.49)	12.3 (4.4-16.01)	2063.22 (1408.79-2607.08)	13.32 (9.18-16.74)	0.19 (0.13-0.25)
United Republic of Tanzania	6065.32 (4737.53-7561.19)	13.12 (10.2-16.25)	10336.16 (7540.69-12713)	12.16 (8.95-14.9)	-0.32 (-0.36--0.28)
Uganda	4865.36 (3838.9-5938.6)	14.18 (11.23-17.26)	9277.01 (6856.88-11215.92)	13.3 (9.91-16.09)	-0.26 (-0.28--0.23)
Zambia	1986.82 (1395.96-2473.96)	13.48 (9.5-16.71)	3535.47 (2334.74-4425.72)	12.55 (8.26-15.65)	-0.34 (-0.42--0.26)
**Central sub-Saharan Africa**					
Angola	3020.5 (1881.64-3893.34)	15.71 (9.97-20.19)	7626.61 (3001.55-9751.8)	14.07 (5.74-17.92)	-0.52 (-0.58--0.45)
Central African Republic	916.27 (700.5-1169.31)	18.61 (14.28-23.58)	1535.9 (1193.06-1921.06)	18.79 (14.62-23.41)	0.03 (0.01-0.05)
Congo	528.14 (265.69-679.57)	13.78 (7.14-17.59)	825.82 (422.26-1026.75)	13.3 (6.77-16.55)	-0.19 (-0.21--0.16)
Democratic Republic of the Congo	11502.25 (8698.91-14841.94)	16.26 (12.19-20.83)	21376.97 (16680.34-26708.01)	16.45 (12.9-20.55)	-0.01 (-0.14-0.12)
Equatorial Guinea	120.64 (88.3-154.81)	15.11 (10.95-19.36)	173.81 (23.39-239.89)	9.44 (1.26-13.04)	-2.34 (-2.7--1.98)
Gabon	158.42(32.38-209.97)	10.43 (2.29-13.94)	214.35 (39.39-278.01)	10.2 (1.88-13.24)	-0.1 (-0.11--0.08)

Abbreviation: EAPC, estimated annual percentage changes; IDD, intellectual developmental disability; CHD, congenital heart disease; ASPR, age-standardized prevalence rates; UI, uncertainty intervals; CI, confidence intervals; SDI, social demographic index.

STable 4. The DALYs in all-aged number and age-standardized rates and EAPC of IDD attributable to CHD across 204 countries and territories from 1990 to 2021

	1990		2021		1990-2021
	Number	ASPR per 100,000	Number	ASPR per 100,000	EAPC
	No.(95%UI)	No.(95%UI)	No.(95%UI)	No.(95%UI)	No.(95%CI)
**High-income**					
**High-income North America**					
Canada	77.09 (37.84-124.37)	0.38 (0.18-0.62)	82.76 (51.28-123.26)	0.39 (0.24-0.59)	-0.34 (-0.47--0.2)
Greenland	0.18 (0.11-0.28)	0.33 (0.2-0.51)	0.12 (0.08-0.19)	0.3 (0.18-0.45)	-0.51 (-0.58--0.45)
United States of America	769.93 (502.41-1167.59)	0.37 (0.24-0.57)	737.36 (474.49-1101.7)	0.36 (0.23-0.54)	-0.12 (-0.16--0.08)
**Australasia**					
Australia	14.8 (2.6-32.29)	0.11 (0.02-0.24)	41.59 (21.48-69.92)	0.25 (0.12-0.42)	2.55 (2.09-3.01)
New Zealand	4.43 (1.06-8.75)	0.15 (0.03-0.31)	9.82 (5.01-15.74)	0.28 (0.14-0.46)	2.32 (1.7-2.94)
**High-income Asia Pacific**					
Brunei Darussalam	0.23 (0-0.72)	0.07 (0-0.22)	0.13 (0-0.49)	0.04 (0-0.15)	-2.47 (-2.78--2.15)
Japan	221.09 (89.68-386.39)	0.26 (0.09-0.46)	90.08 (6.2-193.12)	0.13 (0.01-0.29)	-2.18 (-2.28--2.09)
Singapore	3.56 (0.61-7.37)	0.16 (0.02-0.34)	0.93 (0-4.4)	0.02 (0-0.13)	-5.61 (-6.08--5.14)
Republic of Korea	139.57 (72.64-223.37)	0.39 (0.19-0.62)	13.56 (0-44.85)	0.05 (0-0.19)	-6.22 (-6.48--5.97)
**Western Europe**					
Andorra	0.1 (0.05-0.16)	0.28 (0.14-0.46)	0.12 (0.06-0.19)	0.28 (0.13-0.45)	-0.26 (-0.48--0.04)
Austria	24.99 (15.24-36.95)	0.45 (0.27-0.66)	22.82 (12.35-35.07)	0.39 (0.19-0.61)	-0.73 (-0.86--0.61)
Belgium	35.03 (21.33-53.86)	0.47 (0.28-0.73)	26.97 (15.31-41.87)	0.35 (0.18-0.55)	-0.98 (-1.15--0.8)
Cyprus	3.05 (1.92-4.72)	0.45 (0.28-0.69)	3.63 (2.1-5.65)	0.39 (0.22-0.62)	-0.42 (-0.54--0.31)
Denmark	4.15 (1.28-8.98)	0.1 (0.02-0.24)	3.35 (0.93-6.9)	0.08 (0.01-0.17)	-1.26 (-1.34--1.17)
Finland	20.58 (12.16-32.1)	0.56 (0.32-0.89)	16.2 (9.85-24.99)	0.51 (0.3-0.8)	-0.46 (-0.74--0.18)
France	284.35 (170.15-436.53)	0.65 (0.38-0.99)	242.88 (140.01-379.74)	0.56 (0.32-0.88)	-0.42 (-0.49--0.35)
Germany	197.48 (107.69-300.96)	0.36 (0.19-0.57)	139.22 (53.81-237.92)	0.26 (0.08-0.45)	-0.97 (-1.06--0.88)

Greece	34.61 (20.79-52.63)	0.48 (0.27-0.74)	20.54 (12.06-31.6)	0.35 (0.19-0.55)	-1.17 (-1.29--1.05)
Iceland	0.7 (0.38-1.08)	0.31 (0.17-0.48)	0.67 (0.32-1.08)	0.25 (0.12-0.42)	-0.79 (-0.89--0.69)
Ireland	11.97 (6.68-19.1)	0.38 (0.21-0.6)	8.23 (3.57-13.85)	0.22 (0.09-0.39)	-2.21 (-2.91--1.5)
Israel	20.11 (12.11-30.95)	0.39 (0.24-0.61)	34.84 (20.45-52.73)	0.38 (0.22-0.57)	-0.26 (-0.29--0.23)
Italy	149.77 (88.79-228.38)	0.41 (0.23-0.63)	127.89 (76.25-193.66)	0.39 (0.22-0.6)	-0.15 (-0.46-0.16)
Luxembourg	1 (0.57-1.56)	0.36 (0.2-0.56)	1.19 (0.6-1.9)	0.28 (0.13-0.47)	-0.79 (-0.94--0.63)
Malta	1.31 (0.79-1.99)	0.42 (0.25-0.63)	0.84 (0.43-1.35)	0.29 (0.14-0.49)	-1.14 (-1.24--1.04)
Monaco	0.03 (0.01-0.05)	0.15 (0.04-0.31)	0.03 (0.01-0.06)	0.11 (0.02-0.25)	-0.85 (-0.96--0.73)
Netherlands	37.31 (19.88-58.08)	0.33 (0.16-0.53)	24.88 (12.13-42.06)	0.21 (0.09-0.37)	-1.54 (-1.74--1.34)
Norway	11.22 (6.5-17.18)	0.34 (0.19-0.53)	11.87 (6.65-18.7)	0.33 (0.17-0.52)	-0.56 (-0.71--0.41)
Portugal	30.01 (18.7-45.86)	0.42 (0.25-0.65)	21.23 (13.09-33.2)	0.37 (0.22-0.59)	-0.4 (-0.47--0.33)
San Marino	0.05 (0.03-0.08)	0.3 (0.16-0.48)	0.06 (0.03-0.09)	0.3 (0.14-0.48)	-0.06 (-0.44-0.32)
Spain	223.06 (142.35-339.14)	0.84 (0.54-1.28)	207.15 (127.07-305.79)	0.79 (0.48-1.17)	-0.51 (-0.61--0.41)
Sweden	27.17 (15.71-43.56)	0.44 (0.25-0.71)	26.57 (16.58-40.96)	0.4 (0.25-0.61)	-0.79 (-1.25--0.33)
Switzerland	18.07 (10.02-27.82)	0.36 (0.19-0.58)	17.51 (8.84-27.82)	0.3 (0.14-0.49)	-0.8 (-0.89--0.71)
United Kingdom	170.39 (108.57-250.95)	0.38 (0.24-0.56)	159.06 (100.6-238.94)	0.35 (0.22-0.53)	-0.15 (-0.26--0.03)
**Southern Latin America**					
Argentina	117.37 (72.46-176.24)	0.35 (0.21-0.52)	105.61 (68.01-158.25)	0.33 (0.21-0.5)	-0.27 (-0.37--0.18)
Chile	62.24 (38.62-93.7)	0.44 (0.27-0.66)	43.94 (26.77-66.65)	0.37 (0.22-0.56)	-0.78 (-0.97--0.58)
Uruguay	11.1 (7.03-17.01)	0.4 (0.25-0.61)	7.92 (4.81-12.28)	0.37 (0.23-0.58)	-0.18 (-0.23--0.13)
**Central Europe, eastern Europe, and central Asia**					
**Eastern Europe**					
Belarus	42.54 (22.14-69.86)	0.5 (0.26-0.83)	25.22 (12.92-42.7)	0.46 (0.23-0.79)	-0.5 (-0.63--0.37)
Estonia	5.54 (2.75-9.35)	0.44 (0.21-0.75)	2.64 (1.12-4.63)	0.34 (0.14-0.6)	-1.23 (-1.4--1.06)

Latvia	9.52 (4.77-15.87)	0.45 (0.22-0.75)	3.98 (1.95-6.91)	0.36 (0.17-0.65)	-0.87 (-0.99--0.75)
Lithuania	13.68 (6.59-23.51)	0.45 (0.22-0.78)	5.29 (2.46-9.32)	0.34 (0.15-0.63)	-1.2 (-1.35--1.05)
Republic of Moldova	23.63 (12.26-37.44)	0.55 (0.28-0.88)	10.34 (5.8-15.83)	0.55 (0.29-0.87)	-0.15 (-0.32-0.02)
Russian Federation	448.18 (235.32-748.54)	0.37 (0.19-0.62)	327.91 (167-524.74)	0.38 (0.19-0.61)	-0.25 (-0.46--0.04)
Ukraine	209.84 (106.83-338.47)	0.52 (0.26-0.85)	113.42 (62.3-180.46)	0.55 (0.29-0.89)	-0.08 (-0.21-0.05)
**Central Europe**					
Albania	18.8 (9.98-29.59)	0.48 (0.26-0.75)	7.03 (3.65-11.18)	0.44 (0.22-0.71)	-0.41 (-0.47--0.36)
Bosnia and Herzegovina	22.42 (13.22-34.98)	0.61 (0.36-0.96)	7.89 (3.85-12.6)	0.45 (0.22-0.72)	-1.21 (-1.3--1.12)
Bulgaria	31.6 (17.35-50.29)	0.54 (0.29-0.87)	16.04 (7.93-26.91)	0.46 (0.21-0.77)	-0.37 (-0.51--0.23)
Croatia	12.21 (5.89-20.81)	0.36 (0.17-0.63)	6.52 (3.01-11.81)	0.29 (0.12-0.54)	-0.84 (-1--0.69)
Czechia	26.52 (12.2-46.65)	0.37 (0.17-0.66)	19.8 (8.19-37.23)	0.32 (0.13-0.6)	-1.03 (-1.29--0.77)
Hungary	25.82 (12.64-43.38)	0.37 (0.17-0.64)	16.05 (6.88-28.56)	0.3 (0.12-0.56)	-1.03 (-1.17--0.88)
North Macedonia	7.64 (3.97-12.34)	0.44 (0.22-0.71)	4.53 (2.29-7.55)	0.38 (0.18-0.64)	-0.6 (-0.67--0.53)
Montenegro	1.79 (0.89-3.02)	0.33 (0.16-0.55)	1.24 (0.6-2.1)	0.31 (0.14-0.53)	-0.62 (-0.79--0.44)
Poland	169.96 (92.02-267.01)	0.55 (0.3-0.87)	92.28 (46.38-150.14)	0.44 (0.22-0.73)	-0.92 (-1.06--0.79)
Romania	83.91 (42.73-134.27)	0.46 (0.23-0.73)	38.35 (18-63.44)	0.37 (0.17-0.61)	-0.89 (-0.99--0.79)
Serbia	27.88 (14.34-47.51)	0.38 (0.19-0.65)	17.93 (9.15-28.39)	0.4 (0.19-0.65)	0.04 (-0.1-0.17)
Slovakia	18.11 (8.84-29.68)	0.42 (0.2-0.69)	10.09 (4.3-17.89)	0.32 (0.13-0.57)	-1.04 (-1.09--0.99)
Slovenia	4.93 (2.28-8.57)	0.36 (0.16-0.64)	3.45 (1.47-6.25)	0.3 (0.11-0.56)	-0.72 (-0.85--0.59)
**Central Asia**					
Armenia	27.68 (16-42.38)	0.75 (0.44-1.14)	15.06 (8.09-22.96)	0.72 (0.38-1.1)	-0.31 (-0.4--0.21)
Azerbaijan	54.72 (30.14-86.38)	0.63 (0.35-0.99)	45.39 (24.24-71.33)	0.58 (0.31-0.93)	-0.62 (-0.82--0.41)
Georgia	27 (14.83-40.55)	0.56 (0.3-0.84)	15.36 (8.63-23.44)	0.58 (0.32-0.9)	-0.02 (-0.17-0.13)
Kazakhstan	113.17	0.62	106.64	0.55	-0.56

	(59.99-179.33)	(0.33-0.98)	(53.12-173.65)	(0.28-0.9)	(-0.68--0.43)
Kyrgyzstan	41.43	0.7	56.8	0.74	0.1
	(23.57-65.65)	(0.4-1.09)	(34.24-87.56)	(0.45-1.14)	(-0.1-0.3)
Mongolia	27.23	0.86	25.55	0.68	-1.07
	(15.65-44.02)	(0.49-1.38)	(13.15-41)	(0.35-1.08)	(-1.17--0.97)
Tajikistan	62.25	0.71	98.28	0.77	-0.16
	(36.1-96.89)	(0.42-1.11)	(56.85-157.47)	(0.45-1.23)	(-0.51-0.19)
Turkmenistan	36.57	0.68	31.87	0.6	-0.67
	(19.86-57.83)	(0.38-1.06)	(16.86-52.32)	(0.32-0.98)	(-0.81--0.54)
Uzbekistan	228.55	0.73	263.86	0.71	-0.4
	(132.11-374.16)	(0.43-1.2)	(144.13-421.44)	(0.39-1.12)	(-0.5--0.3)
**Latin America and Caribbean**					
**Central Latin America**					
Colombia	150.24	0.38	113.07	0.3	-0.91
	(75.89-248.24)	(0.19-0.62)	(52.74-189.32)	(0.14-0.51)	(-1.01--0.81)
Costa Rica	13.79	0.36	11.45	0.32	-0.41
	(6.52-23.88)	(0.17-0.62)	(5.58-19.65)	(0.15-0.57)	(-0.51--0.3)
El Salvador	30.64	0.43	22.33	0.36	-0.51
	(16.16-48.76)	(0.23-0.68)	(11.18-37.44)	(0.18-0.61)	(-0.55--0.46)
Guatemala	48.88	0.35	50.15	0.32	-0.33
	(26.07-75.61)	(0.19-0.54)	(23.47-82.7)	(0.15-0.53)	(-0.35--0.3)
Honduras	31.65	0.42	43.58	0.4	-0.16)
	(17.53-51.86)	(0.24-0.69)	(22.36-68.21)	(0.21-0.63)	(-0.19--0.13
Mexico	386.96	0.35	373.07	0.35	-0.09
	(201.25-636.44)	(0.19-0.58)	(192.26-595.94)	(0.18-0.56)	(-0.21-0.03)
Nicaragua	22.49	0.38	24.42	0.37	-0.11
	(11.78-35.81)	(0.2-0.6)	(12.87-38.41)	(0.2-0.59)	(-0.16--0.05)
Panama	10.2	0.37	10.2	0.26	-1.07
	(4.9-16.9)	(0.18-0.61)	(4.3-18.34)	(0.11-0.48)	(-1.15--0.99)
Venezuela (Bolivarian Republic of)	74.91	0.32	75.44	0.33	-0.06
	(36.04-127.06)	(0.16-0.54)	(33.43-129.26)	(0.15-0.56)	(-0.2-0.07)
**Andean Latin America**					
Bolivia (Plurinational State of)	40.29	0.44	46.91	0.39	-0.48
	(24.43-61.37)	(0.28-0.67)	(25.15-72.75)	(0.21-0.61)	(-0.5--0.46)
Ecuador	49.84	0.4	66.29	0.39	-0.07
	(26.54-78.7)	(0.21-0.62)	(35.27-103.24)	(0.2-0.61)	(-0.12--0.02)
Peru	105.97	0.39	116.74	0.35	-0.45
	(59.17-165.4)	(0.22-0.6)	(59.96-185.23)	(0.17-0.55)	(-0.48--0.43)
**Caribbean**					
Antigua and Barbuda	0.2	0.33	0.17	0.27	-0.55
	(0.09-0.34)	(0.14-0.55)	(0.07-0.28)	(0.12-0.47)	(-0.61--0.48)
Bahamas	0.61	0.24	0.6	0.24	-0.09
	(0.24-1.12)	(0.09-0.44)	(0.24-1.05)	(0.09-0.42)	(-0.23-0.06)
Barbados	0.68	0.33	0.54	0.33	0.06
	(0.31-1.14)	(0.15-0.55)	(0.26-0.91)	(0.15-0.56)	(-0.08-0.19)

Belize	0.96 (0.5-1.5)	0.36 (0.19-0.56)	1.25 (0.64-2.01)	0.32 (0.16-0.51)	-0.43 (-0.47--0.4)
Bermuda	0.11 (0.04-0.22)	0.25 (0.08-0.47)	0.06 (0.02-0.12)	0.18 (0.04-0.38)	-1.3 (-1.44--1.16)
Cuba	38.89 (18.37-62.37)	0.42 (0.2-0.67)	28.3 (14.73-45.42)	0.43 (0.22-0.69)	-0.11 (-0.23-0.01)
Dominica	0.28 (0.14-0.44)	0.33 (0.17-0.53)	0.12 (0.06-0.2)	0.29 (0.14-0.47)	-0.44 (-0.47--0.41)
Dominican Republic	28.49 (13.16-45.5)	0.3 (0.14-0.48)	23.11 (10.81-38.22)	0.22 (0.1-0.36)	-1.19 (-1.26--1.12)
Grenada	0.44 (0.22-0.69)	0.39 (0.2-0.61)	0.24 (0.12-0.4)	0.32 (0.15-0.53)	-0.65 (-0.69--0.61)
Guyana	3.82 (2.18-5.98)	0.36 (0.21-0.56)	2.49 (1.29-3.94)	0.33 (0.17-0.53)	-0.21 (-0.25--0.17)
Haiti	35.79 (16.36-59.54)	0.36 (0.17-0.6)	61.06 (33.6-98.09)	0.4 (0.22-0.65)	0.34 (0.29-0.39)
Jamaica	9.28 (4.49-15.02)	0.34 (0.17-0.55)	6.5 (3.01-10.47)	0.33 (0.15-0.54)	-0.13 (-0.16--0.1)
Puerto Rico	8.91 (3.75-16.12)	0.27 (0.11-0.49)	3.3 (1.25-6.01)	0.22 (0.07-0.41)	-0.87 (-1.23--0.5)
Saint Kitts and Nevis	0.14 (0.07-0.24)	0.31 (0.15-0.52)	0.09 (0.04-0.15)	0.23 (0.11-0.4)	-0.92 (-0.97--0.87)
Saint Lucia	0.54 (0.24-0.85)	0.32 (0.15-0.51)	0.3 (0.12-0.48)	0.28 (0.12-0.46)	-0.42 (-0.43--0.4)
Saint Vincent and the Grenadines	0.51 (0.26-0.8)	0.4 (0.21-0.64)	0.27 (0.13-0.42)	0.33 (0.16-0.53)	-0.68 (-0.7--0.66)
Suriname	1.31 (0.64-2.11)	0.3 (0.15-0.49)	1.33 (0.6-2.23)	0.28 (0.13-0.47)	-0.47 (-0.54--0.41)
Trinidad and Tobago	3.75 (1.7-6.46)	0.28 (0.13-0.49)	1.76 (0.61-3.47)	0.19 (0.06-0.39)	-1.64 (-1.78--1.5)
United States Virgin Islands	0.3 (0.13-0.52)	0.27 (0.12-0.48)	0.09 (0.03-0.18)	0.19 (0.05-0.38)	-1.2 (-1.55--0.85)
**Tropical Latin**					
Brazil	621.1 (366.89-966.75)	0.39 (0.23-0.6)	556.9 (301.14-852.26)	0.31 (0.17-0.48)	-0.71 (-0.8--0.62)
Paraguay	22.18 (11.41-36.4)	0.39 (0.21-0.64)	25.22 (13.53-40.79)	0.38 (0.2-0.61)	-0.1 (-0.16--0.05)
**Southeast Asia, east Asia, and Oceania**					
**East Asia**					
China	4959.84 (2896.26-7646)	0.44 (0.26-0.68)	3179.65 (1798.4-4881.54)	0.35 (0.19-0.55)	-0.83 (-0.89--0.78)
Democratic People's Republic of Korea	88.5 (48.75-135.09)	0.39 (0.22-0.59)	85.61 (50.89-134.65)	0.49 (0.29-0.77)	0.7 (0.62-0.77)
Taiwan (Province of China)	53.49 (24.87-87.33)	0.31 (0.14-0.5)	18.1 (4.73-36.35)	0.14 (0.03-0.3)	-2.47 (-2.6--2.34)

**Southeast Asia**					
Cambodia	122.46 (78.6-185.96)	0.72 (0.46-1.08)	96.74 (60.82-145.33)	0.56 (0.35-0.84)	-0.93 (-1.02--0.84)
Indonesia	1489.96 (967.46-2244.52)	0.69 (0.45-1.03)	1045.63 (663.76-1569.32)	0.45 (0.29-0.68)	-1.35 (-1.4--1.3)
Lao People's Democratic Republic	52.28 (33.04-78.52)	0.78 (0.49-1.16)	42.37 (26.76-64.09)	0.52 (0.33-0.79)	-1.42 (-1.5--1.34)
Malaysia	92.92 (57.74-142.51)	0.41 (0.26-0.63)	86.45 (45.98-140.75)	0.33 (0.18-0.54)	-0.69 (-0.74--0.64)
Maldives	1.63 (1.03-2.51)	0.44 (0.28-0.66)	1.15 (0.66-1.79)	0.31 (0.18-0.49)	-1.07 (-1.12--1.03)
Mauritius	4.86 (2.94-7.35)	0.45 (0.27-0.68)	2.47 (1.36-3.79)	0.32 (0.17-0.51)	-1.11 (-1.19--1.04)
Myanmar	423.39 (271.98-630.04)	0.86 (0.55-1.27)	271.86 (171-412.34)	0.51 (0.32-0.78)	-1.93 (-2.09--1.76)
Philippines	511.43 (327.68-759.72)	0.58 (0.37-0.86)	504.08 (319.16-739.15)	0.45 (0.28-0.66)	-0.8 (-0.86--0.74)
Sri Lanka	80.78 (51.02-122.62)	0.46 (0.29-0.69)	61.33 (35.87-93.79)	0.36 (0.21-0.55)	-0.73 (-0.75--0.72)
Seychelles	0.33 (0.19-0.51)	0.42 (0.24-0.65)	0.29 (0.15-0.46)	0.34 (0.18-0.56)	-0.5 (-0.55--0.45)
Thailand	351.72 (216.79-521.61)	0.66 (0.41-0.98)	155.56 (97.4-235.55)	0.44 (0.27-0.67)	-1.32 (-1.37--1.27)
Timor-Leste	7.74 (4.97-11.6)	0.59 (0.37-0.88)	8.34 (5.08-12.5)	0.47 (0.28-0.7)	-1.09 (-1.26--0.92)
Viet Nam	486.44 (315-718.28)	0.55 (0.35-0.8)	314.72 (179.96-492.74)	0.37 (0.21-0.58)	-1.24 (-1.32--1.17)
**Oceania**					
American Samoa	0.19 (0.09-0.32)	0.28 (0.13-0.45)	0.13 (0.07-0.2)	0.32 (0.16-0.5)	0.38 (0.32-0.44)
Cook Islands	0.07 (0.03-0.11)	0.32 (0.15-0.52)	0.03 (0.02-0.05)	0.26 (0.12-0.43)	-0.58 (-0.63--0.53)
Micronesia (Federated States of)	0.66 (0.41-1.06)	0.45 (0.28-0.72)	0.46 (0.28-0.73)	0.48 (0.3-0.75)	0.21 (0.14-0.27)
Fiji	3.58 (1.99-5.52)	0.4 (0.22-0.61)	3.25 (1.72-5.17)	0.36 (0.19-0.57)	-0.29 (-0.31--0.27)
Guam	0.37 (0.17-0.63)	0.24 (0.11-0.4)	0.29 (0.12-0.49)	0.22 (0.09-0.37)	-0.52 (-0.57--0.46)
Kiribati	0.56 (0.33-0.91)	0.51 (0.3-0.82)	0.8 (0.45-1.22)	0.57 (0.32-0.87)	0.44 (0.38-0.5)
Marshall Islands	0.32 (0.2-0.5)	0.46 (0.29-0.74)	0.27 (0.17-0.43)	0.47 (0.3-0.75)	0.1 (0.05-0.16)
Nauru	0.05 (0.02-0.08)	0.32 (0.16-0.52)	0.05 (0.03-0.07)	0.37 (0.21-0.55)	0.41 (0.18-0.64)

Niue	0.01<br>(0.01-0.01)	0.37<br>(0.22-0.56)	0<br>(0-0.01)	0.33<br>(0.18-0.51)	-0.49<br>(-0.55--0.43)
Northern Mariana Islands	0.11<br>(0.05-0.19)	0.23<br>(0.1-0.4)	0.09<br>(0.04-0.15)	0.26<br>(0.12-0.41)	0.57<br>(0.45-0.7)
Palau	0.05<br>(0.03-0.08)	0.35<br>(0.19-0.56)	0.04<br>(0.02-0.06)	0.33<br>(0.18-0.52)	-0.2<br>(-0.24--0.16)
Papua New Guinea	27.66<br>(16.15-45.34)	0.46<br>(0.27-0.74)	61.31<br>(35.86-95.79)	0.42<br>(0.25-0.67)	-0.11<br>(-0.16--0.07)
Samoa	1<br>(0.63-1.54)	0.42<br>(0.27-0.65)	1.12<br>(0.68-1.72)	0.41<br>(0.25-0.62)	-0.18<br>(-0.23--0.12)
Solomon Islands	3.2<br>(1.86-4.94)	0.58<br>(0.34-0.88)	5.37<br>(3.21-8.47)	0.59<br>(0.35-0.93)	0.24<br>(0.18-0.3)
Tokelau	0.01<br>(0-0.01)	0.43<br>(0.26-0.66)	0<br>(0-0.01)	0.39<br>(0.24-0.61)	-0.34<br>(-0.36--0.33)
Tonga	0.61<br>(0.37-0.95)	0.44<br>(0.27-0.68)	0.55<br>(0.34-0.85)	0.41<br>(0.25-0.64)	-0.14<br>(-0.17--0.1)
Tuvalu	0.08<br>(0.05-0.12)	0.56<br>(0.34-0.85)	0.06<br>(0.04-0.1)	0.48<br>(0.3-0.75)	-0.34<br>(-0.43--0.25)
Vanuatu	1.21<br>(0.73-1.92)	0.49<br>(0.3-0.77)	2<br>(1.21-3.15)	0.5<br>(0.3-0.78)	0.06<br>(-0.01-0.12)
**North Africa and Middle East**					
Afghanistan	140.45<br>(90.47-209.3)	0.88<br>(0.56-1.3)	504.52<br>(316.23-790.51)	1<br>(0.63-1.55)	0.26<br>(0.09-0.42)
Algeria	240.03<br>(143.76-369.26)	0.71<br>(0.43-1.08)	328.62<br>(188.71-512.39)	0.71<br>(0.41-1.12)	-0.17<br>(-0.23--0.11)
Bahrain	2.97<br>(1.77-4.64)	0.51<br>(0.31-0.8)	5.4<br>(3.31-8.12)	0.48<br>(0.29-0.73)	-0.25<br>(-0.27--0.23)
Egypt	581.03<br>(382.71-864.48)	0.75<br>(0.5-1.11)	925.78<br>(574.89-1397.04)	0.75<br>(0.47-1.13)	-0.06<br>(-0.08--0.04)
Iran (Islamic Republic of)	689.17<br>(434.94-1039.4)	0.87<br>(0.55-1.31)	495.96<br>(296.79-760.75)	0.73<br>(0.44-1.14)	-0.84<br>(-0.99--0.68)
Iraq	179.29<br>(105.22-276.71)	0.64<br>(0.38-1)	252.34<br>(155.42-397.02)	0.59<br>(0.37-0.93)	-0.68<br>(-0.9--0.45)
Jordan	42.95<br>(25.09-68.05)	0.8<br>(0.47-1.26)	85.46<br>(49.05-138.13)	0.75<br>(0.43-1.22)	-0.48<br>(-0.58--0.37)
Kuwait	10<br>(5.73-15.35)	0.52<br>(0.3-0.79)	18.18<br>(10.24-28.14)	0.57<br>(0.3-0.9)	0.18<br>(0.1-0.26)
Lebanon	25.09<br>(14.71-39.46)	0.7<br>(0.41-1.1)	31.22<br>(18.14-49.03)	0.69<br>(0.4-1.09)	-0.26<br>(-0.34--0.18)
Libya	25.88<br>(14.83-40.88)	0.46<br>(0.27-0.72)	31.69<br>(18.54-49.49)	0.63<br>(0.37-0.99)	0.87<br>(0.68-1.07)
Morocco	228.68<br>(144.62-346.86)	0.69<br>(0.44-1.05)	222.31<br>(134.72-335.29)	0.66<br>(0.4-0.99)	-0.19<br>(-0.23--0.16)
Palestine	28.51	0.85	48.27	0.83	-0.06

	(18.25-44.82)	(0.54-1.32)	(30.01-74.56)	(0.52-1.28)	(-0.08--0.04)
Oman	15.37	0.54	21.8	0.5	-0.42
	(9.27-24.36)	(0.33-0.85)	(12.67-33.62)	(0.29-0.77)	(-0.5--0.34)
Qatar	2.23	0.46	8.71	0.41	-0.61
	(1.18-3.46)	(0.24-0.71)	(4.51-13.75)	(0.2-0.65)	(-0.68--0.53)
Saudi Arabia	100.98	0.46	123.44	0.43	-0.29
	(54.43-156.55)	(0.26-0.72)	(68.53-195.5)	(0.23-0.69)	(-0.32--0.25)
Sudan	29.29	0.3	54.96	0.36	0.46
	(13.42-49.73)	(0.14-0.5)	(30.16-88.39)	(0.2-0.58)	(0.32-0.59)
Syrian Arab Republic	142.08	0.75	91.67	0.82	0.38
	(91.37-214.41)	(0.48-1.12)	(56.44-135.88)	(0.5-1.23)	(0.34-0.43)
Tunisia	79.26	0.79	72.45	0.75	-0.24
	(49.21-121.47)	(0.5-1.21)	(42.62-111.22)	(0.44-1.14)	(-0.28--0.21)
Turkey	438.78	0.66	362.37	0.57	-0.44
	(254.94-683.53)	(0.39-1.02)	(217.61-564.02)	(0.34-0.88)	(-0.5--0.38)
United Arab Emirates	8.39	0.39	25.87	0.44	0.56
	(4.43-14.17)	(0.21-0.65)	(14.31-40.19)	(0.23-0.69)	(0.48-0.64)
Yemen	195.32	0.79	402.71	0.91	0.29
	(128.04-292.91)	(0.52-1.17)	(253.71-616.21)	(0.57-1.38)	(0.2-0.37)
**South Asia**					
Bangladesh	1000.57	0.56	460.77	0.32	-1.95
	(563.57-1587.72)	(0.31-0.88)	(226.19-759.15)	(0.15-0.52)	(-2.05--1.86)
Bhutan	6.02	0.66	3.29	0.51	-0.89
	(3.73-9.13)	(0.41-1)	(1.98-5.18)	(0.31-0.81)	(-0.93--0.86)
India	10330.45	0.92	10769.4	0.93	0.15
	(6641.24-15302.08)	(0.59-1.36)	(7014.08-15679.24)	(0.61-1.36)	(0.08-0.21)
Nepal	193.94	0.63	195.82	0.63	0
	(121.59-282.52)	(0.4-0.93)	(122-294.36)	(0.39-0.95)	(-0.06-0.06)
Pakistan	1261.25	0.72	2245.53	0.78	0.28
	(766.88-1915.57)	(0.44-1.09)	(1389.11-3407.68)	(0.48-1.18)	(0.22-0.33)
**Sub-Saharan Africa**					
**Southern sub-Saharan Africa**					
Botswana	6.3	0.32	5.53	0.23	-1.18
	(2.66-11.08)	(0.14-0.56)	(2.05-10.26)	(0.08-0.43)	(-1.25--1.1)
Lesotho	9.91	0.44	8.31	0.41	-0.38
	(5.29-16.9)	(0.25-0.75)	(4.29-13.59)	(0.21-0.67)	(-0.44--0.32)
Namibia	6.42	0.31	6.96	0.26	-0.84
	(2.71-11.3)	(0.13-0.55)	(2.74-12.56)	(0.1-0.46)	(-0.94--0.73)
South Africa	124.42	0.27	136.46	0.27	-0.21
	(59.13-215.02)	(0.13-0.46)	(64.34-228.86)	(0.12-0.45)	(-0.26--0.17)
Eswatini	4.86	0.37	4.76	0.35	-0.12
	(2.37-8.36)	(0.18-0.63)	(2.14-7.86)	(0.16-0.58)	(-0.21--0.03)
Zimbabwe	61.91	0.39	91.58	0.45	0.72
	(31.73-102.62)	(0.21-0.65)	(48.47-144.31)	(0.24-0.7)	(0.58-0.85)
**Western sub-Saharan Africa**					

Benin	40.92 (23.8-67.94)	0.45 (0.26-0.73)	90 (47.52-143.01)	0.41 (0.22-0.64)	-0.34 (-0.37--0.31)
Burkina Faso	93.49 (52.15-147.25)	0.54 (0.3-0.84)	178.38 (98.26-279.28)	0.47 (0.26-0.72)	-0.5 (-0.57--0.43)
Cameroon	77.21 (37.43-129.66)	0.42 (0.21-0.69)	184.92 (103.6-299.34)	0.4 (0.23-0.65)	-0.2 (-0.25--0.15)
Cabo Verde	2.04 (1.15-3.21)	0.38 (0.21-0.59)	1.58 (0.76-2.53)	0.33 (0.16-0.54)	-0.42 (-0.49--0.35)
Chad	68.77 (40.97-108.72)	0.6 (0.35-0.94)	159.96 (90.1-250.37)	0.47 (0.27-0.75)	-0.95 (-1.05--0.85)
Côte d’Ivoire	76.73 (36.56-129.4)	0.36 (0.17-0.6)	144.58 (75.19-239.85)	0.35 (0.18-0.58)	-0.09 (-0.17-0)
Gambia	6.44 (3.31-10.26)	0.37 (0.19-0.59)	12.85 (6.8-20.06)	0.37 (0.2-0.58)	0.04 (0.01-0.07)
Ghana	108.87 (59.54-175.05)	0.44 (0.25-0.71)	176.74 (91.66-290.68)	0.4 (0.21-0.65)	-0.3 (-0.34--0.25)
Guinea	3.01 (1.47-5.06)	0.39 (0.2-0.65)	3.08 (0.9-5.98)	0.17 (0.05-0.33)	-4.15 (-4.81--3.48)
Guinea-Bissau	8.34 (4.7-13.46)	0.47 (0.27-0.76)	14.12 (7.36-22.4)	0.45 (0.23-0.7)	-0.19 (-0.21--0.17)
Liberia	21.05 (12.37-35.11)	0.49 (0.29-0.81)	32.84 (19.29-53.2)	0.45 (0.26-0.73)	-0.9 (-1.12--0.69)
Mali	91.3 (54.13-147.66)	0.57 (0.33-0.91)	178.87 (91.39-288.17)	0.42 (0.22-0.67)	-1.14 (-1.22--1.05)
Mauritania	11.06 (4.98-17.98)	0.31 (0.14-0.51)	18.2 (8.3-29.47)	0.29 (0.13-0.47)	-0.27 (-0.3--0.25)
Niger	95.12 (55.75-150.35)	0.6 (0.35-0.94)	240.54 (146.48-382.35)	0.5 (0.31-0.79)	-0.75 (-0.89--0.61)
Nigeria	653.11 (375.82-1031.14)	0.44 (0.25-0.69)	1266.14 (662.83-2061.5)	0.37 (0.2-0.59)	-0.97 (-1.13--0.81)
São Tomé and Príncipe	0.73 (0.37-1.18)	0.38 (0.19-0.62)	0.9 (0.45-1.46)	0.37 (0.18-0.6)	-0.18 (-0.25--0.12)
Senegal	53.17 (27.14-83.56)	0.39 (0.2-0.6)	82.55 (42.81-139.33)	0.38 (0.2-0.64)	-0.17 (-0.21--0.14)
Sierra Leone	36.56 (20.15-60.35)	0.5 (0.28-0.83)	59.03 (34.27-94.38)	0.46 (0.27-0.73)	-0.29 (-0.48--0.1)
Togo	28.16 (16.29-44.55)	0.44 (0.25-0.7)	48.52 (27.02-75.13)	0.43 (0.24-0.67)	-0.04 (-0.09-0.02)
**Eastern sub-Saharan Africa**					
Burundi	46.94 (26.5-75.66)	0.47 (0.27-0.75)	97.63 (57.47-160.21)	0.48 (0.28-0.78)	-0.08 (-0.2-0.04)
Comoros	2.48 (1.27-4.04)	0.32 (0.16-0.52)	2.66 (1.42-4.12)	0.33 (0.18-0.51)	0.03 (-0.01-0.06)
Djibouti	1.88 (0.93-3.08)	0.3 (0.15-0.49)	4.25 (2.1-6.92)	0.3 (0.15-0.48)	-0.08 (-0.17-0.02)

Eritrea	25.07 (14.54-41.01)	0.44 (0.25-0.7)	33.93 (19.25-53.6)	0.39 (0.22-0.61)	-0.23 (-0.35--0.11)
Ethiopia	572.96 (350.63-893.87)	0.63 (0.39-0.97)	589.27 (333.58-932.53)	0.38 (0.22-0.6)	-1.99 (-2.19--1.79)
Kenya	156.56 (88.8-241.96)	0.41 (0.24-0.62)	223.17 (125.01-346.12)	0.39 (0.22-0.6)	-0.18 (-0.2--0.16)
Madagascar	80.04 (46.06-131.35)	0.4 (0.23-0.65)	147.61 (87.08-228.49)	0.38 (0.22-0.59)	-0.29 (-0.33--0.24)
Malawi	80.82 (47.63-130.25)	0.46 (0.27-0.73)	104.63 (61.01-166.74)	0.4 (0.24-0.64)	-0.62 (-0.68--0.55)
Mozambique	127.16 (78.27-198.21)	0.57 (0.35-0.87)	188.12 (108.14-303.29)	0.39 (0.22-0.62)	-1.54 (-1.63--1.44)
Rwanda	59.06 (35.64-94.13)	0.49 (0.3-0.76)	66.16 (37.87-103.53)	0.4 (0.23-0.62)	-1.08 (-1.26--0.9)
Somalia	104.32 (64.99-163.09)	0.71 (0.45-1.11)	298.01 (181.93-457.18)	0.76 (0.47-1.16)	0.11 (0.01-0.22)
South Sudan	29.29 (13.42-49.73)	0.3 (0.14-0.5)	54.96 (30.16-88.39)	0.36 (0.2-0.58)	0.46 (0.32-0.59)
United Republic of Tanzania	172.62 (95.03-273.81)	0.39 (0.22-0.62)	278.91 (148.83-453.01)	0.34 (0.18-0.55)	-0.72 (-0.81--0.63)
Uganda	148.74 (87.15-237.69)	0.46 (0.27-0.72)	257.43 (144.05-402.3)	0.38 (0.22-0.6)	-0.75 (-0.81--0.68)
Zambia	53.55 (29.32-84.85)	0.38 (0.21-0.6)	92.96 (46.87-152.72)	0.33 (0.17-0.55)	-0.68 (-0.83--0.53)
**Central sub-Saharan Africa**					
Angola	73.35 (35.8-124.84)	0.39 (0.2-0.66)	173.92 (83.38-311.32)	0.33 (0.16-0.58)	-0.95 (-1.05--0.84)
Central African Republic	27.42 (15.15-43.97)	0.58 (0.31-0.91)	47.34 (27.84-76.16)	0.59 (0.35-0.94)	0.06 (0.01-0.11)
Congo	12.56 (6.06-21.92)	0.33 (0.16-0.58)	21.98 (11.27-36.62)	0.36 (0.18-0.59)	0.1 (-0.01-0.21)
Democratic Republic of the Congo	305.7 (166.86-511.07)	0.45 (0.25-0.74)	632.73 (363.42-1057.09)	0.49 (0.29-0.82)	0.13 (-0.23-0.49)
Equatorial Guinea	3.01 (1.47-5.06)	0.39 (0.2-0.65)	3.08 (0.9-5.98)	0.17 (0.05-0.33)	-4.15 (-4.81--3.48)
Gabon	3.01 (1.02-5.75)	0.2 (0.07-0.38)	4.33 (1.63-7.93)	0.21 (0.08-0.38)	0.07 (0-0.13)

Abbreviation: DALYs, disability adjusted life years; EAPC, estimated annual percentage changes; IDD, intellectual developmental disability; CHD, congenital heart disease; ASDR, age-standardized DALYs rates; UI, uncertainty intervals; CI, confidence intervals; SDI, social demographic index.

eAppendix 4. Global burden of IDD attributable to CHD across age subgroups (STable 5)

STable 5. Data of the global prevalence and DALYs of different levels of IDD attributable to CHD across age subgroups and sexes

Age subgroups	**Subtypes of developmental intellectual disability, Prevalence, Number (thousand)**											
	Borderline		Mild		Moderate		Severe		Profound		Total	
	Male	Female	Male	Female	Male	Female	Male	Female	Male	Female	Male	Female
Neonates	12.5251	11.7603	2.6424	2.7126	0.8488	0.9333	0.2555	0.2959	0.5154	0.6043	16.7874	16.3064
Early infants	45.0735	43.3279	9.6502	9.9580	3.0549	3.4570	0.9171	1.1004	2.0681	2.4864	60.6738	60.3296
Late infants	38.9058	37.4259	8.2870	8.5851	2.6440	2.9484	0.7570	0.9391	1.9719	2.3231	52.5657	52.2218
Toddlers	69.3636	64.9850	15.3682	15.1385	4.7739	5.1167	1.4268	1.6419	4.3452	4.4096	95.2778	91.2917
Preschoolers	169.5667	147.4167	36.6742	33.9746	10.7144	10.8414	2.8520	3.3979	10.7038	9.9934	230.5112	205.6241
Schoolers	4.4056	3.8315	9.4863	8.7876	1.0526	1.1112	0.2772	0.3345	1.1084	0.9879	16.3303	15.0528
Adolescents	4.8329	4.9226	10.2697	11.1471	1.1444	1.4714	0.2301	0.3982	1.1955	1.1903	17.6727	19.1296
Adults	13.6756	16.2218	22.8477	31.5126	2.4660	4.1276	0.3961	1.0225	3.5441	3.9915	42.9296	56.8759

Age subgroups	**Subtypes of developmental intellectual disability, DALYs, Number (thousand)**											
	Borderline		Mild		Moderate		Severe		Profound		Total	
	Male	Female	Male	Female	Male	Female	Male	Female	Male	Female	Male	Female
Neonates	0.1345	0.1261	0.1108	0.1140	0.0834	0.0923	0.0408	0.0472	0.1037	0.1225	0.4732	0.5010
Early infants	0.4838	0.4645	0.4052	0.4222	0.3052	0.3447	0.1467	0.1757	0.4157	0.4994	1.7567	1.9067
Late infants	0.4177	0.4018	0.3514	0.3642	0.2626	0.2932	0.1213	0.1505	0.3962	0.4663	1.5502	1.6759
Toddlers	0.7432	0.6955	0.6529	0.6427	0.4771	0.5110	0.2288	0.2631	0.8718	0.8843	2.9739	2.9967
Preschoolers	1.8207	1.5787	1.5590	1.4437	1.0707	1.0816	0.4565	0.5438	2.1469	2.0035	7.0539	6.6514
Schoolers	0.0476	0.0413	0.4038	0.3739	0.1054	0.1111	0.0443	0.0535	0.2225	0.1982	0.8236	0.7780
Adolescents	0.0522	0.0531	0.4367	0.4740	0.1145	0.1470	0.0367	0.0636	0.2398	0.0367	0.8800	0.9765
Adults	0.1481	0.1754	0.9720	1.3402	0.2466	0.4124	0.0632	0.1634	0.7107	0.8000	2.1407	2.8915

Age subgroups	**Subtypes of developmental intellectual disability, Prevalence, Rate (per 100,000)**											
	Borderline		Mild		Moderate		Severe		Profound		Total	
	Male	Female	Male	Female	Male	Female	Male	Female	Male	Female	Male	Female
Neonates	248.6917	249.7500	52.4665	57.6061	16.8544	19.8215	5.0735	6.2845	10.2347	12.8340	333.3208	346.2961
Early infants	162.2933	166.7473	34.4226	38.3232	10.9999	13.3039	3.3021	4.2351	7.4466	9.5691	218.4642	232.1787
Late infants	119.1616	122.5365	25.3817	28.1087	8.0980	9.6535	2.3185	3.0748	6.0397	7.6062	160.9995	170.9798
Toddlers	104.5997	104.6503	23.1751	24.3786	7.1989	8.2399	2.1516	2.6441	6.5525	7.1011	143.6779	147.0140
Preschoolers	81.4414	75.6528	17.6143	17.4354	5.1460	5.5637	1.3698	1.7438	5.1410	5.1285	110.7126	105.5243
Schoolers	1.2428	1.1521	2.6762	2.6423	0.3341	0.3127	0.0782	0.1106	0.3127	0.2970	4.6069	4.5261
Adolescents	0.7278	0.7856	1.5465	1.7790	0.1723	0.2348	0.0346	0.0635	0.1800	0.1900	2.6614	3.0530
Adults	0.5258	0.6111	0.8784	1.8708	0.0948	0.1554	0.0152	0.0385	0.1363	0.1504	1.6506	2.1425

Age subgroups	**Subtypes of developmental intellectual disability, DALYs, Rate (per 100,000)**											
	Borderline		Mild		Moderate		Severe		Profound		Total	

	Male	Female	Male	Female	Male	Female	Male	Female	Male	Female	Male	Female
Neonates	2.6711	2.6780	2.2003	2.4204	1.6567	1.9597	0.8098	1.0002	2.0584	2.5800	9.3962	10.6404
Early infants	1.7422	1.7878	1.4589	1.6249	1.0989	1.3266	0.5284	0.6764	1.4969	1.9221	6.3253	7.3378
Late infants	1.2793	1.3155	1.0762	1.1923	0.8075	0.9598	0.3715	0.4928	1.2133	1.5267	4.7478	5.4872
Toddlers	1.1208	1.1201	0.9846	1.0350	0.7194	0.8229	0.3450	0.4237	1.3146	1.4240	4.4845	4.8257
Preschoolers	0.8745	0.8102	0.7488	0.7409	0.5142	0.5551	0.2193	0.2791	1.0311	1.0282	3.3879	3.4134
Schoolers	0.0134	0.0124	0.1139	0.1124	0.0297	0.0334	0.0125	0.0161	0.0638	0.0596	0.2323	0.2339
Adolescents	0.0079	0.0085	0.0658	0.0756	0.0172	0.0235	0.0055	0.0101	0.0361	0.0381	0.1325	0.1558
Adults	0.0057	0.0066	0.0374	0.0505	0.0095	0.0155	0.0024	0.0062	0.0273	0.0301	0.0823	0.1089

Abbreviation: DALYs, disability adjusted life years; IDD, intellectual developmental disability; CHD, congenital heart disease.

eAppendix 5. Temporal trends of DID attributable to CHD in joinpoint models (SFigure 1 to 8; STable 6 and STable 7)

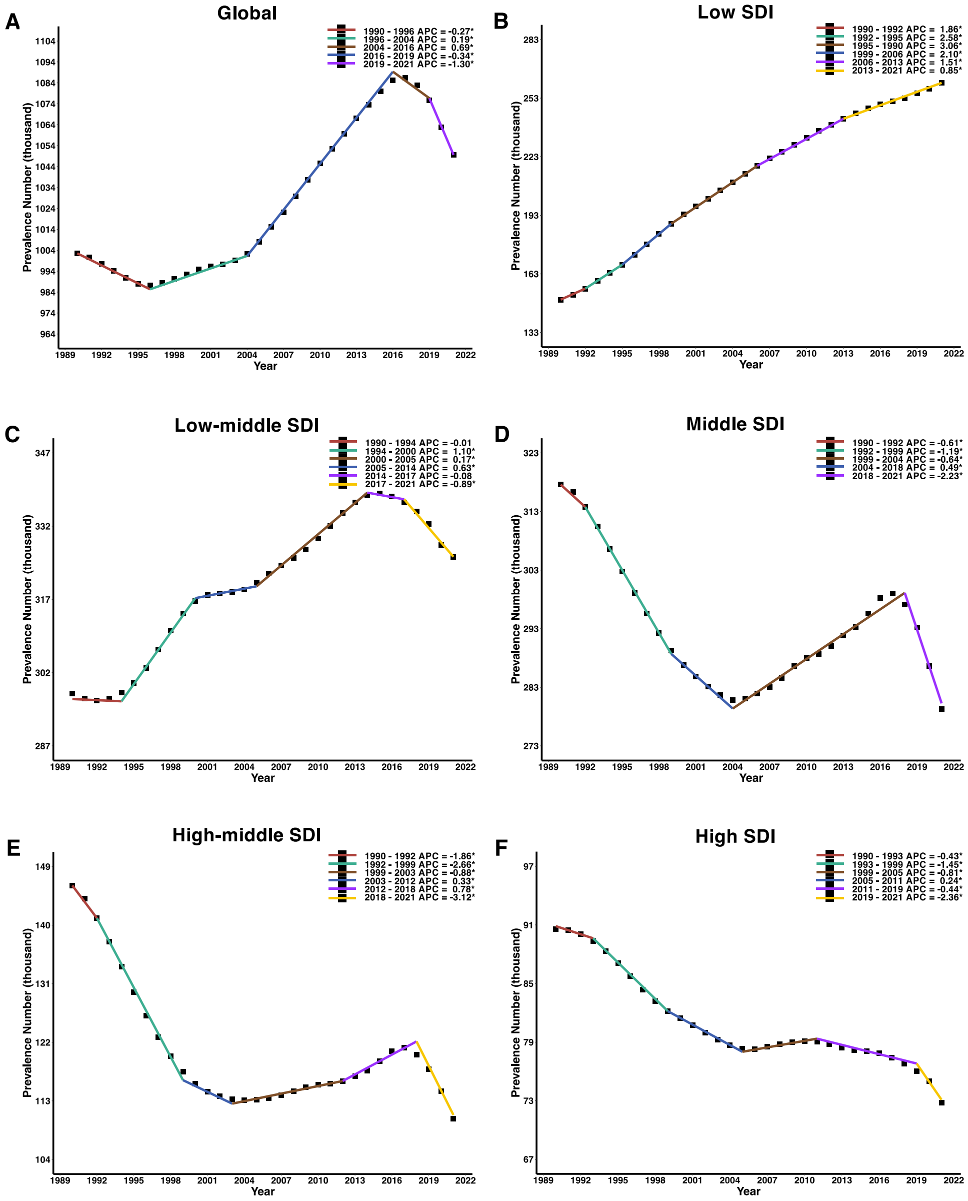

SFigure 1. Temporal trends and the APC of prevalence in number of IDD attributable to CHD across global and five SDI regions from 1990 to 2021. (A) Global; (B) Low SDI regions; (C) Low-middle SDI regions; (D) Middle SDI regions; (E) High-middle SDI regions; (F) High SDI regions.

Abbreviations: APC, annual percentage changes; IDD, intellectual developmental disability; CHD, congenital heart disease; SDI, social demographic index.

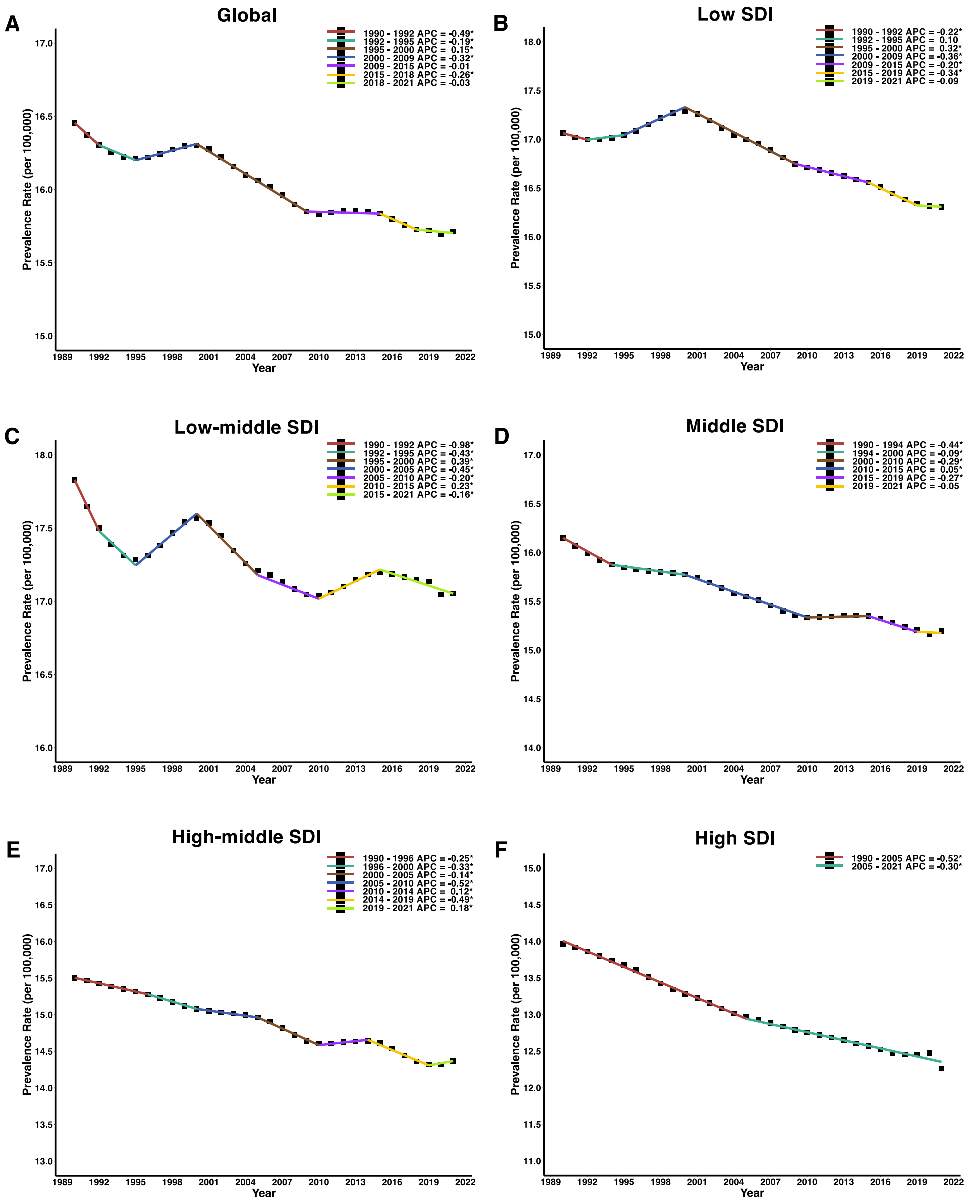

SFigure 2. Temporal trends and the APC of prevalence in rate of IDD attributable to CHD across global and five SDI regions from 1990 to 2021. (A) Global; (B) Low SDI regions; (C) Low-middle SDI regions; (D) Middle SDI regions; (E) High-middle SDI regions; (F) High SDI regions.

Abbreviations: APC, annual percentage changes; IDD, intellectual developmental disability; CHD, congenital heart disease; SDI, social demographic index.

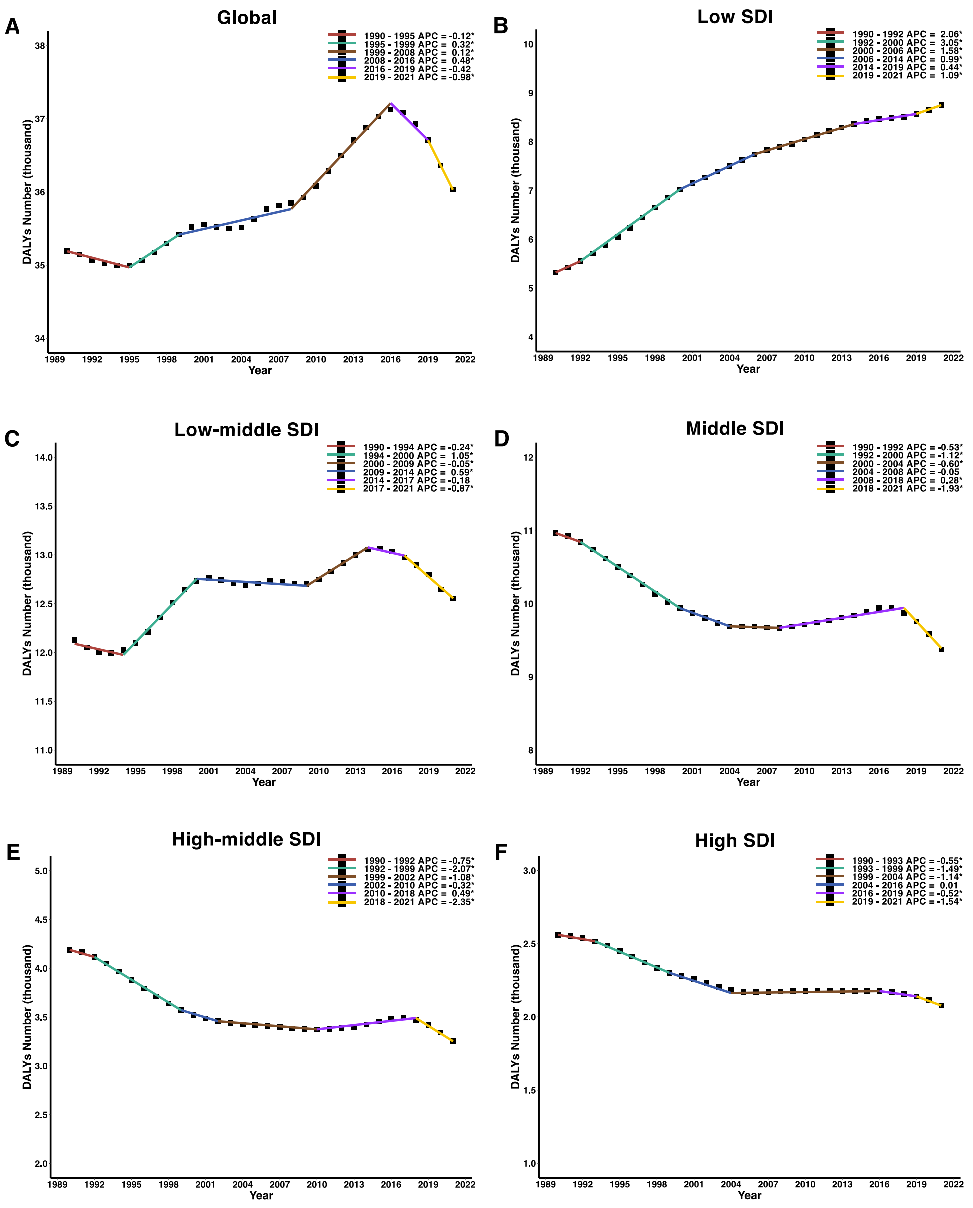

SFigure 3. Temporal trends and the APC of DALY in number of IDD attributable to CHD across global and five SDI regions from 1990 to 2021. (A) Global; (B) Low SDI regions; (C) Low-middle SDI regions; (D) Middle SDI regions; (E) High-middle SDI regions; (F) High SDI regions.

Abbreviations: APC, annual percentage changes; DALY, disability-adjusted life years; IDD, intellectual developmental disability; CHD, congenital heart disease; SDI, social demographic index.

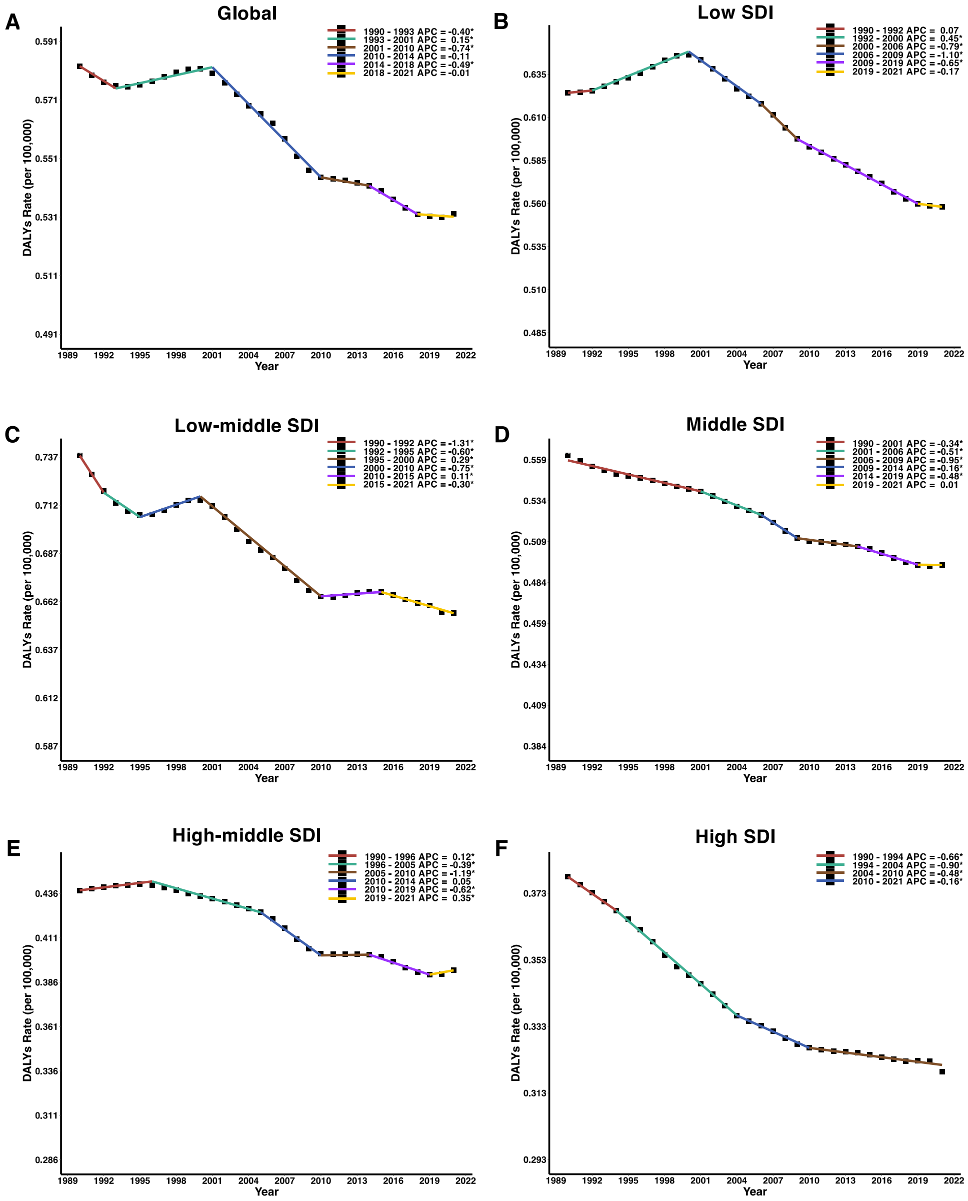

SFigure 4. Temporal trends and the APC of DALY in rate of IDD attributable to CHD across global and five SDI regions from 1990 to 2021. (A) Global; (B) Low SDI regions; (C) Low-middle SDI regions; (D) Middle SDI regions; (E) High-middle SDI regions; (F) High SDI regions.

Abbreviations: APC, annual percentage changes; DALY, disability-adjusted life years; IDD, intellectual developmental disability; CHD, congenital heart disease; SDI, social demographic index.

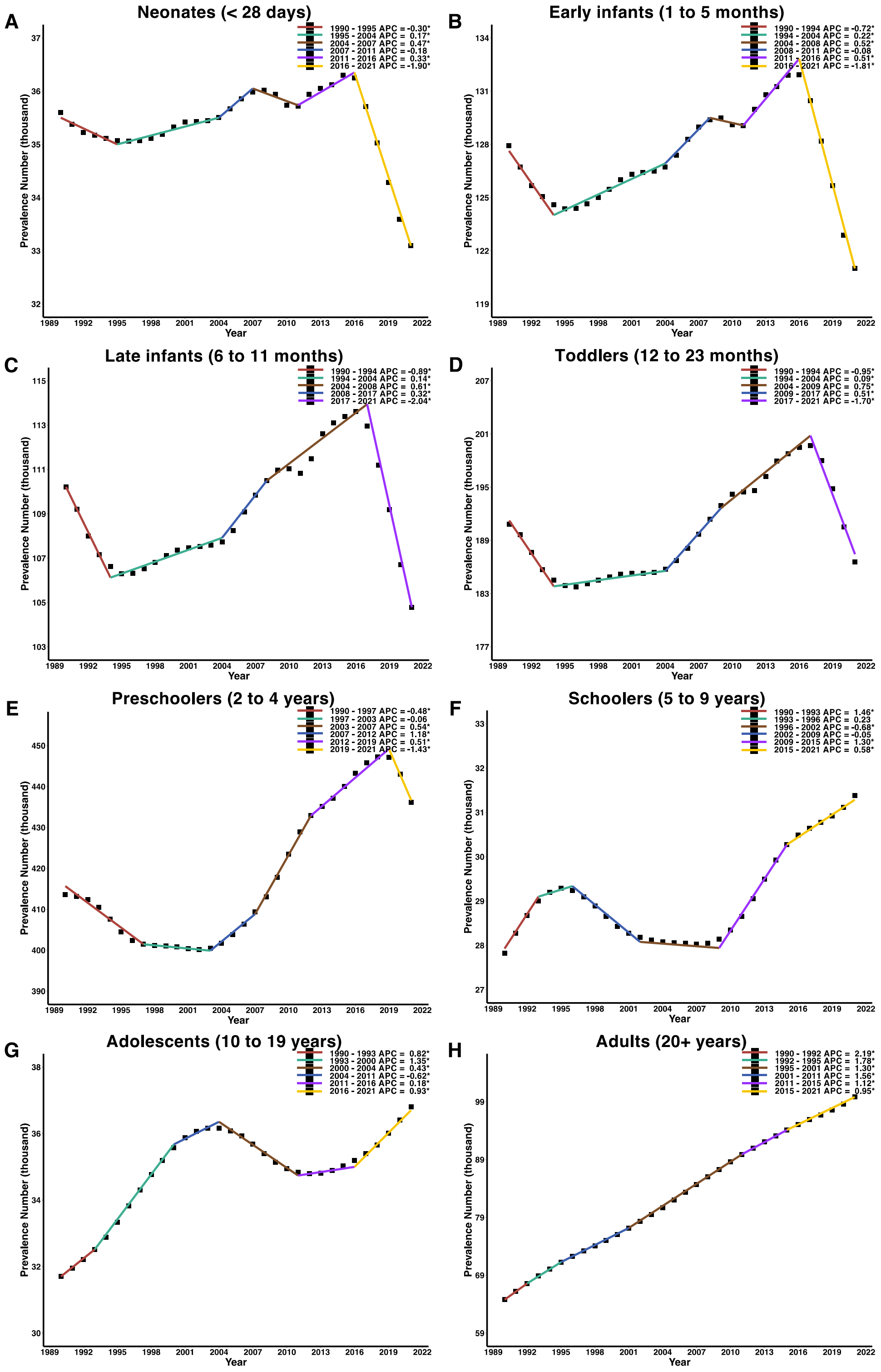

SFigure 5. Temporal trends and the APC of prevalence in number of IDD attributable to CHD across eight age subgroups from 1990 to 2021. (A) Neonates (< 28 days); (B) Early infants (1 to 5 months); (C) Late infants (6 to 11 months); (D) Toddlers (12 to 23 months); (E) Preschoolers (2 to 4 years); (F) Schoolers (5 to 9 years); (G) Adolescents (10 to 19 years); Adults (20+ years).

Abbreviations: APC, annual percentage changes; IDD, intellectual developmental disability; CHD, congenital heart disease.

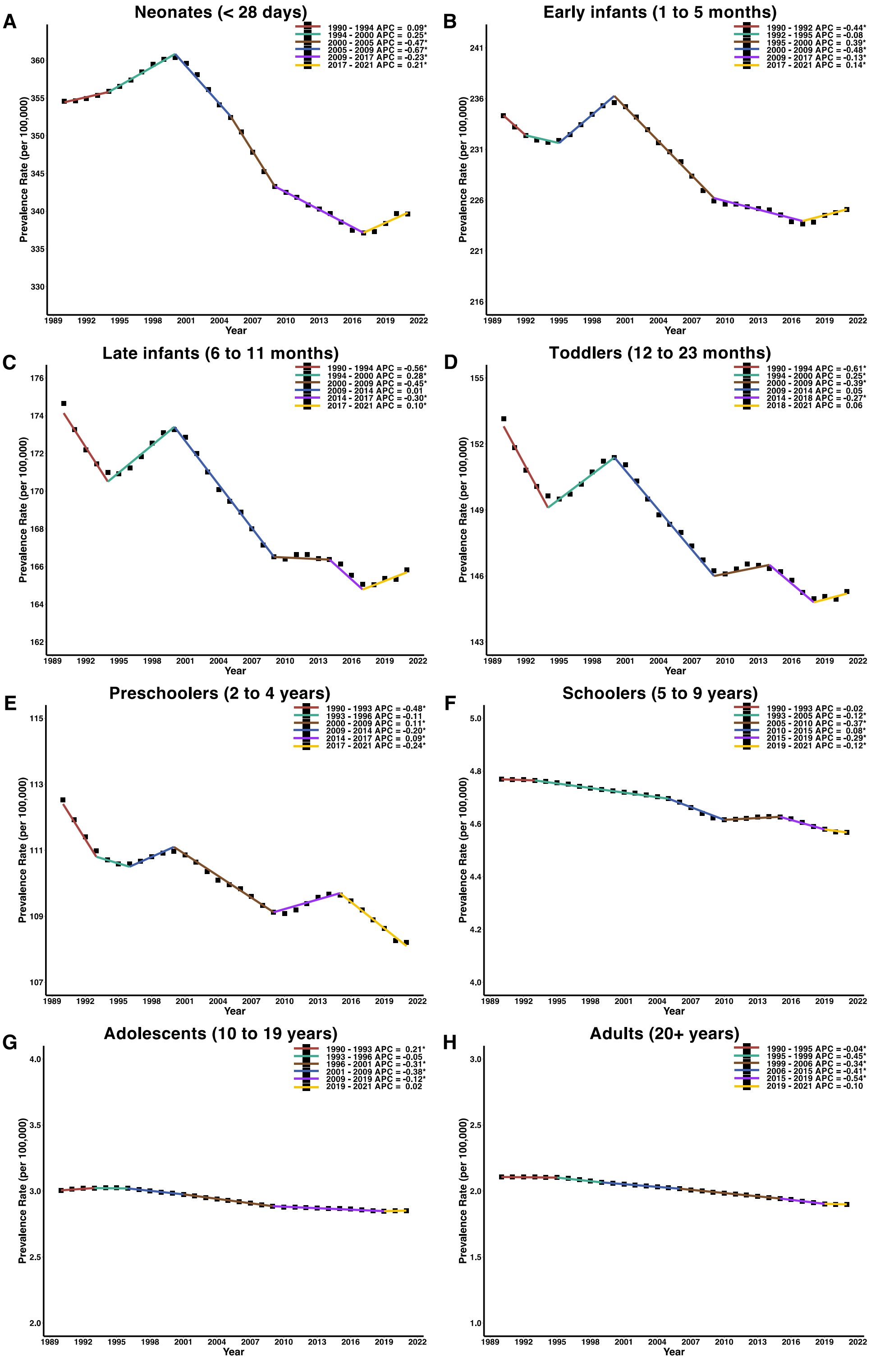

SFigure 6. Temporal trends and the APC of prevalence in rate of IDD attributable to CHD across eight age subgroups from 1990 to 2021. (A) Neonates (< 28 days); (B) Early infants (1 to 5 months); (C) Late infants (6 to 11 months); (D) Toddlers (12 to 23 months); (E) Preschoolers (2 to 4 years); (F) Schoolers (5 to 9 years); (G) Adolescents (10 to 19 years); Adults (20+ years).

Abbreviations: APC, annual percentage changes; IDD, intellectual developmental disability; CHD, congenital heart disease.

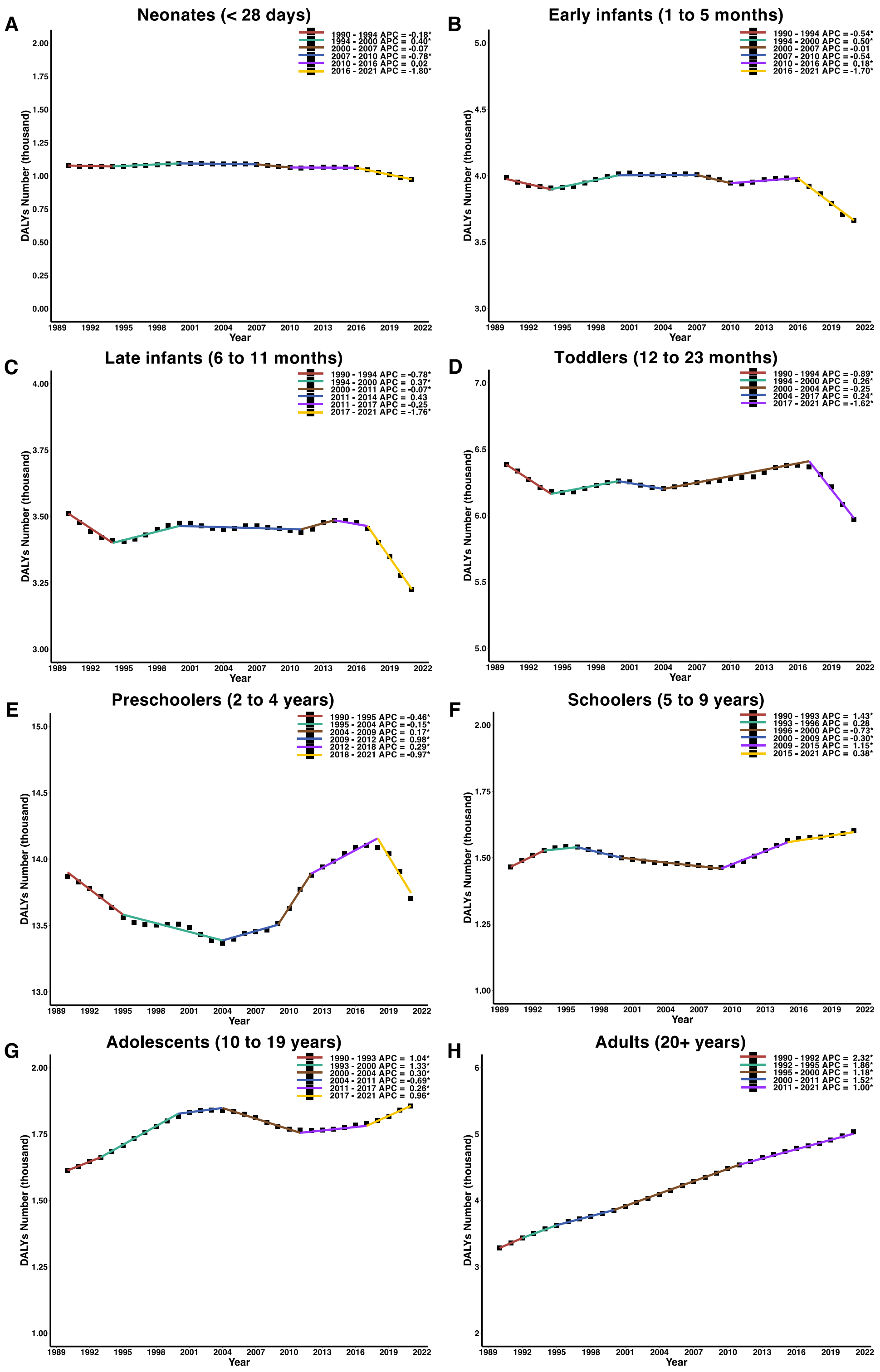

SFigure 7. Temporal trends and the APC of DALY in number of IDD attributable to CHD across eight age subgroups from 1990 to 2021. (A) Neonates (< 28 days); (B) Early infants (1 to 5 months); (C) Late infants (6 to 11 months); (D) Toddlers (12 to 23 months); (E) Preschoolers (2 to 4 years); (F) Schoolers (5 to 9 years); (G) Adolescents (10 to 19 years); Adults (20+ years).

Abbreviations: APC, annual percentage changes; DALY, disability-adjusted life years; IDD, intellectual developmental disability; CHD, congenital heart disease.

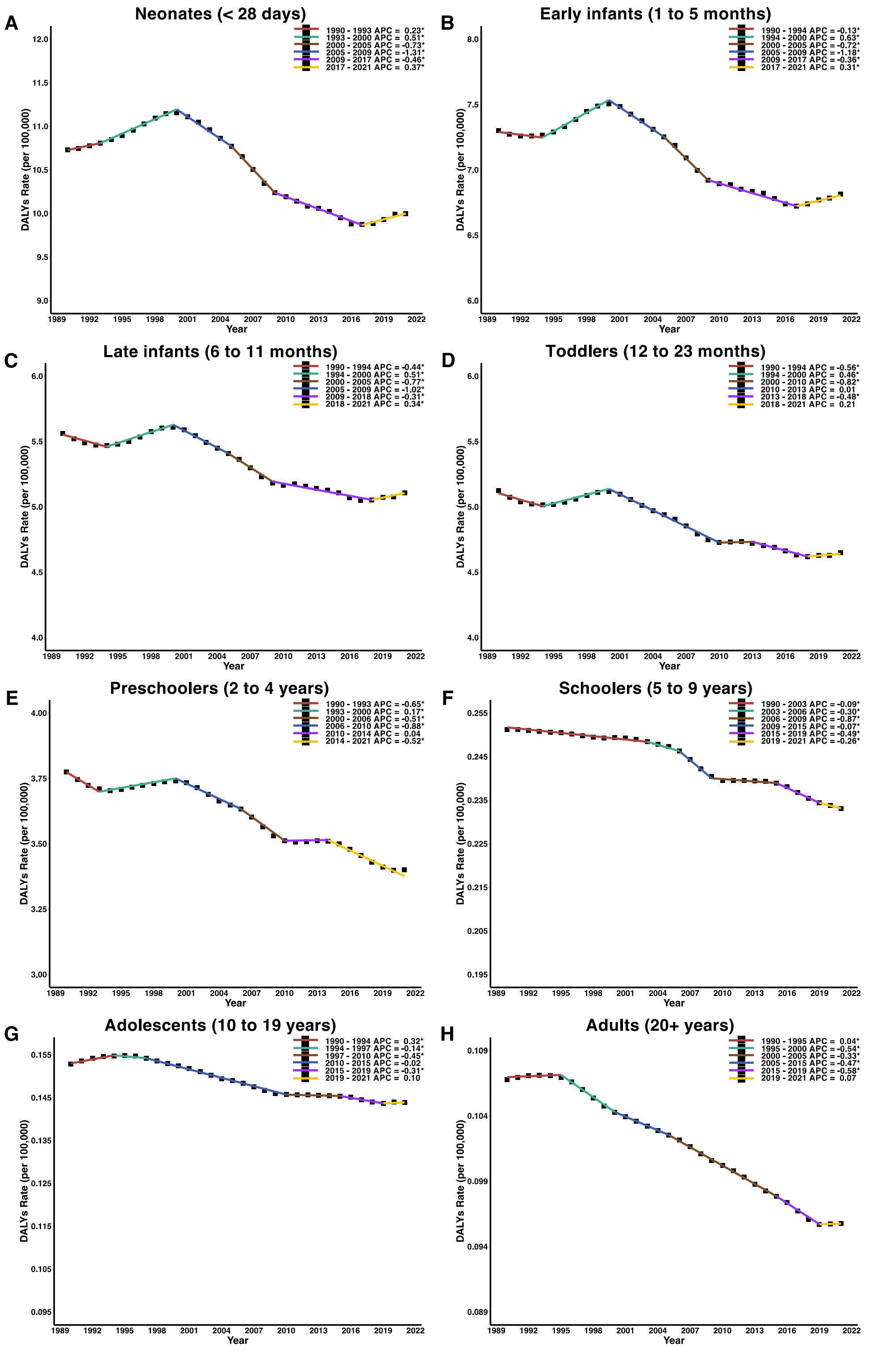

SFigure 8. Temporal trends and the APC of DALY in rate of IDD attributable to CHD across eight age subgroups from 1990 to 2021. (A) Neonates (< 28 days); (B) Early infants (1 to 5 months); (C) Late infants (6 to 11 months); (D) Toddlers (12 to 23 months); (E) Preschoolers (2 to 4 years); (F) Schoolers (5 to 9 years); (G) Adolescents (10 to 19 years); Adults (20+ years).

Abbreviations: APC, annual percentage changes; DALY, disability-adjusted life years; IDD, intellectual developmental disability; CHD, congenital heart disease.

STable 6. The APC and AAPC of IDD attributable to CHD with joinpoint regression analysis from 1990 to 2021 across global and five SDI regions

	Prevalence, Number					
	**Segment Year Start**	**Segment Year End**	**APC (95%CI)**	**P-value**	**AAPC (95%CI)**	**P-value**
Global	1990	1996	-0.2715 (-0.3046, -0.2384)	< 0.001*	0.1473 (0.1224, 0.1721)	< 0.001*
	1996	2004	0.1943 (0.1683, 0.2204)	< 0.001*		
	2004	2016	0.6942 (0.6807, 0.7077)	< 0.001*		
	2016	2019	-0.3448 (-0.5522, -0.1368)	0.003*		
	2019	2021	-1.3041 (-1.5138, -1.094)	< 0.001*		
Low SDI	1990	1992	1.8615 (1.4799, 2.2446)	< 0.001*	1.799 (1.7476, 1.8503)	< 0.001*
	1992	1995	2.5805 (2.1951, 2.9673)	< 0.001*		
	1995	1999	3.0626 (2.8695, 3.2561)	< 0.001*		
	1999	2006	2.1030 (2.0387, 2.1673)	< 0.001*		
	2006	2013	1.5141 (1.4512, 1.5771)	< 0.001*		
	2013	2021	0.8518 (0.8107, 0.8929)	< 0.001*		
Low-middle SDI	1990	1994	-0.0097 (-0.1505, 0.1313)	0.886	0.2985 (0.2449, 0.3521)	< 0.001*
	1994	2000	1.1046 (1.0062, 1.2032)	< 0.001*		
	2000	2005	0.1698 (0.0318, 0.3081)	0.019*		
	2005	2014	0.6326 (0.5854, 0.6799)	< 0.001*		
	2014	2017	-0.0838 (-0.5089, 0.3430)	0.681		
	2017	2021	-0.8926 (-1.0264, -0.7585)	< 0.001*		
Middle SDI	1990	1992	-0.6120 (-1.092, -0.1297)	0.016*	-0.4108 (-0.4590, -0.3627)	< 0.001*
	1992	1999	-1.1857 (-1.2652, -1.1062)	< 0.001*		
	1999	2004	-0.6435 (-0.794, -0.4928)	< 0.001*		
	2004	2018	0.4875 (0.4611, 0.5138)	< 0.001*		
	2018	2021	-2.2297 (-2.4849, -1.9738)	< 0.001*		
High-middle SDI	1990	1992	-1.8593 (-2.5102, -1.204)	< 0.001*	-0.9016 (-0.9764, -0.8266)	< 0.001*
	1992	1999	-2.6560 (-2.7554, -2.5566)	< 0.001*		
	1999	2003	-0.8796 (-1.1715, -0.5868)	< 0.001*		
	2003	2012	0.3270 (0.2525, 0.4016)	< 0.001*		
	2012	2018	0.7752 (0.6051, 0.9456)	< 0.001*		
	2018	2021	-3.1221 (-3.4953, -2.7474)	< 0.001*		
High SDI	1990	1993	-0.4299 (-0.6268, -0.2326)	< 0.001*	-0.7009 (-0.7451, -0.6567)	< 0.001*
	1993	1999	-1.4475 (-1.5364, -1.3585)	< 0.001*		
	1999	2005	-0.8103 (-0.9038, -0.7168)	< 0.001*		
	2005	2011	0.2355 (0.1409, 0.3301)	< 0.001*		
	2011	2019	-0.4382 (-0.4950, -0.3814)	< 0.001*		
	2019	2021	-2.3619 (-2.7851, -1.9370)	< 0.001*		
	**Prevalence, Rate**					
	**Segment Year Start**	**Segment Year End**	**APC (95%CI)**	**P-value**	**AAPC (95%CI)**	**P-value**
Global	1990	1992	-0.4945 (-0.6523, -0.3365)	< 0.001*	-0.1503 (-0.1757, -0.1248)	< 0.001*
	1992	1995	-0.1921 (-0.3486, -0.0353)	0.020*		
	1995	2000	0.1472 (0.0975, 0.1970)	< 0.001*		
	2000	2009	-0.3220 (-0.3391, -0.3048)	< 0.001*		
	2009	2015	-0.0099 (-0.0461, 0.0263)	0.562		

	Segment Year Start	Segment Year End	APC (95%CI)	P-value	AAPC (95%CI)	P-value
	2015	2018	-0.2559 (-0.4224, -0.0893)	0.006*		
	2018	2021	-0.0328 (-0.1185, 0.0530)	0.421		
Low SDI	1990	1992	-0.2159 (-0.373, -0.0585)	0.011*	-0.1457 (-0.1692, -0.1222)	< 0.001*
	1992	1995	0.1043 (-0.0543, 0.2631)	0.177		
	1995	2000	0.3180 (0.2680, 0.3680)	< 0.001*		
	2000	2009	-0.3649 (-0.3817, -0.3480)	< 0.001*		
	2009	2015	-0.1952 (-0.2299, -0.1604)	< 0.001*		
	2015	2019	-0.3368 (-0.4162, -0.2574)	< 0.001*		
	2019	2021	-0.0887 (-0.2484, 0.0713)	0.250		
Low-middle SDI	1990	1992	-0.9793 (-1.2281, -0.7298)	< 0.001*	-0.1437 (-0.1786, -0.1087)	< 0.001*
	1992	1995	-0.4257 (-0.6698, -0.181)	0.003*		
	1995	2000	0.3875 (0.3093, 0.4657)	< 0.001*		
	2000	2005	-0.4522 (-0.5297, -0.3747)	< 0.001*		
	2005	2010	-0.2049 (-0.2821, -0.1277)	< 0.001*		
	2010	2015	0.2252 (0.1480, 0.3024)	< 0.001*		
	2015	2021	-0.1624 (-0.2036, -0.1212)	< 0.001*		
Middle SDI	1990	1994	-0.4403 (-0.4762, -0.4044)	< 0.001*	-0.1974 (-0.2113, -0.1836)	< 0.001*
	1994	2000	-0.0895 (-0.1149, -0.0641)	< 0.001*		
	2000	2010	-0.2893 (-0.2999, -0.2788)	< 0.001*		
	2010	2015	0.0481 (0.0100, 0.0862)	0.017*		
	2015	2019	-0.2666 (-0.3291, -0.2041)	< 0.001*		
	2019	2021	-0.0493 (-0.1761, 0.0776)	0.420		
High-middle SDI	1990	1996	-0.2458 (-0.2571, -0.2346)	< 0.001*	-0.2472 (-0.2570, -0.2374)	< 0.001*
	1996	2000	-0.3337 (-0.3649, -0.3026)	< 0.001*		
	2000	2005	-0.1366 (-0.1570, -0.1161)	< 0.001*		
	2005	2010	-0.5174 (-0.5405, -0.4943)	< 0.001*		
	2010	2014	0.1240 (0.0839, 0.1641)	< 0.001*		
	2014	2019	-0.4853 (-0.5111, -0.4594)	< 0.001*		
	2019	2021	0.1781 (0.0938, 0.2624)	< 0.001*		
High SDI	1990	2005	-0.5154 (-0.5418, -0.489)	< 0.001*	-0.4056 (-0.423, -0.3882)	< 0.001*
	2005	2021	-0.3026 (-0.3278, -0.2774)	< 0.001*		
	**DALYs, Number**					
	**Segment Year Start**	**Segment Year End**	**APC (95%CI)**	**P-value**	**AAPC (95%CI)**	**P-value**
Global	1990	1995	-0.1240 (-0.2231, -0.0247)	0.018*	0.0766 (0.0174, 0.1357)	0.011*
	1995	1999	0.3224 (0.1027, 0.5426)	0.007*		
	1999	2008	0.1174 (0.0697, 0.1652)	< 0.001*		
	2008	2016	0.4850 (0.4259, 0.5441)	< 0.001*		
	2016	2019	-0.4195 (-0.8593, 0.0223)	0.061		
	2019	2021	-0.9756 (-1.4130, -0.5361)	< 0.001*		
Low SDI	1990	1992	2.0613 (1.6447, 2.4796)	< 0.001*	1.6191 (1.5729, 1.6653)	< 0.001*
	1992	2000	3.0519 (2.9954, 3.1085)	< 0.001*		
	2000	2006	1.5827 (1.4916, 1.6739)	< 0.001*		
	2006	2014	0.9872 (0.9336, 1.0409)	< 0.001*		
	2014	2019	0.4428 (0.3177, 0.5680)	< 0.001*		

	2019	2021	1.0905 (0.6915, 1.4911)	< 0.001*		
Low-middle SDI	1990	1994	-0.2382 (-0.3836, -0.0925)	0.003*	0.1204 (0.0643, 0.1765)	< 0.001*
	1994	2000	1.0451 (0.9426, 1.1477)	< 0.001*		
	2000	2009	-0.0526 (-0.1022, -0.003)	0.039*		
	2009	2014	0.5918 (0.4474, 0.7364)	< 0.001*		
	2014	2017	-0.1832 (-0.6307, 0.2664)	0.398		
	2017	2021	-0.8664 (-1.0069, -0.7257)	< 0.001*		
Middle SDI	1990	1992	-0.5345 (-0.8419, -0.2262)	0.002*	-0.5033 (-0.5395, -0.4672)	< 0.001*
	1992	2000	-1.116 (-1.1564, -1.0756)	< 0.001*		
	2000	2004	-0.5954 (-0.7453, -0.4453)	< 0.001*		
	2004	2008	-0.0479 (-0.1974, 0.1019)	0.506		
	2008	2018	0.2836 (0.2560, 0.3113)	< 0.001*		
	2018	2021	-1.9295 (-2.0793, -1.7794)	< 0.001*		
High-middle SDI	1990	1992	-0.7520 (-1.2840, -0.2171)	< 0.001*	-0.8094 (-0.8768, -0.742)	< 0.001*
	1992	1999	-2.0746 (-2.1611, -1.9879)	< 0.001*		
	1999	2002	-1.0775 (-1.5883, -0.5642)	< 0.001*		
	2002	2010	-0.3196 (-0.3893, -0.2498)	< 0.001*		
	2010	2018	0.4923 (0.4208, 0.5640)	< 0.001*		
	2018	2021	-2.3479 (-2.6120, -2.0831)	< 0.001*		
High SDI	1990	1993	-0.5549 (-0.7000, -0.4096)	< 0.001*	-0.6754 (-0.7152, -0.6356)	< 0.001*
	1993	1999	-1.4943 (-1.5595, -1.4290)	< 0.001*		
	1999	2004	-1.1364 (-1.2306, -1.0422)	< 0.001*		
	2004	2016	0.0069 (-0.0134, 0.0272)	0.479		
	2016	2019	-0.5250 (-0.8231, -0.2260)	0.002*		
	2019	2021	-1.5360 (-1.8374, -1.2337)	< 0.001*		

	DALYs, Rate					
	**Segment Year Start**	**Segment Year End**	**APC (95%CI)**	**P-value**	**AAPC (95%CI)**	**P-value**
Global	1990	1993	-0.3978 (-0.6456, -0.1494)	0.004*	-0.2928 (-0.3488, -0.2367)	< 0.001*
	1993	2001	0.1469 (0.0809, 0.2128)	< 0.001*		
	2001	2010	-0.7354 (-0.7885, -0.6822)	< 0.001*		
	2010	2014	-0.1100 (-0.3560, 0.1367)	0.357		
	2014	2028	-0.4905 (-0.7373, -0.2430)	< 0.001*		
	2018	2021	-0.0061 (-0.2554, 0.2437)	0.959		
Low SDI	1990	1992	0.0732 (-0.2143, 0.3616)	0.596	-0.3609 (-0.3989, -0.3230)	< 0.001*
	1992	2000	0.4472 (0.4091, 0.4853)	< 0.001*		
	2000	2006	-0.7913 (-0.8529, -0.7297)	< 0.001*		
	2006	2009	-1.0995 (-1.3779, -0.8203)	< 0.001*		
	2009	2019	-0.6486 (-0.6739, -0.6234)	< 0.001*		
	2019	2021	-0.1670 (-0.4439, 0.1107)	0.219		
Low-middle SDI	1990	1992	-1.3109 (-1.5994, -1.0215)	< 0.001*	-0.3807 (-0.4186, -0.3427)	< 0.001*
	1992	1995	-0.5982 (-0.8834, -0.3123)	< 0.001*		
	1995	2000	0.2879 (0.1969, 0.3789)	< 0.001*		
	2000	2010	-0.7549 (-0.781, 0.7288)	< 0.001*		
	2010	2015	0.1139 (0.0235, 0.2044)	0.017*		

	2015	2021	-0.3017 (-0.3494, -0.2539)	< 0.001*		
Middle SDI	1990	2001	-0.3367 (-0.3692, -0.3042)	< 0.001*	-0.3970 (-0.4617, -0.3323)	< 0.001*
	2001	2006	-0.5095 (-0.6591, -0.3597)	< 0.001*		
	2006	2009	-0.9473 (-1.4155, -0.4770)	< 0.001*		
	2009	2014	-0.1639 (-0.3148, -0.0128)	0.035*		
	2014	2019	-0.4806 (-0.6311, -0.3298)	< 0.001*		
	2019	2021	0.0084 (-0.4742, 0.4935)	0.971		
High-middle SDI	1990	1996	0.1180 (0.0906, 0.1453)	< 0.001*	-0.3556 (-0.3745, -0.3368)	< 0.001*
	1996	2005	-0.3940 (-0.4114, -0.3766)	< 0.001*		
	2005	2010	-1.1874 (-1.2382, -1.1366)	< 0.001*		
	2010	2014	0.0513 (-0.0310, 0.1337)	0.204		
	2014	2019	-0.6226 (-0.6750, -0.5702)	< 0.001*		
	2019	2021	0.3465 (0.1788, 0.5145)	< 0.001*		
High SDI	1990	1994	-0.6612 (-0.8085, -0.5138)	< 0.001*	-0.5251 (-0.5568, -0.4933)	< 0.001*
	1994	2004	-0.9033 (-0.9466, -0.8600)	< 0.001*		
	2004	2010	-0.4775 (-0.5858, -0.3691)	< 0.001*		
	2010	2021	-0.1563 (-0.1891, -0.1234)	< 0.001*		

Abbreviation: APC, annual percentage changes; AAPC, average annual percentage changes; IDD, intellectual developmental disability; CHD, congenital heart disease; SDI, social demographic index; CI, confidence intervals; DALYs, disability adjusted life years.

* Statistical significance

STable 7. The APC and AAPC of IDD attributable to CHD with joinpoint regression analysis from 1990 to 2021 across eight age subgroups

	Prevalence, Number					
	**Segment Year Start**	**Segment Year End**	**APC (95%CI)**	**P-value**	**AAPC (95%CI)**	**P-value**
Neonates	1990	1995	-0.2954 (-0.3984, -0.1922)	< 0.001*	-0.2337 (-0.2952, -0.1731)	< 0.001*
	1995	2004	0.1669 (0.1173, 0.2166)	< 0.001*		
	2004	2007	0.4682 (0.0092, 0.9292)	0.046*		
	2007	2011	-0.1779 (-0.4141, 0.0589)	0.130		
	2011	2016	0.3275 (0.1714, 0.4838)	< 0.001*		
	2016	2021	-1.9014 (-2.0137, -1.7890)	< 0.001*		
Early infants	1990	1994	-0.7173 (-0.8531, -0.5814)	< 0.001*	-0.1744 (-0.2323, -0.1165)	< 0.001*
	1994	2004	0.2179 (0.1786, 0.2573)	< 0.001*		
	2004	2008	0.5190 (0.3026, 0.7359)	< 0.001*		
	2008	2011	-0.0757 (-0.5200, 0.3707)	0.722		
	2011	2016	0.5128 (0.3655, 0.6604)	< 0.001*		
	2016	2021	-1.8054 (-1.9126, -1.6982)	< 0.001*		
Late infants	1990	1994	-0.8948 (-1.1060, -0.6832)	< 0.001*		
	1994	2004	0.1425 (0.0816, 0.2035)	< 0.001*	-0.1639 (-0.2252, -0.1025)	< 0.001*
	2004	2008	0.6068 (0.2716, 0.9431)	0.001*		
	2008	2017	0.3233 (0.2487, 0.3979)	< 0.001*		
	2017	2021	-2.0382 (-2.2623, -1.8137)	< 0.001*		
Toddlers	1990	1994	-0.9536 (-1.1310, -0.7758)	< 0.001*	-0.0663 (-0.1127, -0.0199)	0.005*
	1994	2004	0.0863 (0.0353, 0.1373)	0.002*		
	2004	2009	0.7540 (0.5793, 0.9291)	< 0.001*		
	2009	2017	0.5053 (0.4316, 0.5791)	< 0.001*		
	2017	2021	-1.7040 (-1.8795, -1.5283)	< 0.001*		
Preschoolers	1990	1997	-0.4817 (-0.5539, -0.4094)	< 0.001*	0.1607 (0.0974, 0.2239)	< 0.001*
	1997	2003	-0.0581 (-0.1783, 0.0623)	0.320		
	2003	2007	0.5355 (0.2625, 0.8093)	< 0.001*		
	2007	2012	1.1816 (1.0031, 1.3604)	< 0.001*		
	2012	2019	0.5121 (0.4150, 0.6092)	< 0.001*		
	2019	2021	-1.4296 (-1.9972, -0.8587)	< 0.001*		
Schoolers	1990	1993	1.4571 (1.2808, 1.6338)	< 0.001*	0.3812 (0.3384, 0.424)	< 0.001*
	1993	1996	0.2253 (-0.1222, 0.5741)	0.187		
	1996	2002	-0.6767 (-0.7525, -0.6009)	< 0.001*		
	2002	2009	-0.0456 (-0.1032, 0.0121)	0.113		
	2009	2015	1.2951 (1.2170, 1.3733)	< 0.001*		
	2015	2021	0.5769 (0.5179, 0.6360)	< 0.001*		
Adolescents	1990	1993	0.8188 (0.6552, 0.9827)	< 0.001*	0.4771 (0.4425, 0.5118)	< 0.001*
	1993	2000	1.3489 (1.2924, 1.4055)	< 0.001*		
	2000	2004	0.4332 (0.2715, 0.5952)	< 0.001*		
	2004	2011	-0.6190 (-0.6730, -0.5650)	< 0.001*		
	2011	2016	0.1842 (0.0812, 0.2873)	0.002*		
	2016	2021	0.9299 (0.8558, 1.004)	< 0.001*		
Adults	1990	1992	2.1927 (1.9179, 2.4682)	< 0.001*	1.3974 (1.3601, 1.4347)	< 0.001*

	Segment Year Start	Segment Year End	APC (95%CI)	P-value	AAPC (95%CI)	P-value
	1992	1995	1.7777 (1.5029, 2.0532)	< 0.001*		
	1995	2001	1.3041 (1.2422, 1.3661)	< 0.001*		
	2001	2011	1.5633 (1.5378, 1.5887)	< 0.001*		
	2011	2015	1.1168 (0.9762, 1.2577)	< 0.001*		
	2015	2021	0.9488 (0.9003, 0.9972)	< 0.001*		
	**Prevalence, Rate**					
	**Segment Year Start**	**Segment Year End**	**APC (95%CI)**	**P-value**	**AAPC (95%CI)**	**P-value**
Neonates	1990	1994	0.0924 (0.0290, 0.1558)	0.007*	-0.1351 (-0.1565, -0.1137)	< 0.001*
	1994	2000	0.2464 (0.2023, 0.2905)	< 0.001*		
	2000	2005	-0.4691 (-0.5309, -0.4073)	< 0.001*		
	2005	2009	-0.6708 (-0.7706, -0.5709)	< 0.001*		
	2009	2017	-0.2306 (-0.2589, -0.2023)	< 0.001*		
	2017	2021	0.2138 (0.1434, 0.2843)	< 0.001*		
Early infants	1990	1992	-0.4448 (-0.6754, -0.2137)	< 0.001*	-0.1296 (-0.1600, -0.0991)	< 0.001*
	1992	1995	-0.0775 (-0.3073, 0.1529)	0.484		
	1995	2000	0.3865 (0.3135, 0.4595)	< 0.001*		
	2000	2009	-0.4804 (-0.5056, -0.4551)	< 0.001*		
	2009	2017	-0.1323 (-0.1653, -0.0993)	< 0.001*		
	2017	2021	0.1425 (0.0595, 0.2256)	0.002*		
Late infants	1990	1994	-0.5565 (-0.6436, -0.4694)	< 0.001*	-0.1651 (-0.2003, -0.1300)	< 0.001*
	1994	2000	0.2816 (0.2203, 0.3430)	< 0.001*		
	2000	2009	-0.4541 (-0.4838, -0.4245)	< 0.001*		
	2009	2014	0.0058 (-0.0807, 0.0923)	0.889		
	2014	2017	-0.3043 (-0.5920, -0.0158)	0.040*		
	2017	2021	0.1010 (0.0070, 0.1952)	0.037*		
Toddlers	1990	1994	-0.6112 (-0.7109, -0.5115)	< 0.001*	-0.1669 (-0.2004, -0.1335)	< 0.001*
	1994	2000	0.2454 (0.1752, 0.3157)	< 0.001*		
	2000	2009	-0.3929 (-0.4265, -0.3593)	< 0.001*		
	2009	2014	0.0512 (-0.0458, 0.1482)	0.278		
	2014	2018	-0.2727 (-0.4273, -0.1179)	0.002*		
	2018	2021	0.0608 (-0.0978, 0.2198)	0.427		
Preschoolers	1990	1993	-0.4780 (-0.5537, -0.4023)	< 0.001*	-0.1284 (-0.148, -0.1088)	< 0.001*
	1993	1996	-0.1083 (-0.2588, 0.0425)	0.146		
	1996	2000	0.1126 (0.0377, 0.1875)	0.006*		
	2000	2009	-0.1953 (-0.2118, -0.1788)	< 0.001*		
	2009	2015	0.0925 (0.0573, 0.1277)	< 0.001*		
	2015	2021	-0.2440 (-0.2709, -0.2171)	< 0.001*		
Schoolers	1990	1993	-0.0163 (-0.0581, 0.0256)	0.420	-0.1398 (-0.1496, -0.1299)	< 0.001*
	1993	2005	-0.1231 (-0.1286, -0.1175)	< 0.001*		
	2005	2010	-0.3659 (-0.3919, -0.3399)	< 0.001*		
	2010	2015	0.0847 (0.0585, 0.1109)	< 0.001*		
	2015	2019	-0.2885 (-0.3298, -0.2472)	< 0.001*		
	2019	2021	-0.1217 (-0.2064, -0.0370)	0.008*		
Adolescents	1990	1993	0.2059 (0.1141, 0.2978)	< 0.001*		

	1993	1996	-0.0464 (-0.2341, 0.1417)	0.606	-0.1705 (-0.1950, -0.1459)	< 0.001*
	1996	2001	-0.3076 (-0.3657, -0.2493)	< 0.001*		
	2001	2009	-0.3799 (-0.4041, -0.3558)	< 0.001*		
	2009	2019	-0.1229 (-0.1395, -0.1064)	< 0.001*		
	2019	2021	0.0244 (-0.1651, 0.2142)	0.788		
Adults	1990	1995	-0.0369 (-0.0650, -0.0087)	0.014*	-0.3373 (-0.3525, -0.3222)	< 0.001*
	1995	1999	-0.4547 (-0.5179, -0.3915)	< 0.001*		
	1999	2006	-0.3388 (-0.3601, -0.3174)	< 0.001*		
	2006	2015	-0.4145 (-0.4284, -0.4005)	< 0.001*		
	2015	2019	-0.5353 (-0.6005, -0.4700)	< 0.001*		
	2019	2021	-0.1039 (-0.2379, 0.0303)	0.120		

	**DALYs, Number**					
	**Segment Year Start**	**Segment Year End**	**APC (95%CI)**	**P-value**	**AAPC (95%CI)**	**P-value**
Neonates	1990	1994	-0.1790 (-0.3153, -0.0426)	0.014*	-0.3247 (-0.3768, -0.2725)	< 0.001*
	1994	2000	0.4031 (0.3068, 0.4994)	< 0.001*		
	2000	2007	-0.0697 (-0.1420, 0.0026)	0.058		
	2007	2010	-0.7759 (-1.2010, -0.3489)	0.002*		
	2010	2016	0.0218 (-0.0751, 0.1189)	0.638		
	2016	2021	-1.8005 (-1.8943, -1.7065)	< 0.001*		
Early infants	1990	1994	-0.5413 (-0.7129, -0.3694)	< 0.001*	-0.2691 (-0.3355, -0.2025)	< 0.001*
	1994	2000	0.4973 (0.3749, 0.6199)	< 0.001*		
	2000	2007	-0.0138 (-0.1058, 0.0784)	0.755		
	2007	2010	-0.5367 (-1.0782, 0.0077)	0.053		
	2010	2016	0.1844 (0.0610, 0.3080)	0.006*		
	2016	2021	-1.6958 (-1.8195, -1.5719)	< 0.001*		
Late infants	1990	1994	-0.7840 (-0.9569, -0.6108)	< 0.001*	0.2684 (-0.3481, -0.1887)	< 0.001*
	1994	2000	0.3652 (0.2425, 0.4880)	< 0.001*		
	2000	2011	-0.0716 (-0.1142, -0.0289)	0.003*		
	2011	2014	0.4267 (-0.1232, 0.9796)	0.119		
	2014	2017	-0.2511 (-0.8056, 0.3065)	0.352		
	2017	2021	-1.7610 (-1.9343, -1.5873)	< 0.001*		
Toddlers	1990	1994	-0.8902 (-1.0513, -0.7288)	< 0.001*	-0.2082 (-0.2558, 0.1605)	< 0.001*
	1994	2000	0.2637 (0.1490, 0.3785)	< 0.001*		
	2000	2004	-0.2495 (-0.5045, 0.0062)	0.055		
	2004	2017	0.2377 (0.2075, 0.2680)	< 0.001*		
	2017	2021	-1.6244 (-1.7838, -1.4648)	< 0.001*		
Preschoolers	1990	1995	-0.4625 (-0.5682, -0.3566)	< 0.001*	-0.0358 (-0.0932, 0.0217)	0.223
	1995	2004	-0.1532 (-0.2043, -0.1020)	< 0.001*		
	2004	2009	0.1730 (0.0278, 0.3185)	0.023*		
	2009	2012	0.9758 (0.5129, 1.4408)	< 0.001*		
	2012	2018	0.2900 (0.1886, 0.3915)	< 0.001*		
	2018	2021	-0.9707 (-1.1968, -0.7441)	< 0.001*		
Schoolers	1990	1993	1.4250 (1.2268, 1.6236)	< 0.001*	0.2778 (0.2267, 0.3288)	< 0.001*
	1993	1996	0.2760 (-0.1136, 0.6672)	0.152		

	1996	2000	-0.7303 (-0.9250, -0.5352)	< 0.001*		
	2000	2009	-0.3004 (-0.3436, -0.2572)	< 0.001*		
	2009	2015	1.1519 (1.0605, 1.2433)	< 0.001*		
	2015	2021	0.3832 (0.3131, 0.4533)	< 0.001*		
Adolescents	1990	1993	1.0395 (0.8379, 1.2415)	< 0.001*	0.4543 (0.4126, 0.496)	< 0.001*
	1993	2000	1.3257 (1.2585, 1.3930)	< 0.001*		
	2000	2004	0.2975 (0.1007, 0.4947)	0.006*		
	2004	2011	-0.6865 (-0.7536, -0.6193)	< 0.001*		
	2011	2017	0.2593 (0.1697, 0.3491)	< 0.001*		
	2017	2021	0.9571 (0.8302, 1.0841)	< 0.001*		
Adults	1990	1992	2.3161 (1.8791, 2.7549)	< 0.001*	1.3798 (1.3264, 1.4333)	< 0.001*
	1992	1995	1.8585 (1.4238, 2.2950)	< 0.001*		
	1995	2000	1.1751 (1.0389, 1.3114)	< 0.001*		
	2000	2011	1.5211 (1.4874, 1.5548)	< 0.001*		
	2011	2021	0.9981 (0.9649, 1.0314)	< 0.001*		
	**DALYs, Rate**					
	**Segment Year Start**	**Segment Year End**	**APC (95%CI)**	**P-value**	**AAPC (95%CI)**	**P-value**
Neonates	1990	1993	0.2256 (0.0312, 0.4203)	0.026*	-0.2229 (-0.2639, -0.1818)	< 0.001*
	1993	2000	0.5119 (0.4463, 0.5776)	< 0.001*		
	2000	2005	-0.7317 (-0.8527, -0.6105)	< 0.001*		
	2005	2009	-1.3062 (-1.4963, -1.1158)	< 0.001*		
	2009	2017	-0.4646 (-0.5161, -0.4131)	< 0.001*		
	2017	2021	0.3731 (0.2512, 0.4952)	< 0.001*		
Early infants	1990	1994	-0.1298 (-0.2482, -0.0113)	0.034	-0.2189 (-0.2584, -0.1793)	< 0.001*
	1994	2000	0.6254 (0.5412, 0.7097)	< 0.001*		
	2000	2005	-0.7156 (-0.8331, -0.5979)	< 0.001*		
	2005	2009	-1.1759 (-1.3599, -0.9915)	< 0.001*		
	2009	2017	-0.3641 (-0.4148, -0.3134)	< 0.001*		
	2017	2021	0.3072 (0.1849, 0.4297)	< 0.001*		
Late infants	1990	1994	-0.4446 (-0.6299, -0.2588)	< 0.001*	-0.2714 (-0.3344, -0.2083)	< 0.001*
	1994	2000	0.5147 (0.3834, 0.6462)	< 0.001*		
	2000	2005	-0.7690 (-0.9534, -0.5842)	< 0.001*		
	2005	2009	-1.0200 (-1.3091, -0.7300)	< 0.001*		
	2009	2018	-0.3077 (-0.3721, -0.2433)	< 0.001*		
	2018	2021	0.3381 (0.0386, 0.6384)	0.029*		
Toddlers	1990	1994	-0.5615 (-0.7518, -0.3708)	< 0.001*	-0.3071 (-0.3828, -0.2314)	< 0.001*
	1994	2000	0.4558 (0.3206, 0.5913)	< 0.001*		
	2000	2010	-0.8213 (-0.8757, -0.7668)	< 0.001*		
	2010	2013	0.0087 (-0.5934, 0.6144)	0.976		
	2013	2018	-0.4812 (-0.6703, -0.2918)	< 0.001*		
	2018	2021	0.2074 (-0.0952, 0.5108)	0.165		
Preschoolers	1990	1993	-0.6454 (-0.921, -0.3691)	< 0.001*	-0.3485 (-0.4086, -0.2883)	< 0.001*
	1993	2000	0.1732 (0.0799, 0.2666)	0.001*		
	2000	2006	-0.5108 (-0.6317, -0.3898)	< 0.001*		

	2006	2010	-0.8797 (-1.1404, -0.6184)	< 0.001*		
	2010	2014	0.0389 (-0.2259, 0.3043)	0.759		
	2014	2021	-0.5185 (-0.5894, -0.4475)	< 0.001*		
Schoolers	1990	2003	-0.0853 (-0.0938, -0.0768)	< 0.001*	-0.2429 (-0.2691, -0.2167)	< 0.001*
	2003	2006	-0.2951 (-0.4574, -0.1325)	0.002*		
	2006	2009	-0.8681 (-1.0329, -0.7029)	< 0.001*		
	2009	2015	-0.0707 (-0.1080, -0.0334)	0.001*		
	2015	2019	-0.4918 (-0.5765, -0.4070)	< 0.001*		
	2019	2021	-0.2648 (-0.4353, -0.094)	0.005*		
Adolescents	1990	1994	0.3178 (0.2393, 0.3964)	< 0.001*	-0.1970 (-0.2309, -0.1630)	< 0.001*
	1994	1997	-0.1357 (-0.3798, 0.1090)	0.255		
	1997	2010	-0.4487 (-0.4631, -0.4342)	< 0.001*		
	2010	2015	-0.0174 (-0.0958, 0.0612)	0.644		
	2015	2019	-0.3100 (-0.4336, -0.1862)	< 0.001*		
	2019	2021	0.1003 (-0.1454, 0.3467)	0.398		
Adults	1990	1995	0.0424 (0.0024, 0.0824)	0.039*	-0.3537 (-0.3741, -0.3334)	< 0.001*
	1995	2000	-0.5384 (-0.5943, -0.4824)	< 0.001*		
	2000	2005	-0.3272 (-0.3832, -0.2712)	< 0.001*		
	2005	2015	-0.4673 (-0.4835, -0.4511)	< 0.001*		
	2015	2019	-0.5752 (-0.6632, -0.4872)	< 0.001*		
	2019	2021	0.0661 (-0.1098, 0.2423)	0.436		

Abbreviation: APC, annual percentage changes; AAPC, average annual percentage changes; IDD, intellectual developmental disability; CHD, congenital heart disease; CI, confidence intervals; DALYs, disability adjusted life years.

* Statistical significance

eAppendix6. The cost of IDD attributable to CHD in 2021 (STable 8)

STable 8. The cost of IDD attributable to CHD across 204 countries and territories in 2021 (US dollar in 2021)

	Direct cost (million)	Indirect cost (million)	Total cost (million)
**High-income**			
**High-income North America**			
Canada	72.87 (31.05-93.58)	842.83 (528.98-1,235.87)	915.7 (560.03-1,329.45)
Greenland	-	-	-
United States of America	1,177.44 (491.5-1,428.2)	10,067.13 (7,133.32-13,657.21)	11,244.56 (7,624.83-15,085.41)
**Australasia**			
Australia	44.39 (15.52-62.02)	392.46 (188.34-661.49)	436.85 (203.86-723.51)
New Zealand	7.42 (2.97-9.63)	71.89 (34.97-114.82)	79.31 (37.94-124.45)
**High-income Asia Pacific**			
Brunei Darussalam	0.02 (0-0.08)	0.6 (0-2.25)	0.62 (0-2.33)
Japan	56.21 (1.63-119.22)	621 (1.9-1,442.82)	677.2 (3.53-1,562.04)
Singapore	0.75 (0-4.75)	12.25 (0-66.75)	13 (0-71.5)
Republic of Korea	7.38 (0-26.72)	83.87 (0-339.16)	91.25 (0-365.88)
**Western Europe**			
Andorra	0.05 (0.02-0.07)	-	-
Austria	20.24 (7.83-24.51)	265.01 (115.4-400.37)	285.25 (123.23-424.89)
Belgium	21.01 (9.95-26.24)	356 (165.68-574.9)	377.01 (175.63-601.14)
Cyprus	1.43 (0.76-1.8)	22.38 (11.33-33.26)	23.81 (12.08-35.06)
Denmark	5.08 (0.34-9.37)	29.26 (4.93-83.61)	34.34 (5.28-92.98)
Finland	10.74 (5.47-13.54)	190.14 (95.49-293.38)	200.88 (100.96-306.92)
France	164.13 (95.9-208.1)	2,915.81 (1,510.67-5,015.41)	3,079.94 (1,606.57-5,223.5)
Germany	148.65 (29.62-201.9)	1,346.84 (230.77-2,570.8)	1,495.49 (260.39-2,772.7)
Greece	4.92 (2.63-5.95)	173.97 (82.73-267.04)	178.89 (85.36-273)
Iceland	0.67 (0.21-0.87)	6.13 (2.02-10.55)	6.8 (2.23-11.42)
Ireland	9.72 (2.77-13.16)	151.35 (36.87-289.52)	161.07 (39.64-302.68)
Israel	20.27 (10.26-24.94)	333.09 (168.79-491.62)	353.36 (179.06-516.55)
Italy	51.9 (25.52-64.14)	1,639.38 (840.43-2,432.95)	1,691.28 (865.94-2,497.09)
Luxembourg	1.3 (0.45-1.75)	29.06 (11.35-49.19)	30.36 (11.8-50.95)
Malta	0.45 (0.2-0.56)	7.76 (2.41-13.31)	8.22 (2.61-13.87)
Monaco	0.04 (0-0.08)	-	-
Netherlands	26.36 (7.78-34.96)	253.99 (70.19-470.9)	280.35 (77.97-505.85)
Norway	14.84 (6.28-18.91)	183.06 (90.76-276.53)	197.9 (97.05-295.44)
Portugal	7.36 (4.1-9.13)	169.57 (89.61-259.97)	176.93 (93.71-269.11)
San Marino	0.03 (0.01-0.04)	-	-
Spain	58.76 (48.87-67.39)	2521.27 (1766.73-3577.23)	2580.03 (1815.6-3644.62)
Sweden	24.45 (11.69-31.7)	312.24 (195.23-460.02)	336.69 (206.92-491.72)
Switzerland	28.35 (10.48-36.46)	225.28 (88.55-366.6)	253.63 (99.03-403.07)
United Kingdom	114.55 (65.28-137.85)	1,596.51 (966.24-2,254.07)	1,711.06 (1,031.52-2,391.92)
**Southern Latin America**			
Argentina	15.68 (7.86-19.71)	467.28 (311.66-650.6)	482.96 (319.53-670.3)

Chile	9.61 (3.25-11.72)	265.12 (149.78-374.79)	274.73 (153.04-386.5)
Uruguay	1.7 (0.73-2.15)	46.59 (30.07-67.19)	48.29 (30.8-69.34)
**Central Europe, eastern Europe, and central Asia**			
**Eastern Europe**			
Belarus	1.76 (0.87-2.2)	99.26 (37-154.57)	101.03 (37.86-156.77)
Estonia	0.94 (0.26-1.22)	18.81 (4.49-37.45)	19.75 (4.75-38.67)
Latvia	1.25 (0.41-1.59)	26.65 (8.23-50.1)	27.9 (8.65-51.69)
Lithuania	1.69 (0.5-2.15)	39.85 (10.25-79.78)	41.54 (10.75-81.92)
Republic of Moldova	0.54 (0.44-0.65)	21.96 (10.69-32.85)	22.5 (11.13-33.5)
Russian Federation	44.25 (18.67-54.95)	1,836.16 (670.5-2,941.14)	1,880.41 (689.17-2,996.09)
Ukraine	5.62 (4.15-6.7)	350.18 (168.22-490.89)	355.8 (172.37-497.59)
**Central Europe**			
Albania	0.46 (0.29-0.56)	19.37 (6.98-27.74)	19.83 (7.26-28.3)
Bosnia and Herzegovina	0.79 (0.45-0.97)	28.96 (9.07-45.71)	29.75 (9.52-46.68)
Bulgaria	2.56 (1.05-3.21)	76.5 (19.45-131.56)	79.07 (20.51-134.77)
Croatia	1.61 (0.47-2.06)	46.08 (13.53-98.78)	47.69 (14-100.84)
Czechia	9.11 (2.03-12.01)	146.57 (32.45-310.81)	155.69 (34.48-322.83)
Hungary	4.02 (1.01-5.16)	96.85 (19.68-207.37)	100.87 (20.69-212.53)
North Macedonia	0.42 (0.21-0.53)	19.92 (6.44-31.29)	20.34 (6.65-31.82)
Montenegro	0.21 (0.08-0.26)	5.92 (1.55-10.69)	6.13 (1.63-10.95)
Poland	16.66 (5.6-20.61)	696.51 (212.68-1,141.37)	713.17 (218.28-1,161.98)
Romania	5.74 (1.89-7.19)	295.93 (57.69-497.48)	301.67 (59.59-504.68)
Serbia	2.29 (1.17-2.85)	70.19 (20.06-108.09)	72.48 (21.23-110.94)
Slovakia	2.96 (0.78-3.78)	64.39 (13.8-125.87)	67.35 (14.58-129.65)
Slovenia	1.69 (0.4-2.2)	26.24 (5.83-56.08)	27.93 (6.24-58.27)
**Central Asia**			
Armenia	1.18 (0.79-1.44)	42.35 (14.3-58.74)	43.53 (15.09-60.18)
Azerbaijan	1.59 (0.85-2.01)	148.67 (44.16-215.37)	150.26 (45.01-217.38)
Georgia	0.85 (0.62-1.01)	52.18 (22.13-70.07)	53.03 (22.75-71.09)
Kazakhstan	6.28 (2.42-7.98)	512.64 (146.22-804.88)	518.92 (148.64-812.86)
Kyrgyzstan	0.54 (0.43-0.66)	59.57 (32.14-103.12)	60.11 (32.57-103.78)
Mongolia	1.23 (0.84-1.53)	72.59 (28.27-102.56)	73.83 (29.11-104.09)
Tajikistan	1 (0.79-1.24)	128.05 (63.89-228.84)	129.05 (64.68-230.08)
Turkmenistan	2.24 (1.02-2.81)	121.74 (42.59-180.75)	123.98 (43.61-183.57)
Uzbekistan	5.75 (4.21-7.11)	483.72 (211.65-707.35)	489.47 (215.87-714.45)
**Latin America and Caribbean**			
**Central Latin America**			
Colombia	10.07 (4.22-12.84)	299.7 (71.86-547.29)	309.77 (76.08-560.13)
Costa Rica	1.76 (0.66-2.3)	44.02 (10.34-80.79)	45.78 (11-83.1)
El Salvador	1.55 (1.05-1.94)	40.46 (12.02-64.02)	42.01 (13.08-65.96)
Guatemala	2.61 (1.95-3.21)	107.74 (25.17-176.14)	110.35 (27.12-179.35)
Honduras	1.56 (1.19-1.88)	50.19 (13.07-71.1)	51.74 (14.26-72.98)
Mexico	34.49 (13.72-41.93)	1,243.31 (398.14-1,986.44)	1,277.8 (411.86-2,028.38)
Nicaragua	0.69 (0.52-0.83)	29.74 (8.97-41.83)	30.43 (9.49-42.65)
Panama	2.49 (0.64-3.31)	59.75 (11.87-121.46)	62.24 (12.51-124.77)

Venezuela (Bolivarian Republic of)	1.97 (0.93-2.48)	-	-
**Andean Latin America**			
Bolivia (Plurinational State of)	1.77 (1.39-2.11)	64.53 (25.84-85.6)	66.3 (27.23-87.71)
Ecuador	4.63 (3.23-5.62)	142.02 (45.26-206.1)	146.66 (48.5-211.72)
Peru	7.52 (4.69-9.22)	250.03 (75.07-376.92)	257.56 (79.76-386.14)
**Caribbean**			
Antigua and Barbuda	0.02 (0.01-0.03)	-	-
Bahamas	0.2 (0.06-0.26)	2.94 (0.49-5.7)	3.14 (0.55-5.96)
Barbados	0.11 (0.05-0.14)	1.56 (0.39-2.79)	1.68 (0.44-2.93)
Belize	0.06 (0.04-0.07)	2.61 (0.59-4)	2.67 (0.63-4.06)
Bermuda	-	-	-
Cuba	-	-	-
Dominica	0.01 (0.01-0.01)	-	-
Dominican Republic	1.65 (0.74-2.04)	74.17 (15.09-139.85)	75.82 (15.83-141.89)
Grenada	0.02 (0.01-0.02)	-	-
Guyana	0.17 (0.13-0.21)	12.99 (2.61-20.42)	13.16 (2.74-20.63)
Haiti	0.5 (0.4-0.62)	36.82 (10.68-66.49)	37.32 (11.08-67.11)
Jamaica	0.34 (0.23-0.43)	8.79 (1.86-14.16)	9.14 (2.1-14.59)
Puerto Rico	-	33.03 (5.61-72.26)	-
Saint Kitts and Nevis	0.02 (0.01-0.02)	NA (NA-NA)	-
Saint Lucia	0.03 (0.01-0.03)	0.9 (0.12-1.45)	0.93 (0.14-1.48)
Saint Vincent and the Grenadines	0.02 (0.01-0.02)	0.78 (0.16-1.25)	0.79 (0.17-1.28)
Suriname	0.06 (0.03-0.07)	4.6 (0.84-7.69)	4.65 (0.87-7.76)
Trinidad and Tobago	0.43 (0.08-0.55)	7.6 (1.29-19.82)	8.03 (1.36-20.37)
United States Virgin Islands	-	-	-
**Tropical Latin**			
Brazil	63.87 (42.81-76.1)	1,611.93 (574.45-2,390.56)	1,675.8 (617.25-2,466.66)
Paraguay	1.75 (1.19-2.18)	58.66 (16.44-88.43)	60.42 (17.63-90.61)
**Southeast Asia, east Asia, and Oceania**			
**East Asia**			
China	292.86 (184.1-352.05)	9,315.27 (4,461.09-13,452.92)	9,608.12 (4,645.19-13,804.97)
Democratic People's Republic of Korea	-	-	-
Taiwan (Province of China)	-	-	-
**Southeast Asia**			
Cambodia	1.33 (1.07-1.6)	90.5 (49.37-137.05)	91.83 (50.44-138.66)
Indonesia	20.34 (16.85-24.15)	2,250.67 (1,264.88-3,377.13)	2,271.01 (1,281.73-3,401.28)
Lao People's Democratic Republic	0.36 (0.29-0.44)	62.43 (32-90.2)	62.79 (32.29-90.64)
Malaysia	5.93 (3.59-7.42)	381.81 (124.99-583.36)	387.74 (128.57-590.78)
Maldives	0.17 (0.12-0.21)	3.3 (1.31-4.78)	3.47 (1.43-4.98)
Mauritius	0.2 (0.13-0.25)	9.92 (3.65-15.08)	10.11 (3.78-15.33)
Myanmar	2.34 (1.88-2.85)	285.67 (139.25-411.71)	288 (141.13-414.55)
Philippines	13.39 (11.06-15.74)	904.55 (493.51-1,381.55)	917.95 (504.57-1,397.29)
Sri Lanka	1.3 (0.98-1.59)	175.87 (80.16-260.43)	177.18 (81.14-262.03)
Seychelles	0.03 (0.02-0.03)	-	-
Thailand	6.75 (5.61-8.13)	454.27 (274.1-708.17)	461.02 (279.71-716.3)

Timor-Leste	0.14 (0.11-0.17)	10.63 (5.11-16.41)	10.77 (5.23-16.59)
Viet Nam	6.69 (4.35-8.18)	546.37 (259.81-856.27)	553.05 (264.15-864.45)
**Oceania**			
American Samoa	-	-	-
Cook Islands	-	-	-
Micronesia (Federated States of)	0.02 (0.02-0.03)	-	-)
Fiji	0.15 (0.11-0.19)	6.41 (1.75-9.46)	6.56 (1.86-9.65)
Guam	-	-	-
Kiribati	0.02 (0.02-0.03)	-	-
Marshall Islands	0.03 (0.02-0.03)	-	-
Nauru	0.01 (0.01-0.02)	-	-
Niue	-	-	-
Northern Mariana Islands	-	-	-
Palau	0.01 (0.01-0.01)	-	-
Papua New Guinea	0.51 (0.41-0.63)	55.01 (21.71-96.41)	55.52 (22.12-97.04)
Samoa	0.04 (0.03-0.05)	2.04 (1.07-3.25)	2.08 (1.1-3.3)
Solomon Islands	0.06 (0.05-0.07)	2.22 (1.05-3.4)	2.28 (1.1-3.48)
Tokelau	-	-	-
Tonga	0.02 (0.02-0.03)	0.9 (0.5-1.38)	0.92 (0.52-1.41)
Tuvalu	0.01 (0.01-0.01)	-	-
Vanuatu	0.03 (0.03-0.04)	1.51 (0.78-2.52)	1.55 (0.8-2.56)
**North Africa and Middle East**			
Afghanistan	3.85 (3.21-4.73)	505.37 (356.95-773.59)	509.22 (360.16-778.32)
Algeria	7.48 (5.95-9.27)	1,698.88 (836.3-2,536.25)	1,706.36 (842.25-2,545.51)
Bahrain	0.77 (0.61-0.94)	54.46 (29.34-71.06)	55.23 (29.94-72)
Egypt	16.35 (13.36-19.35)	4,781.4 (2,268.09-6,203.38)	4,797.76 (2,281.44-6,222.73)
Iran (Islamic Republic of)	12.08 (9.88-14.16)	2,256.88 (1,264.65-3,214.76)	2,268.96 (1,274.53-3,228.92)
Iraq	7.47 (6.06-9.05)	1,266.76 (727.24-2,177)	1,274.23 (733.3-2,186.05)
Jordan	2.8 (2.19-3.47)	317.83 (176.63-490.24)	320.63 (178.82-493.71)
Kuwait	4.09 (3.19-4.97)	167.61 (74.36-225.23)	171.7 (77.55-230.2)
Lebanon	1.04 (0.83-1.28)	107.02 (61.98-164.68)	108.07 (62.8-165.97)
Libya	0.99 (0.79-1.19)	125.88 (59.38-181.33)	126.87 (60.16-182.52)
Morocco	4.76 (3.84-5.61)	576.91 (283.88-775.49)	581.67 (287.71-781.1)
Palestine	-	-	-
Oman	2.35 (1.93-2.79)	178.07 (81.3-283.94)	180.42 (83.24-286.74)
Qatar	2.25 (1.25-2.83)	127.37 (32.44-202.44)	129.62 (33.69-205.27)
Saudi Arabia	26.22 (19.94-31.67)	1,704.66 (594.83-2,345.25)	1,730.88 (614.77-2,376.92)
Sudan	0.9 (0.75-1.05)	693.93 (514.86-1,035.67)	694.83 (515.62-1,036.72)
Syrian Arab Republic	0.23 (0.18-0.27)	185.92 (103.52-238.54)	186.14 (103.71-238.81)
Tunisia	2.02 (1.58-2.45)	269.85 (143.42-383.74)	271.87 (145-386.19)
Turkey	18.99 (15.7-22.58)	2,596.51 (1,213.45-4,688.85)	2,615.49 (1,229.15-4,711.43)
United Arab Emirates	7.31 (5.69-8.93)	278.25 (111.88-392.8)	285.55 (117.57-401.73)
Yemen	1.21 (1.01-1.43)	-	-
**South Asia**			
Bangladesh	4.28 (3.17-5.32)	547.23 (94.59-921.23)	551.51 (97.76-926.55)

Bhutan	0.04 (0.03-0.05)	7.06 (4.54-11.74)	7.1 (4.58-11.79)
India	66.92 (56.37-78.52)	22,148.47 (16,627.36-27,937.39)	22,215.38 (16,683.73-28,015.91)
Nepal	1.28 (1.05-1.57)	321.19 (203.14-457.72)	322.48 (204.2-459.28)
Pakistan	9.13 (7.48-11.27)	3,495.61 (2,489.84-5,214.32)	3,504.74 (2,497.32-5,225.59)
**Sub-Saharan Africa**			
**Southern sub-Saharan Africa**			
Botswana	0.54 (0.08-0.72)	15.85 (2.06-36.58)	16.39 (2.14-37.29)
Lesotho	0.15 (0.1-0.18)	4.95 (1.31-7.73)	5.09 (1.41-7.91)
Namibia	0.54 (0.13-0.69)	13.38 (2.41-29.01)	13.92 (2.54-29.7)
South Africa	13.44 (3.45-16.82)	437.06 (103.39-770.41)	450.49 (106.84-787.24)
Eswatini	0.23 (0.08-0.28)	15.17 (3.53-25.98)	15.4 (3.61-26.26)
Zimbabwe	0.83 (0.63-1.05)	61.47 (18.03-87.01)	62.29 (18.66-88.06)
**Western sub-Saharan Africa**			
Benin	0.45 (0.32-0.56)	50.85 (13.19-73.52)	51.3 (13.51-74.08)
Burkina Faso	1.46 (1.11-1.84)	125.75 (40.71-211.97)	127.21 (41.83-213.81)
Cameroon	1.97 (1.2-2.47)	174.03 (52.3-248.89)	175.99 (53.5-251.36)
Cabo Verde	0.06 (0.03-0.08)	2.54 (0.54-3.93)	2.6 (0.57-4.01)
Chad	0.87 (0.65-1.09)	95.75 (33.31-158.84)	96.62 (33.96-159.93)
Côte d’Ivoire	2.09 (1.22-2.64)	155.74 (34.41-253.92)	157.83 (35.63-256.56)
Gambia	0.05 (0.04-0.06)	9.93 (2.62-14.99)	9.98 (2.66-15.05)
Ghana	2.57 (1.2-3.21)	205.46 (46.79-315.27)	208.04 (47.99-318.48)
Guinea	0.6 (0.42-0.77)	84.91 (25.37-131.12)	85.52 (25.79-131.89)
Guinea-Bissau	0.14 (0.11-0.18)	7.15 (2.02-10.32)	7.29 (2.13-10.5)
Liberia	0.42 (0.33-0.52)	9.02 (4.29-14.35)	9.44 (4.62-14.87)
Mali	0.83 (0.61-1.07)	103.63 (26.33-163.4)	104.46 (26.94-164.47)
Mauritania	0.22 (0.12-0.28)	32.28 (4.77-54.13)	32.5 (4.88-54.4)
Niger	1.18 (0.92-1.5)	78.65 (30-142)	79.83 (30.92-143.5)
Nigeria	16.25 (7.09-20.24)	1,051.2 (362.73-1,600.77)	1,067.45 (369.83-1,621)
São Tomé and Príncipe	0.02 (0.02-0.03)	2.86 (0.71-4.33)	2.89 (0.73-4.36)
Senegal	0.89 (0.56-1.11)	80.26 (17.8-124.02)	81.15 (18.36-125.13)
Sierra Leone	0.37 (0.29-0.47)	41.16 (14.47-71.24)	41.54 (14.76-71.71)
Togo	0.36 (0.28-0.44)	28.38 (10.76-38.78)	28.75 (11.05-39.23)
**Eastern sub-Saharan Africa**			
Burundi	0.29 (0.23-0.36)	16 (8.54-29)	16.29 (8.77-29.36)
Comoros	0.04 (0.03-0.05)	2.21 (0.54-3.3)	2.25 (0.56-3.34)
Djibouti	0.06 (0.03-0.07)	11.07 (2.86-17.35)	11.13 (2.89-17.42)
Eritrea	0.13 (0.1-0.16)	-	-
Ethiopia	2.17 (1.74-2.69)	296.31 (129.15-478.99)	298.48 (130.89-481.67)
Kenya	2.89 (2.02-3.46)	223.76 (100.82-298.05)	226.65 (102.84-301.51)
Madagascar	0.36 (0.28-0.44)	36.14 (12.68-57.53)	36.5 (12.96-57.97)
Malawi	0.68 (0.55-0.84)	37.69 (16.13-63.96)	38.37 (16.68-64.8)
Mozambique	1.2 (0.95-1.48)	50.29 (19.07-69.73)	51.49 (20.01-71.21)
Rwanda	0.55 (0.44-0.68)	46.4 (21.16-62.2)	46.95 (21.59-62.88)
Somalia	0.42 (0.34-0.52)	270.08 (177.15-416.73)	270.5 (177.49-417.25)
South Sudan	0.28 (0.19-0.36)	-	-

United Republic of Tanzania	1.53 (1.12-1.88)	144.11 (32.6-209.79)	145.64 (33.72-211.68)
Uganda	1.87 (1.38-2.26)	123.64 (39.61-168.63)	125.51 (40.99-170.89)
Zambia	1.04 (0.69-1.31)	72.97 (18-110.14)	74.01 (18.69-111.44)
**Central sub-Saharan Africa**			
Angola	2.02 (0.79-2.58)	230.02 (45.83-416.83)	232.04 (46.62-419.4)
Central African Republic	0.28 (0.22-0.35)	12.62 (4.47-22.55)	12.9 (4.69-22.9)
Congo	0.26 (0.13-0.32)	31.41 (7.15-47.75)	31.67 (7.28-48.07)
Democratic Republic of the Congo	1.82 (1.42-2.27)	198.05 (85.75-379.24)	199.87 (87.17-381.51)
Equatorial Guinea	0.16 (0.02-0.22)	7.25 (1.27-19.69)	7.41 (1.29-19.92)
Gabon	0.19 (0.04-0.25)	18.11 (3.46-40.33)	18.3 (3.5-40.58)
